# Venom-derived peptides for breaking through the glass ceiling of drug development

**DOI:** 10.3389/fchem.2024.1465459

**Published:** 2024-09-26

**Authors:** Lou Freuville, Chloé Matthys, Loïc Quinton, Jean-Pierre Gillet

**Affiliations:** ^1^ Laboratory of Mass Spectrometry, MolSys Research Unit, University of Liège, Liège, Belgium; ^2^ Laboratory of Molecular Cancer Biology, URPhyM, NARILIS, University of Namur, Namur, Belgium

**Keywords:** venomics, venom peptides, biologically active molecules, drug development, therapy, cancer

## Abstract

Venoms are complex mixtures produced by animals and consist of hundreds of components including small molecules, peptides, and enzymes selected for effectiveness and efficacy over millions of years of evolution. With the development of venomics, which combines genomics, transcriptomics, and proteomics to study animal venoms and their effects deeply, researchers have identified molecules that selectively and effectively act against membrane targets, such as ion channels and G protein-coupled receptors. Due to their remarkable physico-chemical properties, these molecules represent a credible source of new lead compounds. Today, not less than 11 approved venom-derived drugs are on the market. In this review, we aimed to highlight the advances in the use of venom peptides in the treatment of diseases such as neurological disorders, cardiovascular diseases, or cancer. We report on the origin and activity of the peptides already approved and provide a comprehensive overview of those still in development.

## 1 Introduction

**FIGURE 1:**
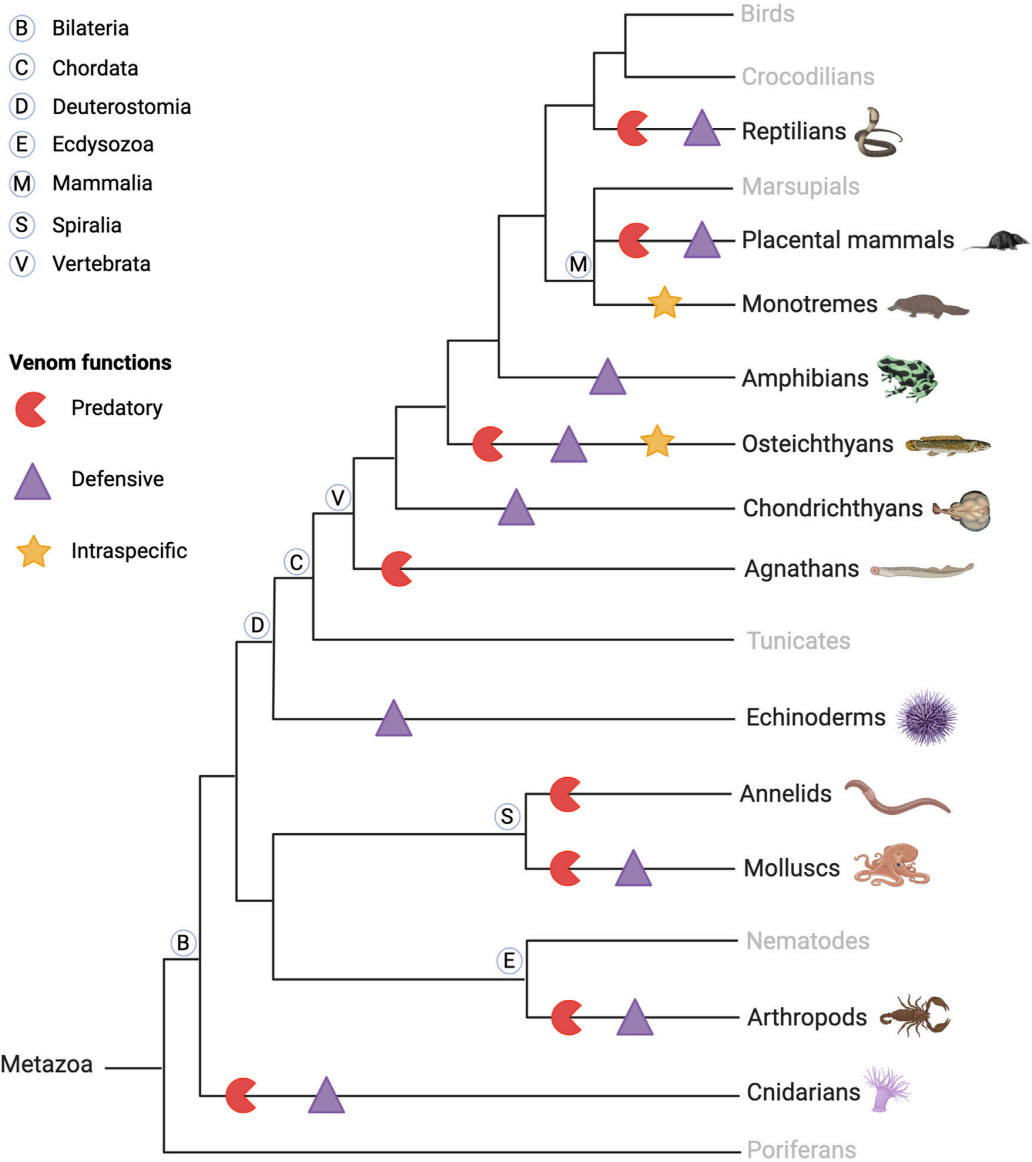
Schematic phylogenetic tree of venomous animal diversity and key venom functions. Venomous animals are found in invertebrates and vertebrates, aquatic and terrestrial animals, and predators and prey. They use their venom for predation and defense, and some use their venom against conspecifics, intraspecifically, in competition for reproduction, as in the platypus. Created with BioRender.com (2024) and inspired by Schendel et al. (2019).

Venomous animals are widely distributed taxonomically, represented in both invertebrates (annelids, arthropods, mollusks, nematodes, cnidarians … ) and vertebrates (platypus, snakes, lizards, fish, shrews … ), as shown in Figure 1 (Morsy et al., 2023). Venomous species are ubiquitous, having colonized many aquatic and terrestrial biotopes, in temperate, tropical, and equatorial areas. Fry *et al.* have defined venom as “a secretion, produced in a specialized gland in an animal, and delivered to a target animal through the infliction of a wound” (Fry et al., 2009). However, in addition to predation and defense, venom serves various functional roles including communication, mating, and offspring care (Schendel et al., 2019). Animal venoms are complex chemical cocktails composed of hundreds of molecules, mostly peptides, and proteins, but also small molecules and salts. Peptides and proteins from venoms are commonly referred to as toxins, but enzymes have also been identified among them (Simoes-Silva et al., 2018). The main families of venom enzymes are L-amino oxidases (LAAOs), phospholipases A2 (PLA2s), proteinases (especially snake venom metalloproteinases (SVMPs), and snake venom serine proteinases (SVSPs) in snake venoms), acetylcholinesterases, and hyaluronidases. Even if they have a deleterious effect on the prey, such enzymes are not (always) considered to be toxins (Utkin, 2015).

Venomous animals have evolved highly complex venoms over millions of years of evolution. Based on recent transcriptomic and proteomic studies, it is generally accepted that an average of a few hundred toxins are present in each venom. Knowing that hundreds of thousands of venomous species have been identified to date, animal venoms can be seen as an incredibly diverse molecular toolbox composed of tens of millions of bioactive peptides and proteins (Herzig et al., 2020). Biologically active venom peptides and proteins act selectively and efficiently against molecular targets, such as ion channels (ICs) and G protein-coupled receptors (GPCRs) but also enzyme-linked receptors (Ghosh et al., 2019; Utkin et al., 2019). Venom toxins have the incredible ability to activate, inhibit, or modulate their functions paving the way for the elucidation of critical physiological processes. From a molecular structural point of view, venom toxins are mostly highly structured peptides with disulfide bridges, providing the molecule with the perfect conformation to bind to the receptors and conferring high stability and resistance to proteases (King, 2013).

Venoms exhibit high chemical diversity and exert a wide range of pharmacological activities. Their toxins act at low doses with high selectivity for receptors, and even for specific subtypes of receptors. From this simple point of view, toxins are extremely attractive for developing therapeutic drugs. However, some of them are not as selective and may target not only a single (sub)type of receptor but a variety of them. In that context, the development of innovative pharmacological drugs appears tricky, if not possible, as the new tool acts on the receptor of interest, without activating additional receptors that may be involved in critical physiological functions (Smith et al., 2015). As these requirements are never met, such polypharmacological toxins are usually discarded from the pool of interest. In the development of toxin-based therapies, the blood-brain barrier passage remains another major obstacle that researchers are actively trying to overcome. It has been shown that adding positively charged amino acids to the terminal ends of the peptide improves its delivery to the target (Teesalu et al., 2009). Although such modification generally reduces potency, it increases the half-life of the active venom-derived peptide drugs. Another critical parameter to explore is oral bioavailability, which depends on the mass and hydrophobicity of the drug candidates. This is not necessarily limiting, depending on the target site of action. Intravenous or local administration is a credible option to circumvent this difficulty (Stepensky, 2018). While toxins can be valuable in therapeutic research, understanding their pharmacological properties, including pharmacokinetics and pharmacodynamics is critical to the successful development of a lead peptide.

Despite these challenges, the potential for venom peptides in developing therapeutic molecules remains significant. Therefore, venom peptides are ideal candidates for developing novel therapeutic molecules due to their high potency, selectivity, and stability (Holford et al., 2018). To date, eleven venom-derived molecules have been approved and marketed for the treatment of disease from lizard (exenatide and lixisenatide), cone snail (ziconotide), leech (bivalirudin and desirudin), and snake (captopril, enalapril, tirofiban, eptifibatide, batroxobin, and cobratide) venoms. These drugs are used: as anticoagulants for bivariludin and desirudin, as antithrombotics for eptifibatide and tirofiban, as defibrinogenating agents for batroxobin, in case of hypertension for captopril and enalapril, to reduce pain for cobratide and ziconotide, and to treat type 2 diabetes for exenatide and lixisenatide. These drugs are synthetic toxins or molecules derived from natural toxins (Bordon et al., 2020). Many studies are underway in preclinical and clinical settings for treating chronic pain, certain cancers, depression, or diabetes (Miljanich, 2004; Mamelak et al., 2006; Osteen et al., 2016). In this context, this review proposes a journey in the recent advances of venom toxins exploited in potential treatments for both cancer and non-cancer diseases. For the reader’s information, this review will not discuss the antimicrobial activity of toxins. More information can be obtained from the review *Antimicrobials from Venomous Animals: An Overview* by Yacoub et al. (2020).

## 2 Snakes: pioneers in the use of venom toxins as medicine. What’s next?

Venomous snakes cause up to 2.7 million cases of envenomation worldwide each year (WHO, 2021). Venom toxins and enzymes disrupt the victim’s physiological systems and cause morbidity or even death if left untreated. The therapeutic use of snake venom was documented in Ayurveda as early as the 7th century and was also mentioned by ancient Greek philosophers and physicians for its pharmacological properties (King, 2013). More recently, technological advances have allowed researchers to transform these potentially deadly toxins into life-saving therapeutics. Components of snake venom have shown potential for the development of new drugs, from the development of captopril, the first drug derived from the bradykinin-potentiating peptide of *Bothrops jararaca* (southeast coast of South America), to disintegrins with potent activity against certain types of cancer. Snake venom exhibits cytotoxic, neurotoxic, and hemotoxic activities, making it the focus of many research projects. The study of the cytotoxic properties of venoms for cancer treatment is ongoing. Despite extensive research on the neurotoxicity of snake venoms for neuronal diseases, no drug derived from snake toxin has been marketed for this purpose. Due to the complexity of the neuronal system, this area of research is still in progress (Oliveira et al., 2022).

### 2.1 Composition of snake venoms

The classification proposed by Tasoulis and Isbister in 2017 identifies four primary (see Table 1) and six secondary protein families (see Table 2) (Tasoulis and Isbister, 2017), which can be respectively associated with enzymatic and non-enzymatic bioactivities (Kang et al., 2011). Several toxins and enzymes exhibit species-specific properties, including defensins, waglerin, maticotoxin, and cystatins. In contrast, PLA2 is the most abundant protein family detected in snake venoms and is present in nearly all snake species (Tasoulis and Isbister, 2023). The PLA2s family is followed in prevalence by the three-finger toxins (3FTxs), a family of non-enzymatic toxins named as such due to the three loops formed by the peptide chain constrained by a conserved disulfide-bond pattern. Beyond these general considerations, it is important to keep in mind that the composition of snake venom is highly species-dependent. It is also influenced by the gender, age, geographic area, and feeding habits of the snake (Gopal et al., 2023). For example, elapid venoms consist mainly of PLA2s and 3FTxs. In contrast, viper venoms are mostly devoid of 3FTxs and contain more snake venom metalloproteinases, PLA2s, and snake venom serine proteases. Crotal venoms also lack 3FTxs, except for *Atropoides nummifer* (Tasoulis and Isbister, 2017).

**TABLE 1:** Dominant protein families.

Toxin family names	Information	Examples	References for more details
Phospholipases A2 (PLA2s) Group I *Elapidae* and *Hydropidae*	13–18.5 kDa Enzymatic 7 disulfide bridges Presence of a calcium-binding loop	Notexin (*Notechis scutatus*): 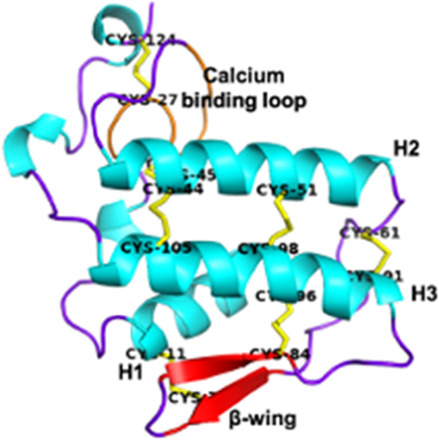 PDB 1AE7	Westerlund et al. (1992) Ponce-Soto et al. (2007) Castro-Amorim et al. (2023)
Phospholipases A2 (PLA2s) Group II *Crotalidae* and *Viperidae*		Crotoxin (*Crotalus durissus*): 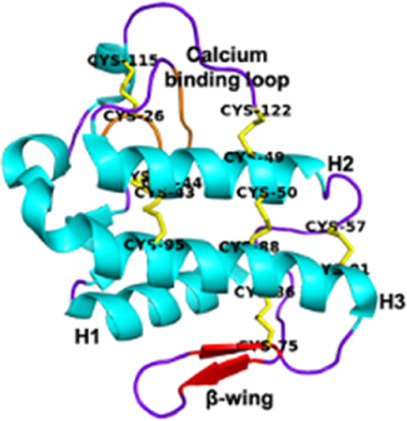 PDB 3R0L	
Snake Venom Metalloproteinases (SVMPs) P-I *Viperidae*	20–30 kDa Enzymatic Metalloproteinase (M) domain only	Adamalysin-II (*Crotalus adamenteus*): 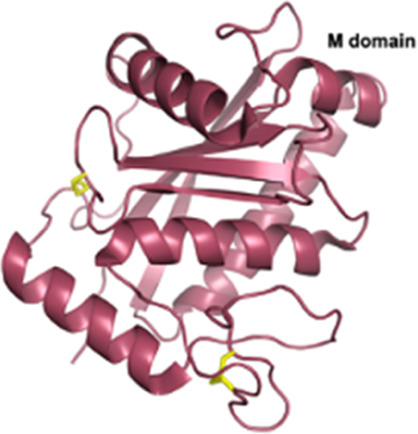 PDB 4AIG	Gomis-Ruth et al. (1993) Igarashi et al. (2007) Olaoba et al. (2020)
Snake Venom Metalloproteinases (SVMPs) P-II *Viperidae*	30–60 kDa Enzymatic Pro-domain, M domain, and disintegrins domain (D) Presence of a RGD (Arg-Gly-Asp) motif	Salmosin (*Gloydius brevicaudus*): 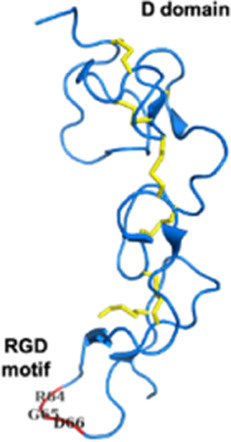 PDB 1L3X	
Snake Venom Metalloproteinases (SVMPs) P-III *Viperidae, Elapidae, Atractaspididae*, and *Colubridae*	60–100 kDa Enzymatic Pro-domain, M domain, D domain and cysteine-rich (C) domain	VAPB2 (*Crotalus atrox*): 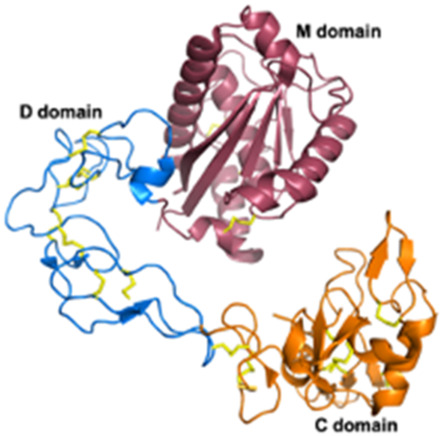 PDB 2DW0	
Snake venom serine proteases (SVSPs) *Viperidae, Elapidae*	26–250 kDa Enzymatic Extend C-terminal tail Active site is constituted of the canonical catalytic triad His-Asp-Ser	Dav-PA (*Deinagkistrodon acutus*): 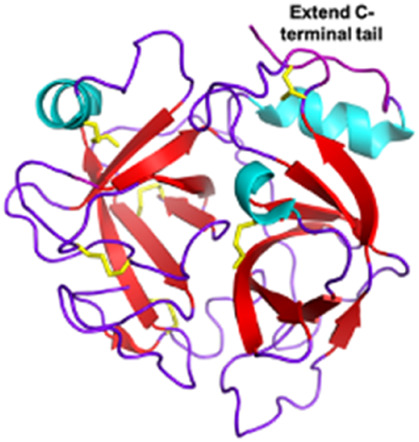 PDB 1OP0	Serrano and Maroun (2005)
Three-finger toxins (3FTxs) *Elapidae*	6–9 kDa Non-enzymatic Named after a highly conserved folding pattern: 3 β-stranded loops (fingers) and a central core stabilized by 4 disulfide bridges Possibility to present a RGD motif	Mambalgin-1 (*Dendroaspis polylepis*): 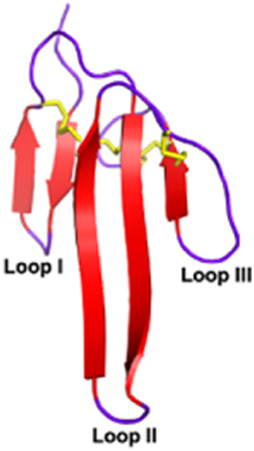 PDB 7ULB	Utkin (2019)

**TABLE 2:** Secondary protein families discussed in the review.

Toxin family names	Information	Examples	References for more details
L-amino acid oxidases (LAAOs) *Viperidae, Elapidae*	Monomeric mass: 50–70 kDa Enzymatic Homodimer with FAD (Flavine Adenine Dinucleotide) or FMN (Flavin Mononucleotide) cofactors	LAAO (*Calloselasma rhodostoma*): 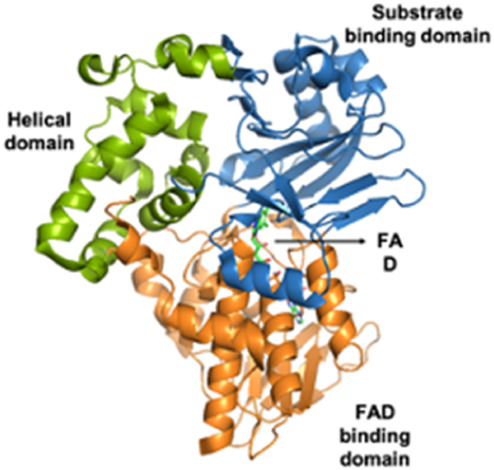 PDB 2IID	Ullah (2020) Izidoro et al. (2014)
Cysteine-rich secretory proteins (CRiSPs) *Viperidae, Elapidae*	20–30 kDa Non-enzymatic Pathogenesis related group 1 (PR-1) domain, a short hinge region and a cysteine rich (CR) domain	CRiSP (*Trimeresurus Stejnegeri*): 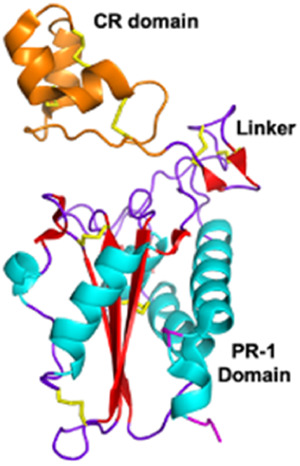 PDB 1RC9	Tadokoro et al. (2020) Urra et al. (2015) Guo et al. (2005)
Disintegrins *Viperidae*, *Atractaspididae* and *Colubridae*	5–10 kDa Non-enzymatic Four subfamilies ranging from 4 to 7 disulfide bridges Result of the proteolytic process of P-II SVMPs	Echistatin (*Echis carinatus*): 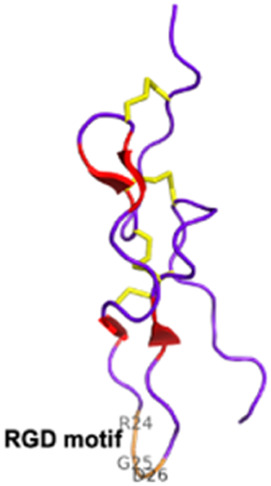 PDB 6LSQ	Chen et al. (2020) Calvete et al. (2005) Vasconcelos et al. (2021)
Natriuretic peptides (NPs) *Viperidae, Elapidae*	5–10 kDa	Bradykinin-potentiating and C-type natriuretic peptides (*Bothrops insularis*): Not available in PDB AFDB accession: AF-P68515-F1	Potter et al. (2009)

### 2.2 Drugs derived from snake toxins currently in clinical use

**FIGURE 2:**
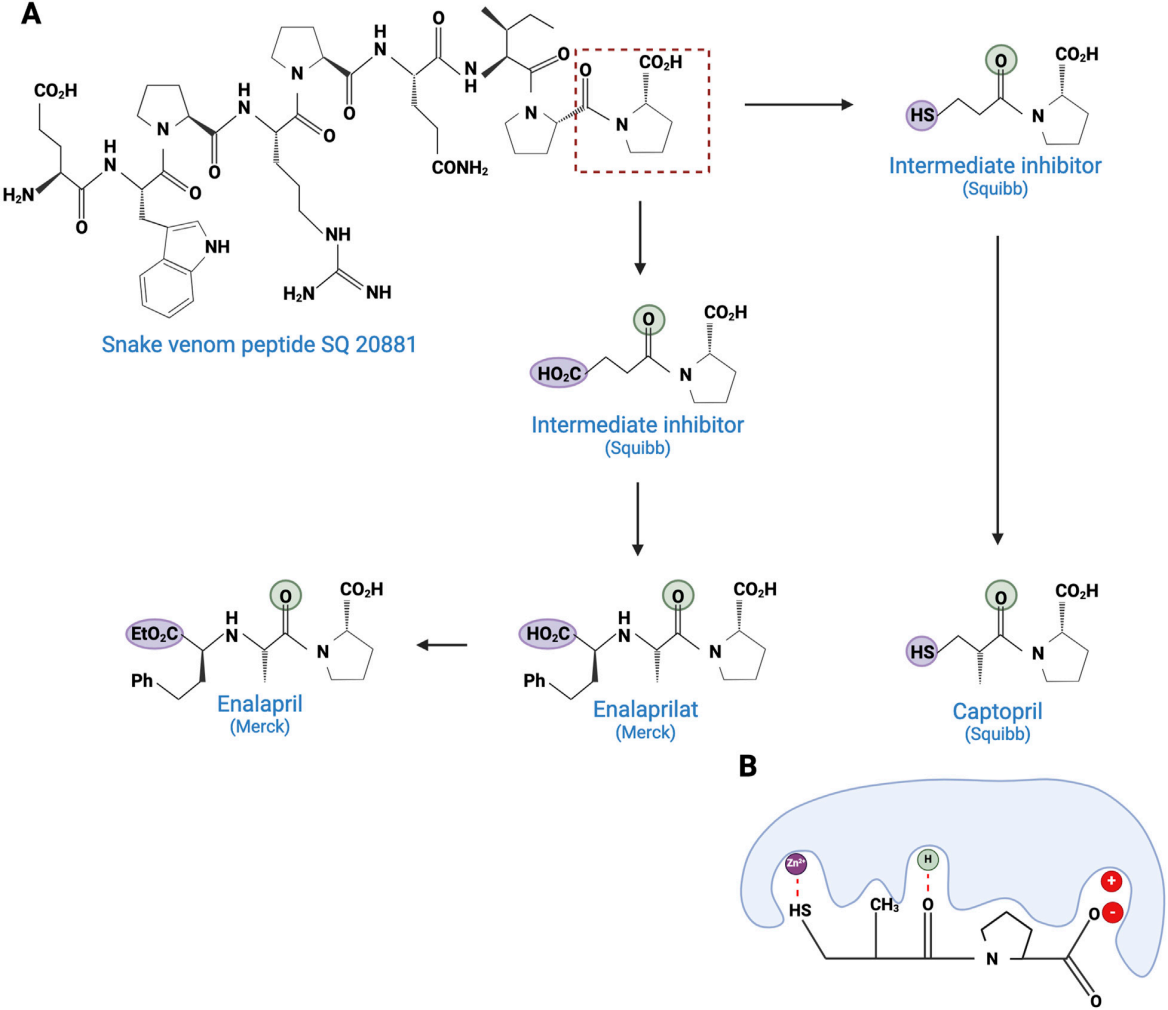
Development of captopril and next ACE inhibitors. **(A)** The natural peptide SQ 20881 (sequence EWPRPQIPP), discovered in *Bothrops jararaca* venom, is an inhibitor of ACE, inducing a drop in blood pressure. Squibb company synthetized a derivative which led, after optimization, to the captopril (1981). Because of the side effects of the thiol group, Merck replaced the latter with a carboxylate, resulting in the enalaprilat molecule (1985). However, as this modification resulted in the loss of oral bioavailability, the esterification of the enalaprilat was considered by Merck to solve the problem successfully and created the enalapril (1985). The conserved zinc binder is shown in purple. The H-bond acceptor is shown in green. **(B)** Interaction between captopril and ACE. By preventing the binding of the inert Angiotensin I to ACE, captopril inhibits the formation of Angiotensin II. Created with BioRender.com (2024).

As early as 1981, the US Food and Drug Administration (FDA) approved the first venom-based treatment, a toxin isolated from the venom of the Brazilian pit viper, *Bothrops jararaca* (see Figure 2). Captopril (Capoten^®^) is a derivative of bradykinin potentiating peptides (BPPs), which lower blood pressure and reduce cardiac hypertrophy (Kini and Koh, 2020). BPPs, which belong to the natriuretic peptide family, inactivate bradykinin and catalyze the conversion of Angiotensin I to the vasoconstrictor Angiotensin II, by inhibiting the proteolytic Angiotensin-Converting Enzyme (ACE) (Ferreira et al., 1970). Captopril mimics the BPP Pro-Ala-Trp triad, the recognition motif for ACE, and binds strongly to the enzyme’s active site (Ki = 1.7 nM) (Cushman and Ondetti, 1991). Captopril is hydrophilic and has a low molecular weight (Stepensky, 2018). However, its main disadvantage is the presence of a thiol group, which has been reported to cause side effects, such as skin rash and loss of taste. To circumvent this drawback, this reactive thiol has been replaced by a carboxylate function in the first step, resulting in a new molecule called enalaprilat. This replacement induced a lack of oral bioavailability, but replacing the carboxylate with an ethylic ester greatly improved it, resulting in enalapril (Patchett, 1984). Based on these initial developments, many drugs have been commercialized (lisinopril, quinapril, ramipril, etc.) (Acharya et al., 2003).

Snake toxins and enzymes have also been described as potent antithrombotic drugs. For instance, echistatin (49 residues) from *Echis carinatus* venom and barbourin (74 residues) from *Sistrurus miliarius* belong to the disintegrin family, due to their potency to bind to integrins. Integrins αIIbβ3 are membrane receptors found on the surface of blood platelets. These receptors play a critical role in platelet aggregation (Casewell et al., 2013). In arterial thrombosis, rupture of the atherosclerotic plaque triggers platelet adhesion and aggregation, leading to clot formation in the arteries, obstructing blood flow to the brain and heart. Ligands for integrins, such as fibronectin, fibrinogen, and von Willebrand factor, interact in the final step of platelet aggregation, via a common recognition motif: a tripeptide sequence RGD (Arg, Gly, Glu) (Lebreton et al., 2016). This motif is commonly found in many PII-type SVMPs. It is unsurprisingly present in echistatin, and a similar sequence, KGD (Lys, Gly, Gluc), is also found in the barbourin. The advantage of the KGD sequence is that it does not block the adhesive functions of other RGD-dependent integrins, and therefore specifically inhibits platelet-dependent thrombus formation. Echistatin and barbourin have led to the development of two αIIbβ3 inhibitors, called tirofiban (Aggrastat^®^) and eptifibatide (Integrilin^®^), respectively, both of which were approved by the FDA in 1998 (Bledzka et al., 2013).

Another target of snake toxins is fibrinogen for anticoagulation purposes (Kini, 2006). For example, consider batroxobin, a 231-residue serine protease isolated from *Bothrops moojeni* venom. Batroxobin is a snake venom thrombin-like serine protease (svTLEs) that catalyzes the cleavage of the Arg16-Gly17 bond of the Aα chain of fibrinogen. By catalyzing this cleavage, it reduces plasma levels of fibrinogen, making clots more fragile and easier to dissolve (Vu et al., 2013). The advantage of this substitute over the well-known thrombin is double as it is more stable and not inhibited by heparin and hirudin (Funk et al., 1971). Treatment with Defibrase^®^, the drug derived from batroxobin and marketed in China and Japan, is used in ischemia caused by vascular occlusive disease, peripheral and microcirculatory dysfunction, and acute cerebral infarction (D'Amelio et al., 2021).

In the venom of the lancehead pit viper, *Bothrops atrox*, an enzymatic system has been discovered and demonstrated a powerful anti-hemorrhagic activity. This system, composed of batroxobin and a thromboplastin-like enzyme, has been derived into a pharmaceutical specialty called Reptilase^®^, which plays the role of haemocoagulase (Oliveira et al., 2022). The thromboplastin-like enzyme is a metalloprotein that activates Factor X to fXa, which converts prothrombin into thrombin. Combining the two activities, forming haemocoagulase, accelerates the hemostasis process, reducing bleeding and clotting times (Pentapharm).

The sixth drug based on a snake venom toxin is α-cobrotoxin (cobratide), purified from the venom of *Naja naja atra*, a cobra found in China. α-Cobrotoxin is a 3FTx α-neurotoxin, known to act selectively and with high affinity on muscle type α1 nicotinic acetylcholine receptors (nAChRs). α-Cobrotoxin exhibits analgesic activity without opiate dependence and can therefore substitute for morphine (Gazerani and Cairns, 2014).

### 2.3 Snake toxins in drug development

Other snake toxins are currently under development in the pharmaceutical field, to play a role in the treatment of cardiovascular diseases as antiplatelet or anticoagulant agents, and potentially even in the treatment of certain types of cancer (Kini and Koh, 2020; da Rocha et al., 2023).

#### 2.3.1 Antiplatelet and anticoagulant agents

As noted above, some drugs have already been developed as antiplatelet agents (tirofiban and eptifibatide). Still, many more toxins with similar activities are being discovered, reinforcing the great potential of toxins used as antiplatelet drugs. Like the drugs already on the market, some 3FTxs share the same RGD motif, which is a key for binding to platelet receptors. This is the case of dendroaspin (also known as mambin) (McDowell et al., 1992), S5C1 (Joubert and Taljaard, 1979), both isolated from the venom of *Dendroaspis jamesoni kaimosae* and thrombostatin from *Dendroaspis angusticeps* (Prieto et al., 2002). They possess the RGD motif in loop III (see Table 1). Dendroaspin targets the most abundant platelet integrin αIIbβ3 and thus prevents the binding of fibrinogen. When the RGD motif is mutated in RYD or RCD, the binding becomes selective for β1 and β3 integrins. Toxin binding inhibits ADP-induced platelet aggregation (Wattam et al., 2001). Another 3FTx, γ-bungarotoxin, isolated from *Bungarus multicinctus*, also shares the RGD motif. In γ-bungarotoxin, the motif is located in loop II, which is less accessible and induces a lower activity for the receptor than in loop III (IC_50_ = 34 µM) (Shiu et al., 2004). All these 3FTxs exhibit anticoagulant properties, making them interesting as potential antithrombotic agents. Other 3FTxs present anticoagulant potential. Hemetexin AB, exactin, and ringhalexin, all from the venom of *Hemachatus haemachatus*, target specific coagulation factors or complexes with inhibitory activity (Banerjee et al., 2005; Barnwal et al., 2016; Girish and Kini, 2016). The disadvantage of exactin and ringhalexin for therapeutic use is that they are also slightly neurotoxic. However, their high selectivity would allow the development of interesting molecular probes or diagnostic tools (Kini and Koh, 2020).

#### 2.3.2 Cytotoxic activity in cancer

**FIGURE 3:**
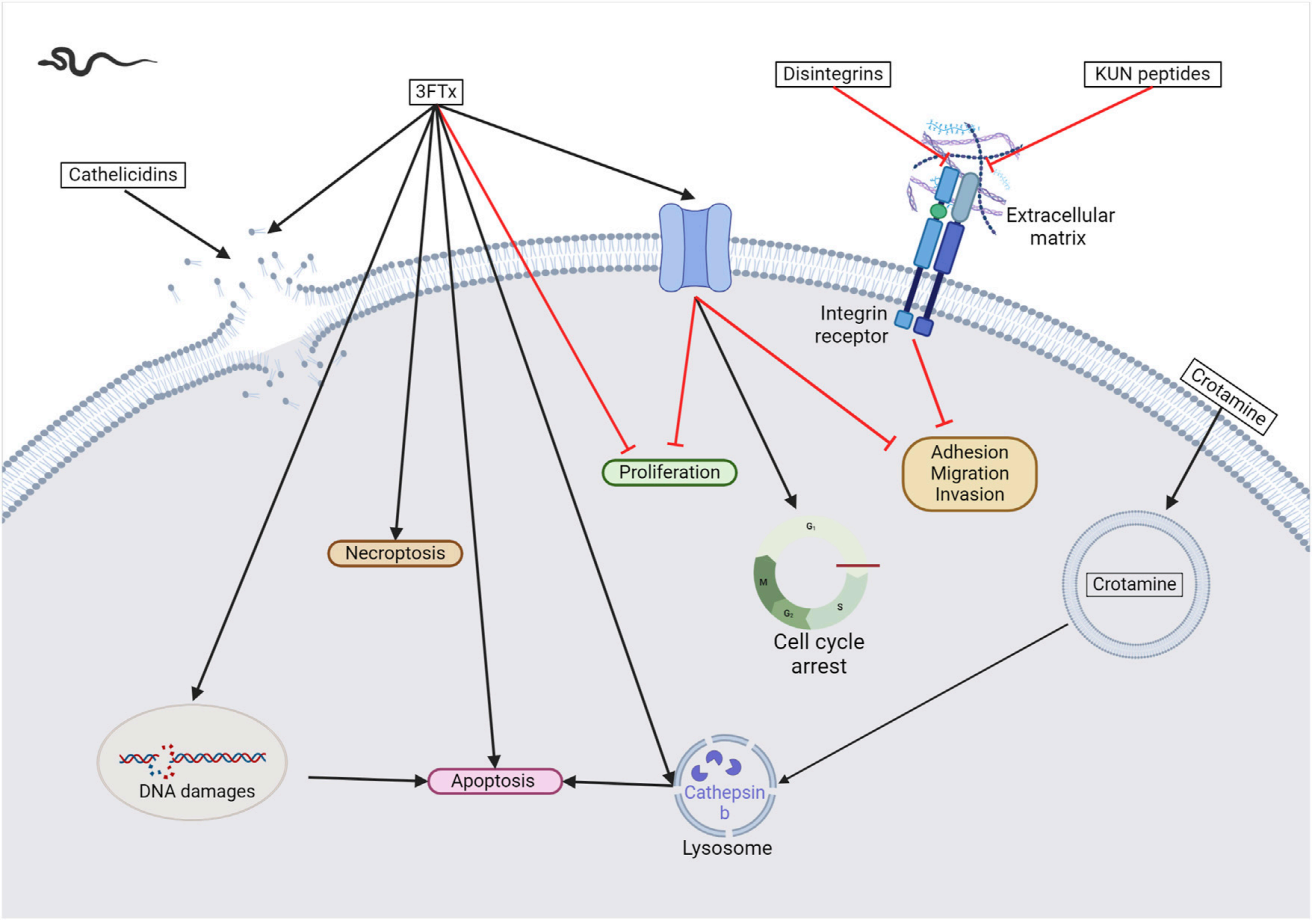
Snake venom toxins as potential anticancer agents. In various cancer cell lines, 3FTx have been demonstrated to have a lytic activity by disrupting cell membranes, inducing DNA damage, and/or the release of lysosomal cathepsin B that further leads to apoptosis, to induce necroptosis and/or to stop cell proliferation (Abdel-Ghani et al., 2019; Wu et al., 2013; Liu et al., 2019). Moreover, these toxins can also target ion channels, halting cancer cell proliferation, adhesion, migration, and invasion, and inducing cell cycle arrest (Bychkov et al., 2021; Sudarikova et al., 2022). Cathelicidins are mainly described to form pores in cancer cell membranes ((Wang et al., 2013). Finally, disintegrins and Kunitz-type serine protease inhibitors (KUN) peptides primarily affect the interactions between the extracellular matrix and cell membrane receptors, reducing cancer cell adhesion, migration, and invasion (Zhou et al., 2000a; Brown et al., 2008; Minea et al., 2010; Zhang et al., 2021; Bhattacharya et al., 2023). Created with BioRender.com (2024).

Snake toxins also present an interest in the cancer field (see Figure 3). Indeed, some 3FTxs are strongly cytotoxic. In that context, cytotoxins have been the focus of numerous studies investigating various cancer cell types. For instance, sumaCTX, a cytotoxin extracted from the venom of *Naja sumatrana*, has received much attention from the community due to its cytotoxic activity against MCF-7 breast cancer cells (Teoh and Yap, 2020). It induces membrane hyperpolarization and apoptosis via activation of the two caspases 3 and 7. Changes in the secretome of cells treated with high doses of sumaCTX were later observed (Hiu and Yap, 2021). Most of the expressed proteins were involved in carbon metabolism, immune response, and necroptosis. *Naja sumatrana* cytotoxin was also tested on two types of cancer cells, lung adenocarcinoma and prostate cancer (Chong et al., 2020). The results showed significant differences in cellular behavior, with an increase in late apoptotic and necrotic cells compared to untreated cells. However, the specific mechanisms involved remain unclear.

Other cytotoxins in snake venoms are being extensively studied for their potential anticancer properties. The NKCT1 (purified *Naja kaouthia* protein toxin) was first extracted from *Naja kaouthia* venom in 2010 and showed cardiotoxic and cytotoxic properties against two leukemia cell lines (U937 and K561) (Debnath et al., 2010). Recently, a growing interest in conjugating this toxin with gold nanoparticles to target leukemia, glioblastoma, hepatocarcinoma, and breast cancer cells emerged (Bhowmik et al., 2013; Bhowmik and Gomes, 2017; Bhowmik et al., 2017). A synergistic effect of administering gold nanoparticle-NKCT1 conjugates was then observed. Indeed, while the conjugation induces apoptosis of cancer cells through caspase activation, it also reduces cytotoxicity against non-cancerous cells. Treatment with GNP-NKCT1 induces autophagy in leukemia cells (Bhowmik and Gomes, 2016). In breast cancer cells, it induces cell cycle arrest by inactivating CDK4 and reduces migration and invasion by inhibiting MMP-9 (Bhowmik and Gomes, 2017).

*Naja atra* cytotoxins (CTX) have also been studied in various cancer cell types. In leukemia cells, CTX1 treatment led to the initiation of necroptosis and the activation of the FasL/Fas-mediated death signaling pathway (Liu et al., 2019; Chiou et al., 2021). Notably, the venom of *Naja atra* contains numerous CTX isoforms, of which only CTX1 can induce this type of cell death whereas CTX3 induces autophagy-dependent apoptosis in leukemia cells (Chiou et al., 2019). This result suggests that different mechanisms may mediate CTX cytotoxicity. In addition, CTX can cause the loss of the lysosomal membrane integrity in breast cancer cells, leading to the release of lysosomal enzymes, including cathepsin B, which induces necrosis or apoptosis (Wu et al., 2013). Intramuscular administration of CTX1 in mice currently results in skeletal muscle necrosis, making its clinical use impossible, without sequence optimization or improved delivery (Ownby et al., 1993).

Another cobra species, *Naja nubiae*, caught the scientific community’s attention by providing the cytotoxin nubein 6.8 (Abdel-Ghani et al., 2019). This peptide shows similarities with other cytotoxins identified in different cobra species and shares the first 6 N-terminal amino acids. In addition, it shows cytotoxic effects against several types of cancer cells, including melanoma and ovarian carcinoma. Cytotoxin nubein 6.8 has been shown to cause DNA damage leading to apoptosis. However, the precise mechanisms underlying this cytotoxicity have not been fully elucidated yet.

NN-32 is a peptide isolated from the cobra *Naja naja* that shows strong homology to other cytotoxins from *Naja* species (Das et al., 2011). When treated with NN-32, leukemia, and breast cancer cells show a reduction in cell viability and proliferation, with a concomitant decrease in lysosomal activity and induction of apoptosis (Das et al., 2013; Attarde and Pandit, 2017). More recently, nanogold particles have been conjugated with the NN-32 peptide, resulting in GNP-NN-32. The goal of developing GNP-NN-32 was the same as for NKCT1, *i.e*., to increase the selective cytotoxicity against breast cancer cells. The results showed lower IC_50_ values (Attarde and Pandit, 2020).

*Dendroaspis polylepis polylepis*, the famous Black mamba, produces mambalgins that inhibit acid-sensing ion channels (ASICs). ASICs are voltage-insensitive receptors that are activated by extracellular protons. By selectively and potently inhibiting the ASIC1a and ASIC1b subtypes, with an IC_50_ between 11 and 300 nM, mambalgin-1 showed a potent analgesic effect, while mambalgin-2 is a powerful and reversible inhibitor of ASIC1a (Diochot et al., 2012). In the context of cancer, this channel has recently been described to be overexpressed in breast, melanoma, lung, and liver cancers (Jin et al., 2015; Bychkov et al., 2020; Yang et al., 2020; Wang et al., 2021; Sudarikova et al., 2022). The mambalgin-2 application to leukemia cells reduces their growth and induces cell cycle arrest (Bychkov et al., 2020). In glioma cells, the constant cation current required for cell growth and migration was shown to be mediated by ASIC1a (Rooj et al., 2012). Their treatment with mambalgin-2 induces cell cycle arrest and apoptosis. Acidification, which promotes cell proliferation, migration, and invasiveness, is facilitated by this channel in melanoma (Bychkov et al., 2021). Treatment with mambalgin-2 reduces this phenotype. The same conclusion has recently been drawn for lung adenocarcinoma (Sudarikova et al., 2022).

Kunitz-type serine protease inhibitors (KUN) are small proteins that contain a Kunitz domain. These domains are approximately 50–60 amino acid residues with alpha and beta-fold structures stabilized by three conserved disulfide bridges and inhibit the enzymatic activity of serine proteases (Munawar et al., 2018). KUNs have also been investigated for their promising anticancer activity. Vipegrin, extracted from *Daboia russelli* (Russell’s viper) venom, is cytotoxic against breast cancer cells while showing no significant effect on non-cancerous cells (Bhattacharya et al., 2023). This property suggests that vipegrin may follow a specific pathway for killing cancer cells, but unfortunately, it has not been identified to date. Another example is PIVL, a peptide derived from the venom of *Macrovipera lebetina,* which possesses an anti-tumor activity by primarily blocking integrin receptor function, resulting in reduced adhesion of cancer cells. This suggests that PIVL’s anticancer activity is not related to cell viability but affects cancer cell migration and invasion (Morjen et al., 2013).

Crotoxin, derived from the venom of *Crotalus durissus terrificus*, is a complex of two subunits, namely phospholipase A2 (crotactin) and a non-enzymatic subunit (crotapotin) that enhances the activity of the first subunit (Faure et al., 1993). This β-neurotoxin activates both autophagic and apoptotic pathways in leukemia, breast, and lung cancer cells (Yan et al., 2006; Yan et al., 2007; Han et al., 2014). Crotoxin has also been shown to enhance the efficacy of gefitinib in lung adenocarcinoma cells (Wang et al., 2012; Wang et al., 2014). Gefitinib is an inhibitor of the epidermal growth factor receptor (EGFR), which is used to treat lung cancer. One hypothesis explaining the synergistic effect is based on the observation that crotoxin modulates EGFR signaling (Donato et al., 1996). Recent studies show that crotoxin also exhibits cytotoxic effects against several cancer cell types, including esophageal, brain, cervical, and pancreatic cancer (Muller et al., 2018). Further evidence shows that crotoxin may have an anti-tumor effect on estrogen-positive (ER+) breast cancer by decreasing the phosphorylation of the ERK1/2 protein, with the antiproliferative effect then being related to the inhibition of the MAPK pathway (Almeida et al., 2021). Crotoxin treatment (10 μg/kg) did not induce any changes in body weight or biochemical parameters in mice (He et al., 2013; da Rocha et al., 2023). However, it was still effective in reducing tumor growth in transplanted esophageal and oral cancer mice. A phase 1 clinical trial was initiated to evaluate the pharmacokinetics of the toxin in patients with advanced breast cancer (Cura et al., 2002), and an open-label phase 1 clinical trial in patients with advanced cancer using intravenous administration was more recently initiated in 2018 and has shown promising results for the efficacy of the toxin in cancer treatment (see Table 3).

**TABLE 3:** Summary table of venom-derived molecules mentioned in clinical trials.

Tested molecule	Specie	Molecular mechanism	Target disease	Phase	Status	Trial number	References	Comment
Crotoxin	*Crotalus durissus terrificus*	Cytotoxic	Advanced solid tumors	Phase 1 part 3 (tolerability of intra-patient dose escalation with i.v. administration, confirmation of induction of drug tolerance and assessment of drug efficacy)	Not recruiting yet (08/2024)	NCT01481532	Cura et al. (2002)	
BLZ-100	*Leiurus quinquestriatus quinquestriatus*	Binding to cancer cells	Soft tissue sarcoma	Phase 1	Withdrawn	NCT02464332		
			Skin cancer	Phase 1 dose escalation/expansion study	Completed	NCT02097875	Yamada et al. (2021)	
			Glioma	Phase 1 dose escalation/expansion study	Completed	NCT02234297	Patil et al. (2019)	
			Pediatric subjects with primary central nervous system tumor	Phase 1 dose escalation/expansion study	Completed	NCT02462629		
			Solid tumors (surgical excision)	Phase 1 exploratory study of the safety	Completed	NCT02496065	Dintzis et al. (2019)	
			Pediatric subjects with primary central nervous system tumor	Phase 2/3	Completed	NCT03579602		
			Oral cavity squamous cell carcinoma and high-grade dysplasia	Phase 1/2	Recruiting	NCT05316688		
Xen2174	*Conus marmoreus*	Norepinephrine transporter inhibitor	Cancer patients with chronic pain	Phase 2b	Completed	EUCTR 2010-019109- 40BG	Okkerse et al. (2017)	Discontinuation due to dose-limiting toxicity
Contulakin-G	*Conus geographus*	Neurotensin receptor subunit of the NMDA receptor (hNTR1) agonist	Central neuropathic pain following spinal cord injury	Phase 1	Completed		Sang et al. (2016)	Discontinuation due to financial issues
Vc1.1 (ACV-1)	*Conus victoriae*	α9α10 nAChR antagonist	Healthy volunteers	Phase 1 multiple ascending dose study of the safety, tolerability and pharmacokinetics	Completed	ACTRN12605000408684		
			Diabetic peripheral neuropathic pain or post-herpetic neuralgia	Phase 2a study of the safety, tolerability, pharmacodynamics and pharmacokinetics	Stopped early	ACTRN12607000201471		Discontinuation due to lack of efficacy
ShK-186 (dalazatide)	*Stichodactyla helianthus*	K_V_1.3 channel antagonist	Healthy volunteers	Phase 1 multiple ascending dose study of the safety, tolerability and pharmacokinetics	Completed	NCT02446340		
			Plaque psoriasis	Phase 1 study of the safety, tolerability and pharmacodynamics	Completed	NCT02435342	Tarcha et al. (2017)	
SOR-C13	*Blarina brevicauda*	TRPV6 calcium channel inhibitor	Advanced solid tumors	Phase 1 dose escalation study	Completed	NCT01578564	Fu et al. (2017)	
			Advanced solid tumors	Phase 1	Completed	NCT03784677		

The Caspian cobra, *Naja naja oxiana*, is considered the most venomous species among the *Naja sp*. This cobra secretes a specific cytotoxin called oxineur (Sadat et al., 2023). Oxineur shows cytotoxic activity against colon cancer cells while not affecting normal cells. However, more extensive testing is required to evaluate the effects of its administration on animals.

Disintegrins are components of snake venoms that interact with integrins through the RGD domain (see Table 2). Because integrins are involved in angiogenesis and metastasis, integrin ligands are potentially potent anticancer reagents. For instance, obtustatin, a disintegrin inhibitor of the α1β1 integrin isolated from the venom of *Macrovipera lebetina obtusa* venom, inhibits melanoma growth in mice (Brown et al., 2008). The inhibition mechanisms are mainly due to obtustatin’s anti-angiogenic activity, which activates apoptosis in endothelial cells. Obtustatin also reduces tumor size in sarcoma-bearing mice, via angiogenesis inhibition (Ghazaryan et al., 2015; Ghazaryan et al., 2019). Contortrostatin, a disintegrin homodimer derived from the venom of *Agkistrodon contortrix contortrix*, is another potent integrin inhibitor, that is selective for β1, β3, and β5 integrins (Zhou et al., 2000a). Contortrostatin, although non-cytotoxic, inhibits the adhesion and invasion of breast cancer cells *in vitro* (Zhou et al., 2000b). This anti-invasive effect was attributed to the blockage of αvβ3, an integrin highly expressed in metastatic cells. Interestingly, migration and invasion are also reduced in prostate cancer. However, this effect cannot be attributed to αvβ3 inhibition as this prostate cancer cell line (PC-3) does not express this integrin but αvβ5 may be an alternative target (Lin et al., 2010). Furthermore, encapsulation of contortrostatin in liposomes prevents potential clinical side effects such as platelet binding and immunogenicity (Swenson et al., 2004). These findings are promising for the long-term use of the compound in clinical trials. According to a recent review published in 2020, the next step will be to submit an investigational new drug application to initiate a phase 1 clinical trial (Schonthal et al., 2020). In addition, Zhang and co-workers have recently developed a recombinant fusion protein known as IL-24-CN, a tumor suppressor protein (Zhang et al., 2021). Overexpression of IL-24 can inhibit cancer cell proliferation and induce apoptosis. The study successfully demonstrated the growth-suppressive and apoptosis-inducing effects of IL-24-CN on melanoma cells.

Vicrostatin is a disintegrin produced by recombination of the C-terminal tail of echistatin with contortrostatin (Minea et al., 2010). Despite its immunogenicity, this construct not only retains the native binding properties of contortrostatin but also shows an innovative binding to the integrin α5β1. Intravenous administration of vicrostatin in mice showed no side effects. As previously demonstrated for other disintegrins, vicrostatin can inhibit angiogenesis, thereby reducing both tumor vascular density and metastasis (Minea et al., 2010). In the context of glioma treatment, brachytherapy is an emerging method in which radioactive material is delivered to the tumor to minimize damage to healthy tissue. Radioiodinated vicrostatin (^131^I-VCN) has been developed to treat glioma, a tumor type expressing high levels of integrins. ^131^I-VCN has been successfully tested in glioma animal models and has been shown to prolong survival (Swenson et al., 2018). Moreover, Jadvar and colleagues have recently developed a ^64^Cu-labeled vicrostatin probe for PET imaging of tumor angiogenesis in prostate cancer, suggesting that venom components can be used as both diagnostic and therapeutic tools (Jadvar et al., 2019). Other disintegrins found in snake venom are also listed in Table 4.

**TABLE 4:** Other snake toxins with a potential interest in drug discovery.

Name	Species	Demonstrated effects	References
Leberagin-C (Leb-C)	*Macrovipera lebetina transmediterrannea*	- Reduction of adhesion, migration and invasion of breast cancer cells - Angiogenesis inhibition *in vitro* and *in vivo* - Reduction in tumor size *in vivo*	Limam et al. (2023)
r-viridistatin 2	*Crotalus viridis viridis*	- Inhibition of platelet aggregation - Inhibition of adhesion, migration and invasion of cancer cells - Inhibition of lung colonization of melanoma cells (B16F10) *in vivo*	Lucena et al. (2012)
Crotalicidin	*Crotalus durissus terrificus*	- Anti-tumoral effects - Anti-microbial effects - Anti-fungal activity - Antichagastic activity	Falcao et al. (2015) Perez-Peinado et al. (2020) Klaiss-Luna et al. (2023) Cavalcante et al. (2017) Aguiar et al. (2020) Bandeira et al. (2018)
Micrurotoxin-1 and 2	*Micrurus mipartitus*	GABA_A_ receptor activity modulation	Rosso et al. (2015)

Cathelicidins are a class of antimicrobial peptides found in insects, fish, amphibians, and mammals. They are effective against a wide range of bacteria, fungi, viruses, and protozoa (Wang et al., 2013). Interestingly, these peptides have also shown cytotoxic activity against cancer cells. More specifically, BF-30 is a cathelicidin-like polypeptide, extracted from *Bungarus fasciatus*. BF30 inhibits the proliferation of metastatic melanoma cells without affecting normal cells (Wang et al., 2013). *In vivo*, this compound effectively reduces cell proliferation and has low toxicity in mice. Furthermore, BF30 reduces angiogenesis by decreasing VEGF gene expression levels. BF-30 derivatives have been developed to improve the pharmacokinetic and pharmacodynamic parameters of BF-30 (Qi et al., 2020).

In the venom of *Crotalus durissus terrificus,* crotamine is a β-defensin that possesses cell-penetrating properties by efficiently translocating into cells (Pereira et al., 2011). Crotamine exhibits targeted cytotoxicity against melanoma cell lines, with a specificity of 5 times higher than normal cells. Interestingly, no toxicity was observed in treated animals. The precise mechanisms underlying the cytotoxic effects of crotamine are not well understood. One hypothesis is that crotamine is endocytosed and transported to the lysosome, resulting in an increase in lysosomal content and the leakage of content into the cytosol. Furthermore, lysosomes have been shown to trigger intracellular Ca^2+^ transients and affect mitochondrial membrane potential (Nascimento et al., 2012). In addition, crotamine has been found to accumulate in tumor cells, suggesting that it could act as a diagnostic tool like vicrostatin (Kerkis et al., 2014). To facilitate the advancement of crotamine in clinical trials, an oral administration of the molecule was assessed in animals (Campeiro et al., 2018). Small changes in glucose clearance, total cholesterol, triglyceride, and lipoprotein levels were measured but were considered harmless. No other adverse toxic effects were observed. Synthetic crotamine has since been produced with similar properties to the native peptide, allowing for improved analogs with fewer potential side effects and better properties (de Carvalho Porta et al., 2020). This provides an opportunity for further research into developing new applications for these analogs.

#### 2.3.3 Neurological disorders

**FIGURE 4:**
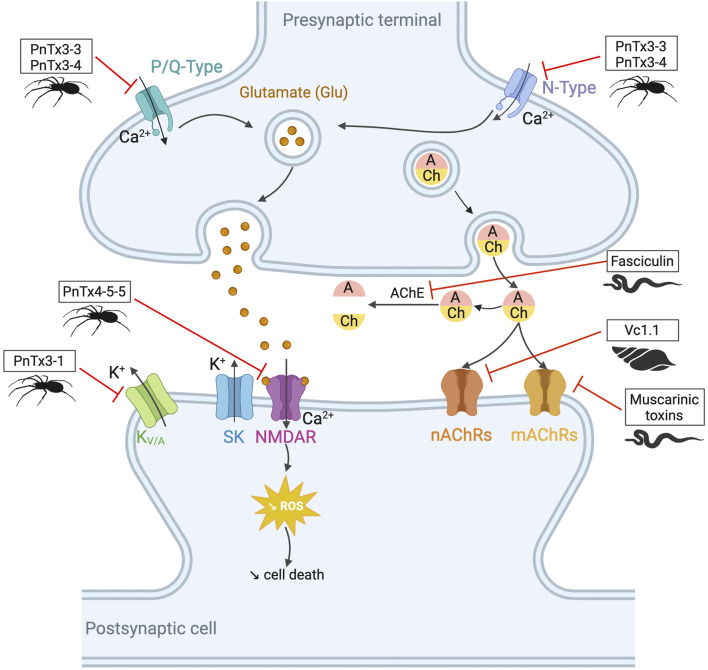
Potential toxin inhibitors involved in neurological disorders, such as Alzheimer’s disease, Parkinson’s disease, and chronic pain. Vc1.1 has antagonistic activity on neuronal nAChRs involved in neuropathic pain (Sandall et al., 2003; Bordon et al., 2020). Inhibition of AChE by fasciculin helps to counteract the acetylcholine deficiency seen in disorders such as Alzheimer’s disease. Blocking K_V/A_ channels with PnTx3-1 reduces memory deficits (Gomes et al., 2013). PnTx4-5-5 has a neuroprotective effect by blocking the NMDA receptors by reducing glutamate release (Silva et al., 2016). N- and P/Q-type channels also release glutamate by controlling the calcium flux. PnTx3-3 and PnTx3-4 have inhibitory activity on these two channels (Vieira et al., 2005; Dalmolin et al., 2011; Souza et al., 2012; Pedron et al., 2021). The reduction of glutamate prevents ROS formation. Muscarinic toxins can regulate mAChRs when they are dysfunctional. Adapted from “NMDAR-dependent long-term depression (LTD),” Created with BioRender.com (2023).

Snake venom contains other toxins that may have potential in the treatment of neurodegenerative diseases, such as Alzheimer’s and Parkinson’s (see Figure 4). For instance, interesting bioactivity in the context of Alzheimer’s disease comes from fasciculin, a 61-residue 3FTx isolated from *Dendroaspis angusticeps* (Waqar and Batool, 2015). By inhibiting the acetylcholinesterase (AChE), fasciculin restores normal levels of acetylcholine (Harel et al., 1995). Since a reduction in this neurotransmitter leads to cognitive impairment, particularly the memory loss associated with Alzheimer’s disease (Winblad and Jelic, 2004), the effect of fasciculin may be beneficial. In parallel, RVV-V, a peptide discovered in the venom of *Daboia russelli russelli* is a procoagulant enzyme activator of factor V that destabilizes β-amyloid (Aβ) aggregates. Alzheimer’s disease is characterized by insoluble plaques composed of Aβ peptide fibrils. Destabilizing these aggregates would help prevent amyloidosis (Bhattacharjee and Bhattacharyya, 2013).

Ionotropic γ-aminobutyric acid type A (GABA_A_) receptors are massively present in the central nervous system. They modulate Cl^−^ conductance across the cell membrane and thus shape synaptic transmission (Sieghart, 2006). These receptors have been implicated in many diseases including epilepsy, schizophrenia, and chronic pain. Some snake toxins (α-bungarotoxin and α-cobratoxin) show activity for GABA_A_ receptors, but unfortunately also act on nAChRs avoiding any easy use of these toxins for medical purposes.

The venom of the Eastern green mamba, *Dendroaspis angusticeps*, but also the black mamba *Dendroaspis polylepis polylepis* (black mamba), contains muscarinic toxins that selectively target muscarinic acetylcholine receptors (mAChRs; M1 – M5). These toxins exhibit high affinity, selectivity, and low reversibility for their receptors (for a table with the inhibition constants of each toxin for each channel subtype, see the review by Servent et al. (2011)). M1, M4, and M5 are mainly found in the central nervous system, whereas M2 and M3 are found in the central and peripheral nervous systems. Muscarinic toxins offer the possibility to regulate dysfunctional receptors and thus provide solutions for neurological diseases as well as diseases related to the peripheral system, such as chronic obstructive pulmonary disease, incontinence, overactive bladder, etc*.* (Servent et al., 2011). The Eastern green mamba venom is a rich source of drug candidates as another toxin, called mambaquaretin-1 (MQ-1) shows high affinity and selectivity for the vasopressin type 2 receptor (V2R), with a Ki = 2.81 nM (Ciolek et al., 2017). Interestingly, MQ-1 does not interact with the other subtypes of the vasopressin receptors (V1a, V1b) and with the oxytocin receptor OT (Ki > 1 mM), making it a true molecular tool for the specific study of the V2R. From a therapeutic point of view, this Kunitz-type venom protein has the potential to treat polycystic kidney disease (PKDs), a genetic disorder characterized by the formation of numerous cysts in the kidneys, leading to end-stage renal failure. Selective inhibition of the V2 receptor reduces cAMP levels. This molecule stimulates chloride-induced cell proliferation and fluid secretion into the cyst lumen in polycystic kidneys. Since the discovery of MQ-1, eight other mambaquaretin-like toxins have been discovered in mamba’s venoms. All of them are antagonists of V2R, interacting with the receptor with nanomolar affinity (Droctove et al., 2022).

## 3 Harnessing the power of arthropod venom for next-generation therapies

Arthropods are the largest group of animals on Earth, comprising approximately 80% of the 1.5 million described animal species (according to the IUCN Red List in 2023). This phylum includes insects, arachnids, crustaceans, and myriapods, such as bees, scorpions, and spiders (Soltan-Alinejad et al., 2022).

### 3.1 Scorpion venoms, champions ion channel targeting

Scorpions have evolved over 400 million years to produce powerful toxins that affect various targets, especially localized in the nervous system (Estrada-Gomez et al., 2017). Scorpion venoms include peptides, enzymes, and non-protein compounds, such as salts, free amino acids, lipids, nucleotides, and neurotransmitters (Almaaytah and Albalas, 2014). Peptides are divided into two main classes according to their structural and functional properties: disulfide-bridged peptides (DBPs), and non-disulfide-bridged peptides (NDBPs). Five families comprise the DBPs, sodium channel toxins (NaTx), potassium channel toxins (KTx), chloride channel toxins (ClTx), calcium channel toxins (CaTx), and transient receptor potential channel toxins (TRPTx), all described in Table 5. Among the NDBPs, short antimicrobial peptides (AMPs) and bradykinin potentiating peptides (BPPs) are commonly found (Hmed et al., 2013). As seen in the case of the snakes, scorpions produce L-amino acid oxidases (LAAOs), serine proteases, hyaluronidases, metalloproteinases, nucleotidases, and phospholipases A2 (Soltan-Alinejad et al., 2022). Although NDBPs do not have specific ion channel targets, they are increasingly studied for their potential as antimicrobial, antiviral, and anticancer agents (Almaaytah and Albalas, 2014).

**TABLE 5:** DBPs scorpion families.

Toxin family names	Information	Examples	References
NaTx	Major component of scorpion venom 13–56 residues 3–4 disulfide bridges Two subgroups: α-NaTx (bind to receptor site 3) and β-NaTx (bind to the receptor site 4)	α-toxin OD1 (*Odontobuthus doriae*): 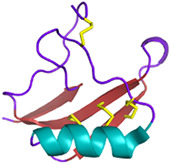 PDB 4HHF	Xia et al. (2023)
KTx	23–64 residues 2–4 disulfide bridges Six subgroups depending on their sequence and structural folds: α-KTx, β-KTx, γ-KTx, κ-KTx, δ-KTx, λ-KTx, and ε-KTx	Charybdotoxin (*Leiurus quinquestriatus*): 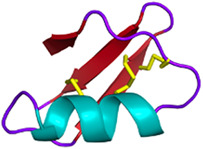 PDB 2CRD	
CaTx	33–36 residues 2–3 disulfide bridges Two subgroups with an ICK motif or a disulfide-directed β-hairpin (DDH) fold	ICK motif - Imperatoxin (*Pandinus imperator*): 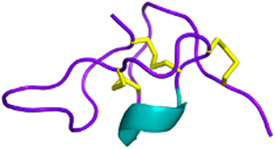 PDB 1IE6	
		DDH motif - U1-liotoxin-Lw1a (*Liocheles australasiae*): 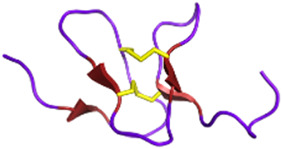 PDB 2KYJ	
ClTx	35–38 residues 4 disulfide bridges	Chlorotoxin (*Leiurus quinquestriatus*): 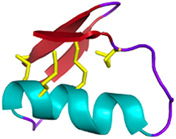 PDB 1CHL	
TRPTx	2–3 disulfide bridges Recently discovered	Wasabi receptor toxin (*Urodacus manicatus*): 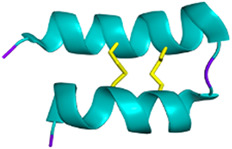 PDB 6OFA	

Overall, scorpion DBP toxins primarily target ion channels (ICs). Because ICs modulate essential functions in the body, their dysfunction can lead to the development of neurological disorders such as chronic pain, depression, autoimmune diseases, epilepsy, and cancer, as well as metabolic diseases such as diabetes. These dysfunctions, known as channelopathies, can be caused by the deregulation of channel opening/closing, changes in current amplitude, or problems regulating protein activity (Mendes et al., 2023). Neurotoxins of arthropods targeting these channels with remarkable specificity and potency constitute true molecular scalpels for studying IC distributions, functions, structures, and real candidates for tomorrow’s drugs. This review section is divided into five parts corresponding to the sodium, potassium, chloride, and calcium channels targeted, as well as other potential activities of scorpion venom.

#### 3.1.1 Sodium channels toxins (NaTxs)

Voltage-gated sodium channels (Na_v_) play an essential role in pain transmission, especially with Na_v_1.7, Na_v_1.8, and Na_v_1.9 subtypes, the most expressed in sensory neurons. Scorpion-derived peptides exert analgesic effects by regulating various Na_v_ channels, especially Na_v_1.1, Na_v_1.6, Na_v_1.7, Na_v_1.8, and Na_v_1.9 (Cummins et al., 2007; Eagles et al., 2022). Many NaTxs possess analgesic potency. Among them, BmK AS, BmK AS1, and BmK IT2 act on Na_v_1.8, Na_v_1.9, and Na_v_1.3 by reducing the peak Na^+^ conductance in dorsal root ganglia (DRG) neurons. Others like BmKM9, BmK AGAP, and AGP-SYPU1 inhibit the inactivation of the activated Na_v_1.4, Na_v_1.5, and Na_v_1.7. All these peptides are derived from *Buthus martensii* Karsch. BmK AGAP alleviates inflammatory pain by inhibiting the expression of peripheral and spinal mitogen-activated protein kinases and induces long-lasting analgesia by blocking TRPV1 currents when injected with lidocaine. It is considered a promising analgesic due to its multitarget capabilities (Kampo et al., 2021). Despite the discovery of numerous potent and selective Na_v_ channel inhibitors, which are pharmacologically interesting, very few of these inhibitors have resulted in effective pain relief in preclinical models or human clinical trials (Eagles et al., 2022).

#### 3.1.2 Potassium channels toxins (KTxs)

Potassium channels are divided into four groups according to their activation mode and the number of transmembrane segments (TM). Inwardly rectifying K^+^ (KIR) channels have 2 TM and two pore domains, whereas potassium channels (K2P) consist of 4 TM and two pores, K_Ca_ are calcium-activated potassium channels with 6 or 7 TM, and K_V_ are voltage-gated potassium channels with 6 TM (Wulff et al., 2009). K_V_ channels have been implicated in several diseases including cancer, autoimmune, neurological, and cardiovascular diseases.

In 1984, patch-clamp studies highlighted the role of the voltage-gated K_V_ channels in the activation of thymus-derived lymphocytes (T cells). Therefore, ion channels are involved in the immune response (Matteson and Deutsch, 1984). K_V_1.3 (KCNA3) and calcium-activated K_Ca_3.1 channels are primarily responsible for K^+^ efflux and are important therapeutic targets in various autoimmune diseases, such as multiple sclerosis, rheumatoid arthritis, and type-1 diabetes (Chandy and Norton, 2017). Charydbotoxin (ChTX), identified from the venom of *Leiurus quinquestriatus*, is a blocker of K_V_ channels (Kd = 3 nM) but also of intermediate conductance calcium-activated channels (IK_Ca_1) (Kd = 5 nM). Other inhibitors of the K_V_1.3 channel are margatoxin (MgTX), from *Centruroides margaritatus*, and HsTX1 from *Heterometrus spinnifer* venom. They are both potent blockers in the picomolar range of K_V_1.3. HsTX1 is a potentially attractive candidate for the treatment of K_V_1.3-related diseases due to its selectivity (IC_50_(K_V_1.3) = 29 ± 3 pM; IC_50_(K_V_1.1) = 11,330 ± 1,329 pM). To further improve selectivity, analogs of this toxin (HsTX1[R14A] and HsTX1[R14Abu]) have been developed. The arginine at position 14 is replaced by a neutral residue to prevent ionic interaction with K_V_1.1. This toxin binds to the E353 amino acid of this potassium channel but does not bind to K_V_1.3. Thus, the affinity for K_V_1.1 is reduced without affecting the affinity for K_V_1.3. The selectivity of HsTX1[R14A] is then more than 2,000-fold for K_V_1.3 over K_V_1.1 (Rashid et al., 2014). Other synthetic analogs of scorpion toxins show potent activity against K_V_1.3. Among them, OSK-1[E16K, K20D] has a five-fold higher IC_50_ than the native peptide OSK-1 (α-KTx3.7) from *Orthochirus scrobiculosus*: 3 pM versus 14 pM, respectively (Mouhat et al., 2005). Other peptides are listed in Table 6.

**TABLE 6:** Other scorpion toxins with a potential interest in drug discovery.

Name	Species	Demonstrated effects	References
ADWX-1	Modification of BmKTX (from *Buthus martensi*)	K_V_1.3 inhibition	Han et al. (2008)
Maurotoxin	*Scorpio maurus*	IK_Ca_1 channel inhibition	Kharrat et al. (1996) Castle et al. (2003)
Hemicalcin	*Hemiscorpius lepturus*	Stimulation of ryanodine binding to ryanodin receptor type 1 (RyR1)	Shahbazzadeh et al. (2007)
Opicalcin 1 and 2	*Opistophthalmus carinatus*	Stimulation of ryanodine binding to RyR	Zhu et al. (2003)
Hadrucalcin	*Hadrurus gertschi*	Stimulation of ryanodine binding to RyR1 and 2	Schwartz et al. (2009)
Urocalcin	*Urodacus yaschenkoi*	Stimulation of ryanodine binding to RyR	Luna-Ramirez et al. (2013)
Vejocalcin	*Vaejovis mexicanus*	Stimulation of ryanodine binding to RyR	Xiao et al. (2016)

**FIGURE 5:**
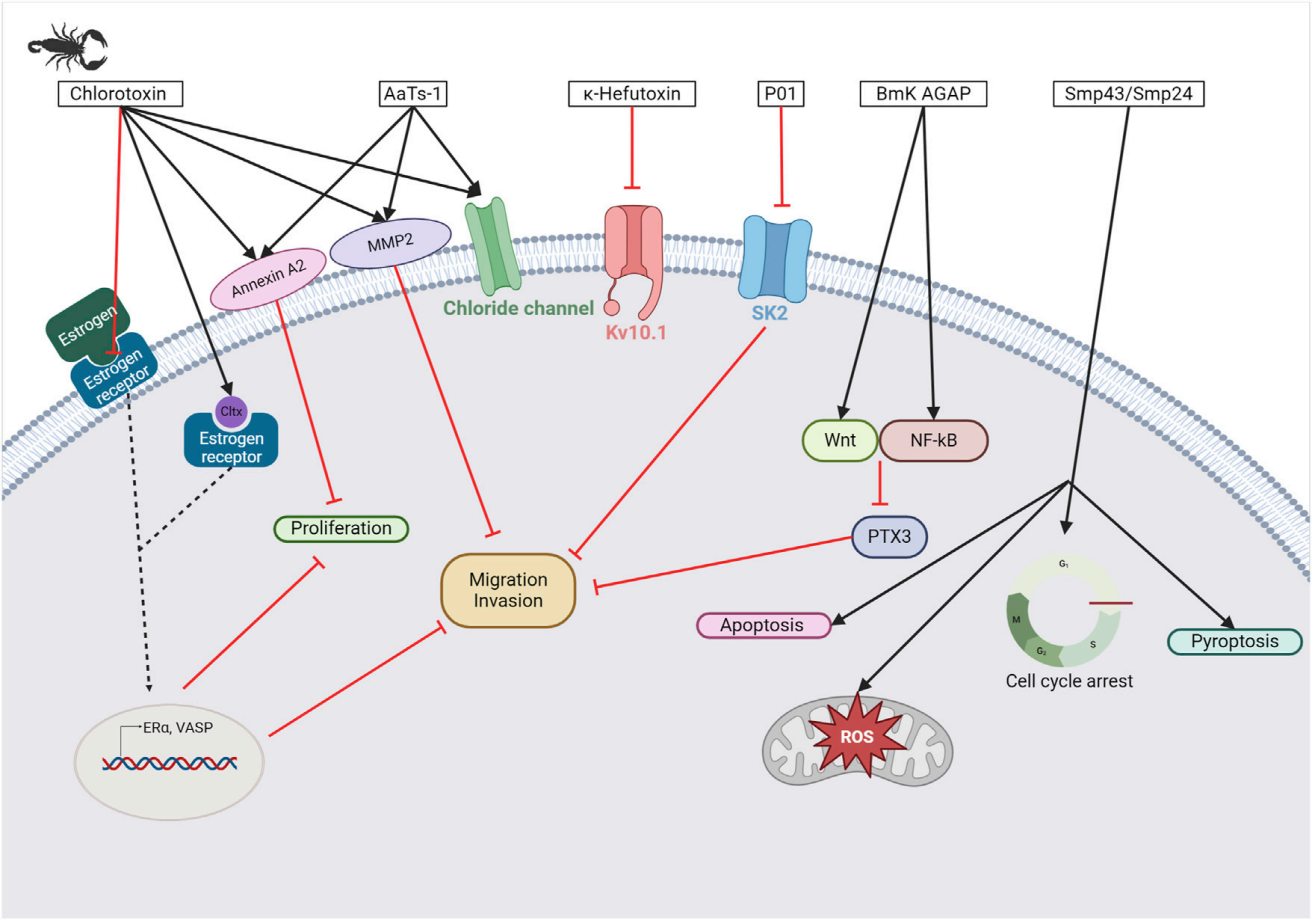
Scorpion toxins as potential anticancer therapy. Chlorotoxin has four cellular targets: estrogen receptor (ERα), annexin A2, MMP2, and chloride channel. Overall, the binding of chlorotoxin to these targets leads to inhibiting cell proliferation and/or reducing cancer cell migration and invasion (Boltman et al., 2023). Binding to chloride channels can also help to visualize tumor sites in brain tumors. Sharing 80% of homology with chlorotoxin, AaTs-1 binds annexin A2, MMP2, and chloride channels (Aissaoui-Zid et al., 2021). κ-hefutoxin-1 is a K_V_10.1 inhibitor, a potassium channel known to be overexpressed in several cancer types (Pardo et al., 1999; Moreels et al., 2017). P01 is a potent SK2 channel inhibitor that leads to the inhibition of cancer cell migration and invasion (Mlayah-Bellalouna et al., 2023). BmK AGAP has been shown to reduce cancer cell migration and invasion by the downregulation of PTX3 via NF-κB and Wnt/β-catenin (Kampo et al., 2019). Finally, Smp43 and Smp24 are two antimicrobial peptides that can trigger apoptosis, pyroptosis, an accumulation of reactive oxygen species, or a cell cycle arrest in cancer cells (Chai et al., 2021; Elrayess et al., 2021). Created with BioRender.com (2024).

Scorpion venom also contains several peptides with anticancer activity (see Figure 5). For instance, κ-hefutoxin-1, a peptide isolated from the venom of *Heterometrus fulvipes*, is a potassium channel inhibitor. Specifically, it can inhibit the oncogenic K_V_10.1 channel, which is overexpressed in several types of cancer (Pardo et al., 1999; Moreels et al., 2017). However, the effects of this peptide on cancer cells remain to be determined. Interestingly, P01-toxin, extracted and purified from the venom of *Androctonus australis* is a potent inhibitor of the SK2 potassium channel (Mlayah-Bellalouna et al., 2023). While the peptide was shown to reduce cell viability, adhesion, and migration in glioma cells, no such effects were observed in breast and colon cancer cells. These results suggest that SK2 channels are involved in the formation of glioma tumors. Another peptide toxin derived from the same species, AaTs-1, shares more than 80% homology with chlorotoxin (Aissaoui-Zid et al., 2021). Like chlorotoxin, AaTs-1 binds to chloride channels, MMP-2, and annexin 2, leading to a reduction in glioma cell proliferation and migration. In terms of anticancer activity, the effects of *Buthus martensii Karsh* antitumor analgesic peptide (BmK AGAP) on breast cancer cells have been studied, revealing its ability to inhibit cancer cell stemness, epithelial-mesenchymal transition, migration, and invasion (Kampo et al., 2019). The mechanisms underlying these effects have been investigated, and it has been found that the downregulation of PTX3 via NF-κB and Wnt/β-catenin signaling is critical.

#### 3.1.3 Chloride channels toxins (ClTxs)

ClTxs are divided into two subgroups. The vast majority have an inhibitory cystine knot (ICK) motif, characterized by two disulfide bonds pierced by a third to form a pseudoknot. The second motif, the disulfide-directed hairpin (DDH), would result from a simplification of the ICK motif to only two bonds (Smith et al., 2013). The best-known toxin targeting chloride channels is chlorotoxin, isolated from the venom of *Leiurus quinquestriatus*. Chlorotoxin can also bind to matrix metalloproteinase-2, annexin A2, estrogen receptor α, and neuropilin-1 receptor. This peptide has been extensively studied in the context of glioblastoma and neuroblastoma where those proteins are all involved in cell migration and invasion, as recently reviewed by Boltman and colleagues (Boltman et al., 2023). In addition, chlorotoxin has a wide range of applications, including tumor imaging and combination with other therapeutics or molecules as this peptide can cross the blood-brain barrier (Veiseh et al., 2007; Formicola et al., 2019; Vannini et al., 2021; Dardevet et al., 2022). Numerous clinical trials are underway to establish the safety and pharmacokinetic properties of BLZ-100, a chlorotoxin-based imaging agent containing indocyanine green as a fluorophore (Patil et al., 2019) (see Table 3). The efficacy of chlorotoxin in the treatment of other cancers has also been investigated. Efficacy against cervical cancer cells is significantly improved when coupled with a platinum complex (Graf et al., 2012). In breast cancer, chlorotoxin has the potential to inhibit cell proliferation, migration, and invasion by either directly binding to the estrogen receptor (ER) or by preventing estrogen binding to its receptor. This thereby inhibits the ER signaling pathway (Wang et al., 2019).

#### 3.1.4 Calcium channels toxins (CaTxs)

Because calcium channels are involved in pain pathways, Parkinson’s disease, epilepsy, seizures, migraine, and ataxia, they are promising and interesting targets (Zamponi, 2016). As scorpion venoms are a rich source of toxins that act on Ca^2+^ channels, they may have therapeutic potential. Such scorpion toxins include calcins, a family of cell-penetrating peptides composed of 33 residues (35 for hadrucalcin) and three disulfide bridges. They have an ICK motif and potently target Ryanodine receptors (RyRs), intracellular ion channels that regulate the Ca^2+^ release from the endoplasmic and sarcoplasmic reticulum, thereby triggering myocardial contraction (Vargas-Jaimes et al., 2017). Imperacalcin (formerly imperatoxin A), identified from the venom of *Pandinus imperator*, is the first member of the calcium-targeting toxins to bind to RyR1 with nanomolar affinities (Valdivia et al., 1992). Subsequently, maurocalcin, from the venom of *Scorpio maurus palmatus*, was isolated based on sequence similarity to imperacalcin (82% sequence identity). Both increase skeletal RyR (RyR1) activity but also have a nanomolar affinity for cardiac RyR (RyR2) (De Waard et al., 2020). Other toxins of interest are listed in Table 6.

CPP-Ts, isolated from *Tityus serrulatus* venom, is a cell-penetrating peptide, that crosses both cellular and nuclear membranes. This peptide increases the contractile frequency of cardiomyocytes by activating the inositol 1,4,5-trisphosphate receptor (InsP3R), a ligand-gated Ca^2+^ release channel. This activation leads to a transient change in intracellular calcium levels. CPP-Ts can be internalized by cancer cells and not by normal cell lines, making it a potential intranuclear delivery tool to target cancer cells (Oliveira-Mendes et al., 2018).

#### 3.1.5 Other targets

Scorpion venom also contains antimicrobial peptides (AMPs), which belong to the group of non-disulfide-bridged peptides (NDBPs) (Almaaytah and Albalas, 2014). Their role in venom and their molecular target remains to be elucidated. However, the antimicrobial peptides Smp43 and Smp24 from the Egyptian scorpion *Scorpio maurus palmatus* were studied in different cancer cell types including hepatocellular, non-small cell lung, and leukemia cancer cell lines. Smp43 exhibits antitumor properties in hepatocellular carcinoma by inducing apoptosis, autophagy, necrosis, and arresting cell cycle progression (Chai et al., 2021). In addition, both peptides stimulate pyroptosis, a regulated cell death mechanism that recruits the inflammasome, which subsequently activates caspases (Elrayess et al., 2021). These two peptides also induce a loss of mitochondrial membrane potential, leading to the accumulation of reactive oxygen species in lung and hepatocellular cancer cells (Guo et al., 2022; Nguyen T. et al., 2022; Deng et al., 2023). Interestingly, Smp43 only has minor effects on a lung fibroblast cell line, MRC-5 (Deng et al., 2023).

Scorpion peptides have also been investigated for the treatment of malaria. This disease, caused by *Plasmodium falciparum* infection, occurs in more than one hundred countries and can be fatal, especially in children (Murray et al., 2012). Scorpine, isolated from *Pandinus imperator*, shows activity in the ookinete and gamete stages of the development of the parasite *Plasmodium berghei*. Since the developmental stages of the two parasites are the same, scorpine could represent a promising model for malaria treatment (Conde et al., 2000). Lastly, some peptides with antimicrobial activities are also important antimalarial candidates, such as meucin-24, meucin-25, and hadrurin (Ortiz et al., 2015).

### 3.2 Spider venoms, on the way to a new drug for heart attack and beyond

Spiders, like scorpions, have evolved over more than 400 million years. Although about 50,000 species have been described so far, the diversity is estimated to be more than 100,000 (Agnarsson et al., 2013; World Spider Catalog, 2023). Spider venoms consist of proteins, peptides, nucleotides, and small molecular weight organic molecules, such as organic acids, nucleotides, amino acids, amines … , and salts (Smith et al., 2015). Peptides without disulfide bonds, often antimicrobial peptides, are represented in these venoms but their major components are disulfide-rich peptides, which possess an ICK motif, in most cases, or a DDH fold, a Kunitz motif, a colipase-like fold or a helical arthropod-neuropeptide-derived (HAND) motif (for more information on these motifs, see the review by Langenegger et al. (2019). Ion channels are the main targets of spider toxins and, more precisely voltage-dependent sodium and calcium channels (Na_V_ and Ca_V_) representing up to 75% of the total number of receptors targeted. Various enzymes, such as hyaluronidase, phospholipase A2, and proteases, are the primary protein families found in spider venom. Therefore, they contain a valuable collection of biologically active peptides of interest for drug discovery (King and Hardy, 2013). Unfortunately, spider venom has long remained unexplored. For a long time, venom research focused only on certain species of venomous animals, primarily snakes (King, 2011). “The limited availability of venom from species that produce small amounts or are rare was due to unsuitable techniques uses. The development of omics techniques like transcriptomics and proteomics has opened up opportunities for the study of these long-neglected species” (Holford et al., 2018).

#### 3.2.1 Regulation of insulin secretion

**FIGURE 6:**
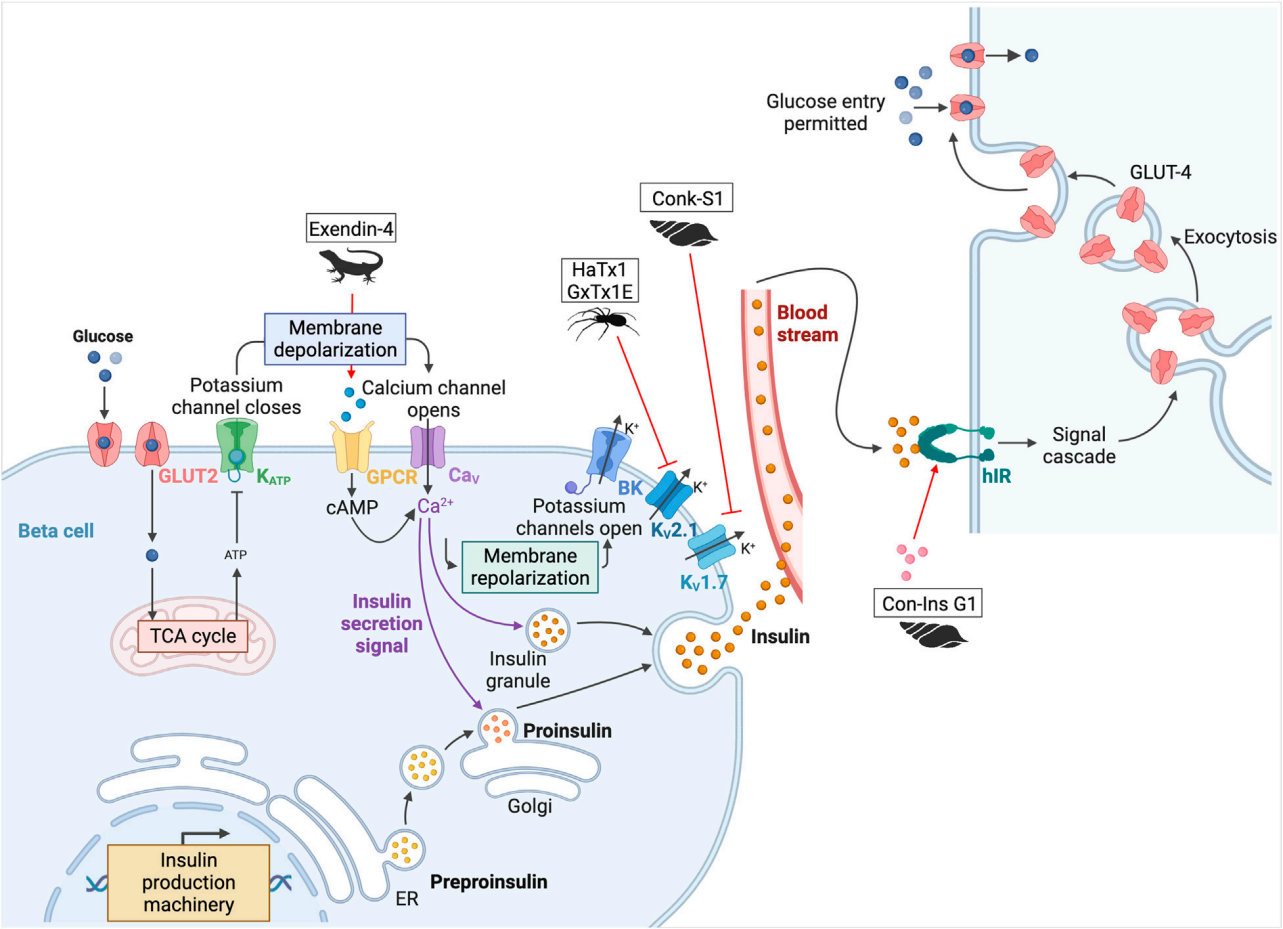
Potential toxins involved in the insulin pathway. Glucose is transported into pancreatic beta cells by facilitated diffusion through GLUT2 glucose transporters. Once inside the cell, glucose is converted to ATP by glycolysis and oxidative phosphorylation. When the ATP/ADP ratio is high, K^+^ channels are inhibited, leading to cell membrane depolarization. Closure of the K_ATP_ channels leads to the depolarization of the cell, which opens the Ca_V_ channels and triggers the action potential that allows insulin granules to be released from the pancreas. This is followed by repolarization of the cell with activation of BK, K_V_2.1, and K_V_1.7. Once produced, insulin is delivered to target tissues, such as the liver, adipocytes, muscle, and brain. Insulin binds to hIR, initiating a phosphorylation cascade that ultimately leads to glucose uptake and storage in glycogen, thereby lowering blood glucose levels. HaTx1 and GxTx1E are two spider peptides, and Conk-S1 is a cone snail peptide that inhibits K_V_2.1 and K_V_1.7 respectively (Herrington et al., 2005; Herrington, 2007; Finol-Urdaneta et al., 2012). Inactivation of these channels leads to an increase in glucose-stimulated insulin secretion. Another cone snail toxin, Con-Ins G1 is an insulin-like peptide that can activate hIR (Xiong et al., 2020). Finally, the most famous one is exenatide-4, from the Gila monster, which led to the development of the drug exenatide (Byetta^©^) (Nadkarni et al., 2014). This peptide binds to the incretin hormone GLP-1 receptor. This GPCR stimulates adenylyl cyclase activity and cAMP accumulation, leading to insulin secretion. Adapted from “Insulin production pathway” and “Insulin pathway,” created with BioRender.com (2024).

As mentioned above, arthropod venom contains toxins with activities on ion channels. These channels are involved in physiological mechanisms, including the regulation of insulin secretion by glucose. They allow membrane depolarization and trigger an action potential that induces the release of insulin granules. The channels involved in this process are ATP-sensitive potassium (K_ATP_) channels. Their closure leads to the depolarization of the cell, which opens voltage-dependent calcium (Ca_V_) channels, triggering the action potential that allows insulin granules to be released from the pancreas (Ashcroft and Rorsman, 1989). This is followed by repolarization of the cell with activation of large conductance calcium-activated K^+^ (BK) and voltage-gated potassium (K_V_2.1 and K_V_1.7) channels (Herrington, 2007). κ-theraphotoxin-Gr1a (hanatoxin-1, HaTx1), a toxin with inhibitory activity on these K_V_2.1 channels, was isolated from the venom of the Chilean pink tarantula, *Grammostola rosea* (Swartz and MacKinnon, 1995). By blocking them, the HaTx1 increases glucose-stimulated insulin secretion (see Figure 6) (Herrington et al., 2005). Unfortunately, this peptide, as well as guangxitoxin-1 (GxTx1E, κ-theraphotoxin-Pg1a), isolated from the venom of the Chinese tiger tarantula (*Chilobrachys guangxiensis*), also shows an affinity for other various channels, such as K_V_4.2 and Ca_V_2.1 and K_V_2.2 and 4.3 respectively. This lack of selectivity prevents its use as a treatment (Herrington, 2007).

#### 3.2.2 Chronic pain and neurological disorders

The ion channel activity of spider venom peptides may lead to potential treatments for chronic pain (see Figure 4). Acid-sensing ion channels (ASICs), transient receptor potential (TRP), and Na_V_ and Ca_V_ channels are involved in the transduction of stimuli into depolarization of the cell membrane and are therefore important in the development of analgesics (King and Vetter, 2014). Among the ion channels, voltage-gated calcium channels are the main target of spider toxins. For instance, the venom of *Phoneutria nigriventer,* one of the most studied with not less than 41 neurotoxins identified, is a rich source of potential analgesic drugs due to its activity on Ca_V_ channels (Peigneur et al., 2018). ω-ctenitoxin-Pn2a (also known as PnTx3-3) and ω-ctenitoxin-Pn4a (PnTx3-6 or Phα1β), two toxins identified from this venom, both block Ca_V_2.1, Ca_V_2.2, and Ca_V_2.3 channels, as well as Ca_V_1 and Ca_V_1.2. Despite the apparent lack of selectivity, the peptides show analgesic activity in mouse models without side effects (Vieira et al., 2005; Dalmolin et al., 2011). As PnTx3-3 reduces pain and depressive symptoms, it is a credible drug candidate for fibromyalgia (Pedron et al., 2021). In addition to opioid treatment, PnTx3-6 potentiates the analgesic effect of morphine and reduces the adverse effects of regular morphine use, such as tolerance, constipation, and withdrawal symptoms (de Souza et al., 2011). *Phoneutria nigriventer* venom is also being studied for Huntington’s disease, a fatal neurodegenerative disorder, as Joviano-Santos and colleagues recently demonstrated the neuroprotective effect of PnTx3-6 (Joviano-Santos et al., 2021). Indeed, neuronal survival was improved, and the release of L-glutamate was reduced in mice treated with the toxin. Huntington’s disease is associated with the formation of insoluble aggregates and glutamatergic excitotoxicity associated with progressive neuronal death. This led to an improvement in behavioral and morphological parameters related to motor tests (Joviano-Santos et al., 2021). PnTx3-6 may have important potential in various diseases. Compared to current drugs (morphine and ziconotide), the spider toxin is more effective and has fewer side effects (Rigo et al., 2013). The inconvenience is the limitation of administration, as it is unlikely to be available orally (Tonello et al., 2014).

Other toxins inhibiting Ca_V_2.2 (N-type) channels are of primary interest because of their involvement in pain pathways (for review see Sousa et al., 2013). In addition to chronic pain, Ca_V_2.1 (or P/Q type) channels have been implicated in many neurological disorders including migraine, Alzheimer’s disease, and epilepsy (Nimmrich and Gross, 2012; Inan et al., 2024). ω-Agatoxin-Aa4a (ω-agatoxin IVA) and ω-agatoxin-Aa4b (IVB), from the venom of the American funnel-web spider *Agelenopsis aperta*, are the most selective blockers of this calcium channel subtype, with an IC_50_ of about 2 and 3 nM, respectively. The remaining problem for this type of peptide is the poor permeability of the blood-brain barrier (Smith et al., 2015).

Na_V_1.7 – Na_V_1.9 voltage-gated sodium channels are expressed in nociceptive neurons and therefore play a critical role in pain signaling. Na_V_1.7 is by far the most important target for analgesic development (Alexandrou et al., 2016). All spider toxins identified to bind to this channel come from theraphosid spiders (tarantulas) and share the ICK motif. These include huwentoxin-IV (*Haplopelma schmidti*), GpTX-1 (*Grammostola portei*), ceratotoxin-1 (*Ceratogyrus cornuatus*), Pn3a (*Phamphobeteus nigricolor*), β-theraphotoxin-Tp2a/ProTx-II (*Thrixopelma pruriens*), and β-theraphotoxin-Cj2a/JzTX-V (*Chilobrachys jingzhao*). ProTx-II is the most potent Na_V_1.7 blocker (IC_50_ = 0.3 nM) of the six currently known, but none is sufficiently selective to be developed as a therapeutic drug. The recent review from Neff and his co-workers describes the peptide engineering of each toxin to achieve better selectivity and highlights some interesting analogs (Neff and Wickenden, 2021). JNJ-63955918, derived from the ProTx-II, increases the selectivity for Na_V_1.7 from 100- to 1000-fold compared to other Na_V_ channels, but unfortunately, the affinity is altered by ∼10-fold (Flinspach et al., 2017). AM-6120, derived from JzTX-V, was designed as a potent and selective peptide with >750-fold potency against Na_V_1.5, 1.6, and 1.8. Similarly, ProTx-II analogs optimized for the ability to cross the blood-nerve barrier *in vivo* have recently been successfully developed (Adams et al., 2022; Nguyen P. T. et al., 2022).

Spider toxins targeting Na_V_ channels are not only interesting for pain treatment. Na_V_1.1 channels are involved in Dravet syndrome, a form of infantile epilepsy with ataxia and loss of motor skills. Hm1a, identified from *Heteroscodra maculata* venom, selectively inhibits these Na_V_1.1 channels and constitutes a promising candidate for treating the disease as its administration improved seizure inhibition and reduced the number of seizures per day in mouse models (Richards et al., 2018).

Spider venom has also been extensively studied in stroke. During cerebral ischemia, which occurs in most strokes (>80%), oxygen is depleted and the brain switches from oxidative phosphorylation to anaerobic glycolysis (Duggan et al., 2021). The pH drops from ∼7.3 to 6.0–6.5 and even below 6.0 in severe ischemia. This low pH activates the acid-sensing ion channels 1a which are the main acid sensors in the brain. Some studies have shown that removing or inhibiting ASIC1a by genetic ablation reduces neuronal death (Xiong et al., 2004). More recently, in 2017, Hi1a, isolated from the Australian funnel-web spider *Hadronyche infensa*, was shown to be a potent inhibitor of ASIC1a. The real revolution of this peptide is its protection of the brain from neuronal damage for 8 h after a stroke event, instead of “only” 2–4 h for other potential drugs such as psalmotoxin 1 (PcTx1) from *Psalmopoeus cambridgei* (Chassagnon et al., 2017). Hi1a has a high sequence similarity to PcTx1, but is a more potent inhibitor, and is more selective with no effect on ASIC2a and ASIC3 channels. As a brief aside, in addition to its neuroprotective activity, PcTx1 is also of interest for reducing cartilage destruction in rheumatoid arthritis, in which ASIC1 plays a key role (Saez and Herzig, 2019). Hi1a has the ideal characteristics to be a therapeutic candidate. Very recently, the Australian government announced the next steps for the development of this peptide as the first spider-based drug. The search for other ASIC inhibitors continues with the Hm3a (*Heteroscodra maculata*) identification, which shows some similarities to PcTx-1. Both completely block ASIC1a with high potency (IC_50_ PcTx-1 ≃ 0.9 nM and IC_50_ Hm3a ≃ 1.3 nM) and have a lower activity for ASIC1b (EC_50_ ≃ 46.5 nM for both). A key advantage of Hm3a over the other drug candidates is its better biological stability (Er et al., 2017).

#### 3.2.3 Cancer

**FIGURE 7:**
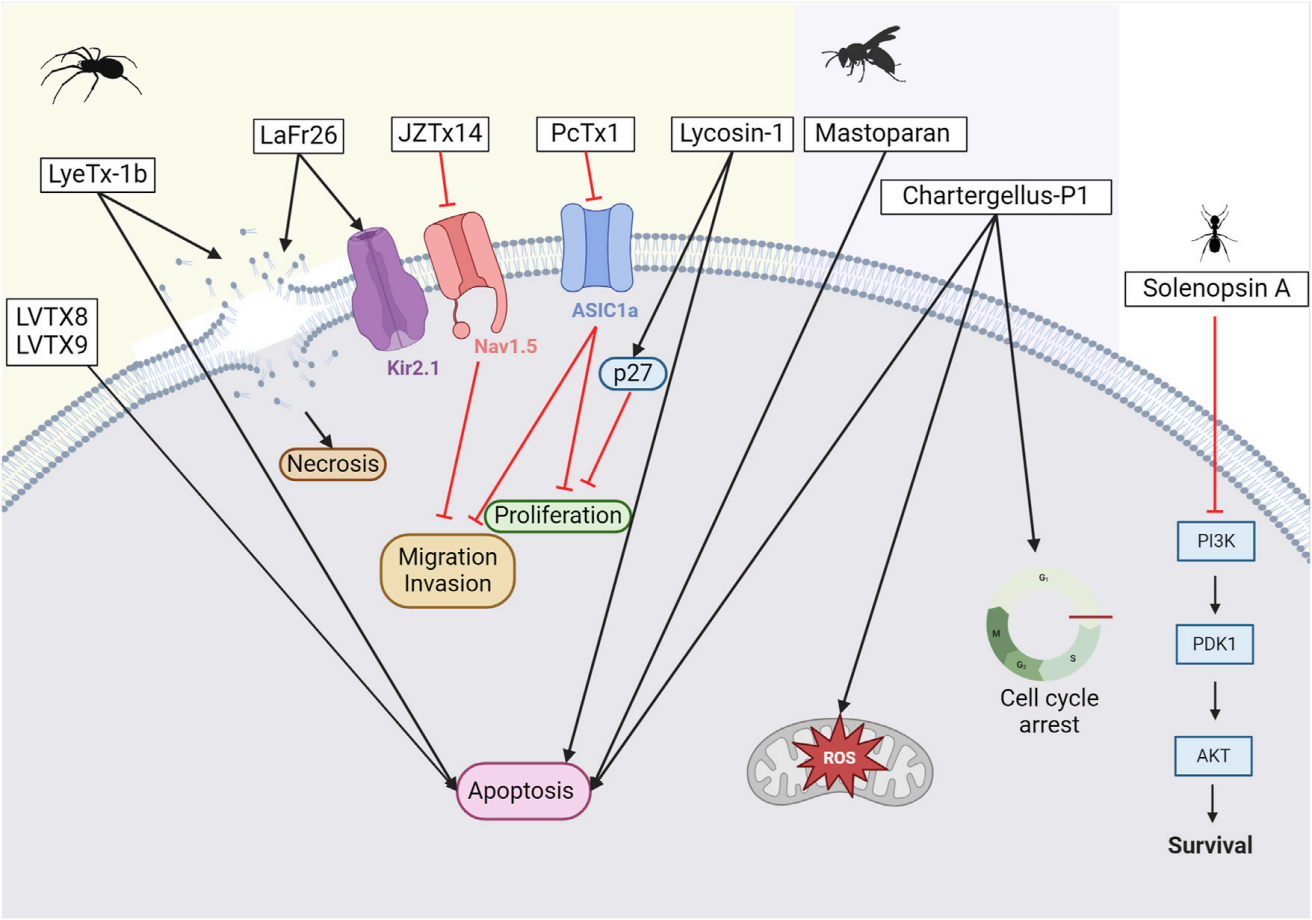
Potential anticancer toxins from other Arthropods. From left to right, an overview of some toxins with anti-cancer activity from spiders, wasps, and ants. From spider venoms, LVTX8 and LVTX9 can trigger apoptosis in some cancer cells (Zhang P. et al., 2020). LyeTx-1b has been demonstrated to either create pores leading to necrosis or trigger apoptosis (Abdel-Salam et al., 2019). LaFr26 is a pore-forming peptide specific to the Kir2 channel (Okada et al., 2019). JZTx14 is an inhibitor of the Na_V_1.5 channel. Inhibiting this channel leads to a reduction in the migration and invasion of cancer cells (Zhang et al., 2018). PcTx1 is an inhibitor of the ASIC1a channel. Its inhibition reduces cell proliferation (Rooj et al., 2012). Lycosin-1 can upregulate the p27 protein, which reduces cancer cell proliferation (Liu et al., 2012; Tan et al., 2016). However, it can also trigger apoptosis. From wasp venoms, mastoparan induces apoptosis, while chartergellus-P1 can increase reactive oxygen species and induce cell cycle arrest (de Azevedo et al., 2015; Soares et al., 2022). From ant venom, solenopsin A blocks PI3k and its downstream pathway (Arbiser et al., 2007). Created with BioRender.com (2024).

The potential of spider toxins has also been explored in cancer treatment (see Figure 7). Indeed, the venom of *Chilobrachys jingzhao* has been shown to have the ability to inhibit voltage-gated sodium channels. This is the case of JZTx-14, which was first reported by Zhang in 2018, who demonstrated its ability to block current flow in voltage-gated sodium (Na_V_1.2–1.8) channels (Zhang et al., 2018). Having observed the pro-metastatic effects of Na_V_1.5 and knowing that inhibitors of Na_V_1.5 are seen as emerging therapeutic candidates for breast cancer, Wu and colleagues conducted tests to analyze the potential of the peptide as an inhibitor of this channel in triple-negative breast cancer cells (Luo et al., 2020; Wu et al., 2023). Although this peptide did not reduce cancer cell proliferation, an inhibition of cancer cell migration by affecting the extracellular matrix and cell adhesion molecules was observed.

It has also been shown that ASIC1a expression is altered in gliomas. Consequently, inhibition of ASIC1a with PcTx-1 can reduce the proliferation and migration of glioma cells (Rooj et al., 2012). Notably, reducing ASIC1a expression in other types of cancer cells can also limit proliferation, migration, and invasion (Jin et al., 2015). Due to its remarkable selectivity, PcTx-1 has also recently been used as a true pharmacological tool to identify the ASIC1 subtype associated with breast cancer progression (Yang et al., 2020).

With more than 200 species described to date, spiders of the genus *Lycosa* have been extensively studied for this purpose. Lycosin-I peptide, derived from a toxin identified in the venom of *Lycosa singorensis*, a spider found in Central and Eastern Europe, has shown promise as a potential treatment option. Lycosin-I inhibits cancer cell growth *in vitro* by inducing programmed cell death (Liu et al., 2012). It sensitizes cancer cells to apoptosis and inhibits their proliferation by upregulating the cyclin-dependent kinase inhibitor 1B, p27, whose major function is to stop the cell cycle at the G1 phase. The mechanisms through which this peptide interacts with membrane cancer cells were investigated by Tan and colleagues (Tan et al., 2016). Furthermore, in 2018, Shen and colleagues demonstrated that lycosin-I inhibits the invasion and metastasis of prostate cancer cells (Shen et al., 2018). To improve the delivery of lycosin-I to cancer cells, the amino acid sequence of the peptide was modified by replacing a lysine with an arginine (Zhang et al., 2017). This change improved the interaction between R-lycosin I and the cancer cell membrane. The selectivity against cancer cells was then improved, while the IC_50_ against non-cancerous cells remained stable. In addition to amino acid modifications, various fatty acids were incorporated at the N-terminus of the R-lycosin I peptide to enhance its anticancer activity (Jian et al., 2018). The cytotoxicity of the obtained lipopeptide R-C16 with a 16-carbon fatty acid chain was three times higher for cancer cells than that of the original peptide. This was mainly due to the increased hydrophobicity, which enhanced the interaction between the peptide and the cell membrane. In 2017, Tan and colleagues created lycosin-I-modified spherical gold nanoparticles to improve intracellular delivery and were shown to accumulate in cancer cells, *in vitro* and *in vivo* (Tan et al., 2017). This suggests a high potential for clinical application in cancer therapy. Gold nanoparticles have been developed for selective targeting of cancer cells, as they can accumulate at tumor sites via non-specific receptor-mediated endocytosis. These particles can be applied locally and activated by laser light via the hyperthermia principle to penetrate directly into the tumor (Vines et al., 2019). Recently, the same team successfully developed lycosin-I-inspired fluorescent gold nanoparticles for tumor cell bioimaging (Tan et al., 2021). In parallel, a lycosin-I peptide coupled to HCPT, a DNA topoisomerase I inhibitor, has been developed (Zhang Q. et al., 2020). This conjugate forms in-solution nanospheres that enhance its antitumor and antimetastatic activity both *in vitro* and *in vivo*.

Another peptide, LyeTx I, from another species of the genus *Lycosa*, *Lycosa erythrognatha*, was synthesized and evaluated already in 2009 (Santos et al., 2010). This peptide was initially assessed for its antimicrobial properties, against *Staphylococcus aureus*, *Escherichia coli*, and *Candida krusei*. Despite a mild hemolytic activity, LyeTx I is a promising candidate for potential clinical applications. In 2018, an optimized peptide known as LyeTx I-b was prepared by incorporating a deletion of the His at position 16 and acetylating the N-terminus (Reis et al., 2018). The new compound exhibits antimicrobial activity that is 10 times higher than the native peptide. LyeTx I-b is not only interesting for its antimicrobial activity but also for its antitumor activity on brain tumor cells (Abdel-Salam et al., 2019). Interestingly, the IC_50_ values are lower for cancer cells (U-87 MG, glioblastoma cells) than for normal cells (<30 µM *versus* >100 µM), indicating a selectivity for the cancer cells. Notably, there was no effect on either apoptosis or autophagy in normal cells. However, exposure to IC_50_ treatment for a short period (approximately 15 min) degrades the integrity of cell membranes. This observation was confirmed by electron microscopy, which revealed pores, holes, and slits indicative of necrotic cell death (Abdel-Salam et al., 2019). The LyeTx I-b peptide has also been studied for its selectivity in degrading breast cancer cells (Abdel-Salam et al., 2021; de Avelar Junior et al., 2022). Exposure to this peptide induced apoptotic death in breast cancer cells but not in glioblastoma ones. Interestingly, systemic injection of the peptide into mice did not result in toxicity to the liver, kidneys, brain, spleen, or heart. Hematological parameters remained normal. *In vitro* studies confirmed that the peptide has antitumor activity and reduces tumor size. In addition, the peptide was found to have an immunomodulatory effect, reducing the number of monocytes, lymphocytes, neutrophils, and eosinophils. This discovery was significant because it demonstrated the involvement of leukocytes in tumor migration and metastasis. Moreover, the combined use of LyeTx-Ib and the chemotherapeutic agent cisplatin showed an increase in selectivity and a synergistic effect in a triple-negative breast cancer cell line, MDA-MB-231(de Avelar Junior et al., 2022). The combination of LyeTx-Ib and cisplatin showed reduced nephrotoxicity compared to cisplatin alone. Cisplatin treatment is associated with significant side effects, with nephrotoxicity occurring in more than 20%–30% of patients. These recent positive results are promising for future clinical trials.

The last *Lycosa* species under review is *Lycosa vittata*, mainly found in Southwestern China. Two interesting peptides have been described from its venom, LVTX-8 and LVTX-9. Both showed cytotoxic activity and the ability to induce apoptosis in lung carcinoma cells (A549 and H460) (Zhang P. et al., 2020). Furthermore, RNA sequencing analysis of treated and control samples showed that regulation of the p53 pathway inhibited cancer cell growth and migration. These findings were further validated in a mouse model of metastasis. More recently, analogs of LVTX-8 were shown to increase stability and resistance to protease degradation (Chi et al., 2023). Similarly, LVTX-9 was derived from the *Lycosa vittata* venom gland cDNA library (Li et al., 2021). However, this peptide exhibits lower levels of cytotoxicity against cancer cells. Chemical modifications involving the addition of fatty acids of different lengths to the N-terminus of LVTX-9 significantly increased the hydrophobicity of the peptides and, in turn, their cytotoxicity. LVTX-9-C18 showed the highest cytotoxicity due to an 18-carbon fatty acid inclusion in its sequence.

The potential effects of tarantula venom on cancer cells have been extensively studied. Of particular note is SNX-482, derived from the African tarantula *Hysterocrates gigas*. The 41 amino acids peptide, first reported in 1998 (Newcomb et al., 1998), is known to affect the influx of ion channels, specifically, the Ca_V_2.3 subunit-containing R-type calcium channel. However, the role of this channel in cancer initiation and progression is not fully understood. A study investigating the effects of SNX-482 on macrophages has shown that the peptide activates M0-macrophages, and increases molecules involved in antigen presentation, unraveling its potential for cancer immunotherapy (Munhoz et al., 2021).

So far, *Lachesana* spiders have revealed two peptides of interest: LaFr26 and latarcin-3a. LaFr26 is a pore-forming peptide that can conduct ions, like ionophores (Okada et al., 2015). Notably, this peptide was revealed to be specific for HEK293T cells overexpressing the inwardly rectifying K^+^ (Kir2.1) channel. Therefore, LaFr26 may be a remarkable choice for hyperpolarized K^+^ channel expressing cancer cells. This has been demonstrated later and confirmed for two lung cancer cell lines, LX22 and BEN (Okada et al., 2019). The second peptide, latarcin-3a, was first described in 2006 (Kozlov et al., 2006). Various latarcins have been discovered in the venom of *Lachesana tarabaevi*, with numerous effects noted (for a detailed review, see Dubovskii et al. (2015). For its anticancer properties, the amino acids of the latarcin-3a peptide have recently been modified to increase its hydrophobicity and net charge, resulting in increased antitumor activity (de Moraes et al., 2022).

#### 3.2.4 Muscular dystrophy

GsMTx4, a modulator of mechanosensitive ion channels (MSCs), was isolated from the tarantula *Grammostola rosea* (Gnanasambandam et al., 2017). This peptide has great potential for the treatment of Duchenne Muscular Dystrophy (DMD), a fatal orphan muscle disease for which there is currently no treatment. DMD is caused by a mutation in the gene encoding the dystrophin protein, resulting in a reduction or an absence of this protein and increased activation of MSCs (Ward et al., 2018). Interestingly, GsMTx4 can modulate the MSCs associated with dystrophin deficiency without affecting the MSCs involved in hearing and touch. This clear advantage, combined with its non-toxicity, non-immunogenicity, and high stability, makes it a good therapeutic candidate for DMD (Sachs, 2015). GsMTx4 has been in clinical development since 2014 and has been renamed AT-300 (Saez and Herzig, 2019).

### 3.3 Hymenoptera venoms, beyond melittin

Hymenoptera is an order that includes several species of bees, ants, and wasps and contains over 150,000 species. Hymenoptera venoms are composed of toxins and non-toxic components, such as inorganic salts, sugars, formic acid, free amino acids, hydrocarbons, peptides, and proteins (Guido-Patino and Plisson, 2022). Honeybee (*Apis mellifera*) venom has been widely studied for many years for its potential in a wide range of treatments, particularly for its antimicrobial activity. The venom consists of peptides, with melittin being the major compound, bioactive amines, non-peptide compounds, and enzymes such as hyaluronidase and PLA2 (group III) (Son et al., 2007).

#### 3.3.1 Neurological disorders

Group III PLA2s have real potential in the treatment of neurodegenerative diseases, such as prion, Parkinson’s, and Alzheimer’s diseases. Prion disease involves the accumulation of a misfolded, β-sheet-enriched isoform (PrPSc) of cellular prion protein (PrPC). The misfolded isoform is partially resistant to protease digestion, and forms aggregated and detergent-insoluble polymers in the CNS (Saverioni et al., 2013). Neuronal cell death caused by prion peptides can be prevented by PLA2s, which reduce PrP (106–126)-mediated neurotoxicity (Jeong et al., 2011). In Alzheimer’s disease, an Aβ peptide aggregation occurs, leading to neuroinflammation with microgliosis. PLA2s, found in bee venom, aid in suppressing microglial activation, leading to reduced cognitive and neuroinflammatory responses (Ye et al., 2016). PLA2s also offer therapeutic potential for Parkinson’s disease. This neurodegenerative disorder is characterized by a progressive degeneration of dopaminergic neurons in the substantia nigra. As in Alzheimer’s disease, neuroinflammatory mechanisms are involved in neuronal degeneration (Hirsch and Hunot, 2009) and PLA2s have a beneficial neuroprotective effect by increasing the survival of dopaminergic neurons. They can also induce the activation of regulatory T cells (Tregs) (Chung et al., 2015).

#### 3.3.2 Cancers

While bee venom products, such as melittin, have been extensively studied for their effects on cancer cells, this review will focus on other Hymenoptera species that possess anticancer activities (Pandey et al., 2023). Ant venom has received limited attention in cancer treatment. The red imported fire ant (RIFA), *Solenopsis invicta Buren*, is a widely distributed invasive species responsible for painful stings annually reported. The venom of this species consists primarily of non-peptide piperidine alkaloids called solenopsins and other noxious substances (Mo et al., 2023). Studies have shown that solenopsin A can reduce angiogenesis in a zebrafish model (Arbiser et al., 2007). Treatment *in vitro* appears to block the activation of Akt and PI3k, thereby regulating their downstream pathway. This PI3k/Akt pathway is well known to play a role in cancer cell growth, survival, and carcinogenesis (see Figure 7).

Wasp venoms additionally contain various small bioorganic molecules including amines (such as histamine and dopamine), proteins, and peptides (such as mastoparan and waspkinin) (Souza et al., 2005). Two peptides, polybia-MPI, and polybia-CP, have been isolated from the venom of the *Polybia paulista* wasp. Both have demonstrated cytotoxic effects against prostate and bladder cancer cells (see Figure 7) (Souza et al., 2005; Wang et al., 2008; Wang et al., 2011). More recently, a couple of groups attempted to improve polybia-MPI by mutating an amino acid or by engineering bacterial outer membrane vesicles to enhance its delivery to the tumor site (Phuong et al., 2023; Ren et al., 2023). Mastoparan is a small peptide discovered in wasp venom. Since then, more than 40 different mastoparan sequences have been identified (de Santana et al., 2022). Mastoparan from *Vespa* wasps and *Vespula* hornets has been shown to have cytotoxic effects against various cancer cells, including melanoma, breast adenocarcinoma, and glioblastoma (de Azevedo et al., 2015). It has also been observed to act synergistically with the chemotherapeutic agent gemcitabine in a mouse model of breast cancer (Hilchie et al., 2016). The mechanisms involved are related to mitochondrial-dependent apoptosis. Finally, chartergellus-P1 was isolated from the wasp *Chartergellus communis* and shares more than 90% homology with the polybia-CP peptide (Soares et al., 2022). As expected, the peptide exhibits cytotoxicity against two breast cancer cell lines (MCF-7 and MDA-MB-231), primarily by inducing cell cycle arrest, promoting apoptosis, and increasing intracellular reactive oxygen species levels.

## 4 Cone snail venom, an incredible pharmacological toolbox

**FIGURE 8:**
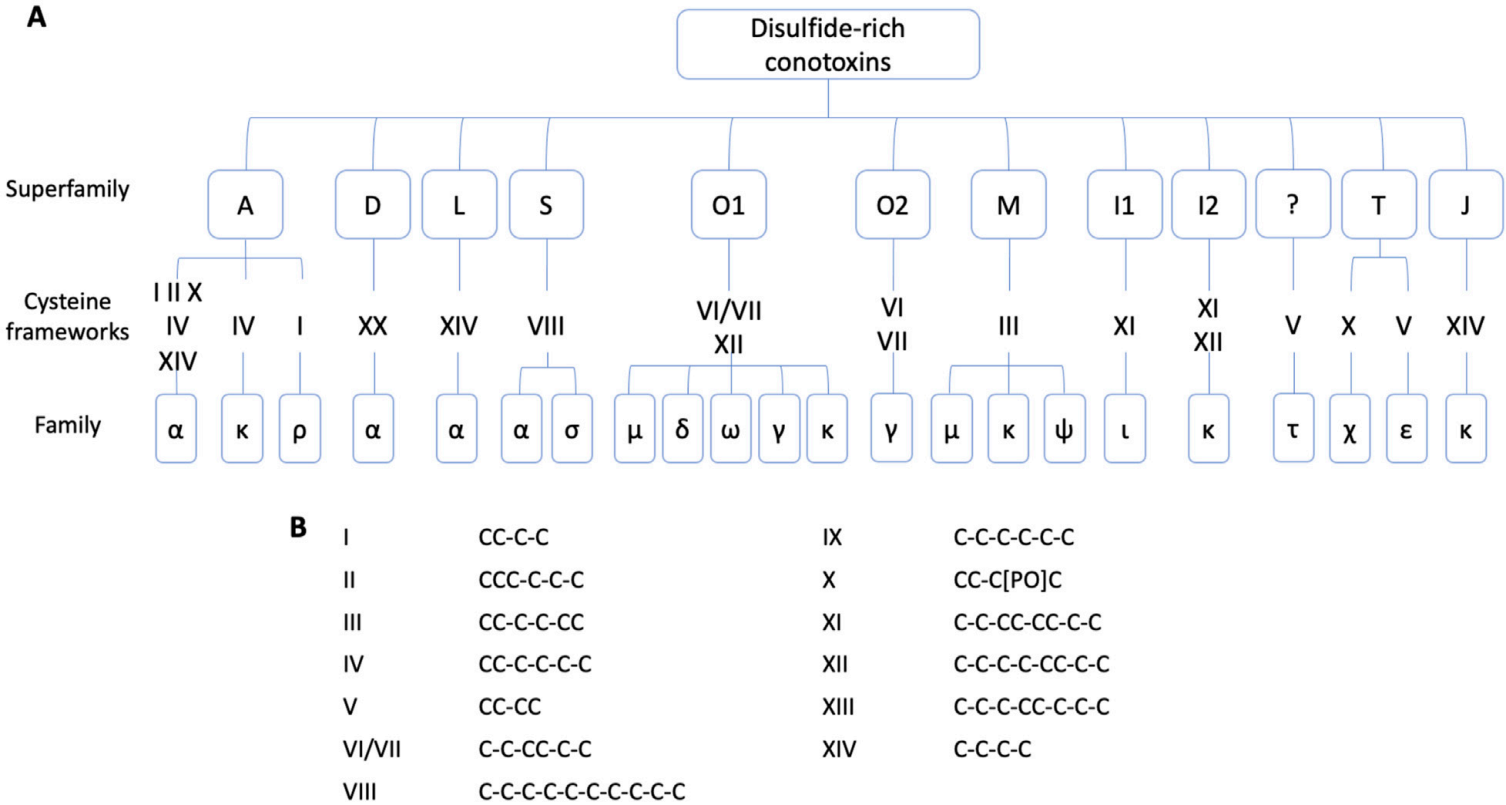
Classification of disulfide-rich conotoxins according to their cysteine frameworks. **(A)** Conotoxins are classified according to their pharmacological family, defined by a Greek letter, their cysteine framework, and their gene superfamily, represented by an Arabic capital letter (Akondi et al., 2014; Ratibou et al., 2024). **(B)** Conotoxin cysteine frameworks and their cysteine pattern.

Cone snails are specialized sea and ocean predators that use their venom to paralyze and hunt fishes, mollusks, and worms. Cone snail venoms represent a rich source of potent pharmacological compounds. It is reasonably estimated that more than 100 different peptides are produced in each venom. With 800 species, the cone snail venom library can be considered as a source of more than 80,000 bioactive peptides (Terlau and Olivera, 2004; Kohn, 2018). Cone snail venoms contain hundreds of small neurotoxic peptides (usually less than 5 kDa) that can be divided into two main groups based on the number of disulfide bonds: disulfide-poor (sometimes called conopeptides), with one or fewer disulfide bonds, and disulfide-rich, with two or more disulfide bonds (Mohan et al., 2020). Disulfide-poor peptides are usually less abundant in venom than disulfide-rich peptides, and include various subgroups with various targets, as shown in Table 7 (Lebbe and Tytgat, 2016). Disulfide-rich conotoxins are highly structured and often have high affinity for membrane receptors and ion channels, see Table 8 and Figure 8. Structural properties such as the number of disulfide bridges and the cysteine backbone, are important for the target interaction. Conotoxins are named following a convention of first the Greek letter related to their pharmacological target, second the initials of the conus species, next a Roman number related to the cysteine framework, and finally a capital letter for the order of discovery (Morales Duque et al., 2019; Ratibou et al., 2024). Cone snail venom has a rich pharmacological potential. However, only a small percentage (0.2%) of the components of these venoms have been studied so far, leaving much to be discovered (Peigneur et al., 2019).

**TABLE 7:** Disulfide-poor peptide families.

Family	Disulfide bond	Target	Examples	References for more details
Contulakins	0	Neurotensin receptors	Contulakin-G (*Conus geographus*): Not available in PDB AFDB accession: AF-Q9XYR5-F1	Robinson and Norton (2014) Lebbe and Tytgat (2016)
Conantokins	0	N-methyl-D-aspartate receptors	Conantokin-G (*Conus geographus*): 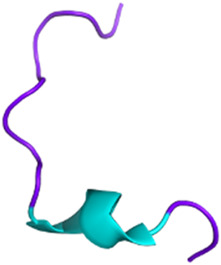 PDB 1AD7	
Conorfamides	0	RFamide receptors	CNF-Tx2 (*Conus textile*): 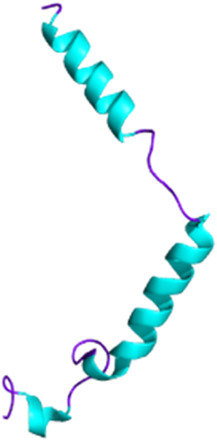 PDB 8JGB	
Conolysins	0	Cellular membranes	Conopressin (*Conus monile*): Not available in PDB AFDB accession: AF-Q9XYR5-F1	
Conopressins	1	Vasopressin receptors	Contulakin-G (*Conus geographus*): Not available in PDB AFDB accession: AF-A0A4Y5X186-F1	
Contryphans	1	Ca_V_ or K_V_ channels	Contryphan-R (*Conus radiatus*): 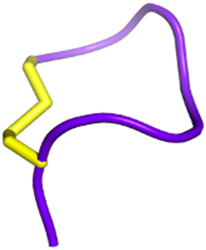 PDB 1QFB	
Conophans	0	Unknown	Conophan mus-V (*Conus mus*): Not available in PDB or AF	
Conomarphins	0	Unknown	Conomaprhin (*Conus marmoreus*): 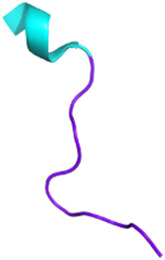 PDB 2JQB	
Conomaps	0	Unknown	Conomap-Vt (*Conus planorbis*): Not available in PDB or AF	
ConoCAPs	1	Unknown	ConoCAP-a (*Conus villepinii*): Not available in PDB AFDB accession: AF-E3PQQ8 -F1	
Cono-NPYs	0	Unknown	Cono-NPY (*Conus betulinus*): Not available in PDB or AF	
CONOGAYs	1	Unknown	ConoGAY-AusB (*Conus australis*): Not available in PDB AFDB accession: AF-P0DOZ2 -F1	

**TABLE 8:** Disulfide-rich peptide families.

Family	Disulfide bond number	Pharmacological target	Examples	References for more details
α-conotoxins	2	nAChRs	PnIA (*Conus pennaceus*): 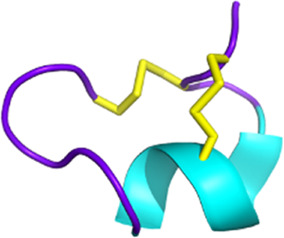 PDB 7N1Z	Lluisma et al. (2008) Robinson and Norton (2014) Ratibou et al. (2024)
	3	nAChRs	SII (*Conus striatus*): 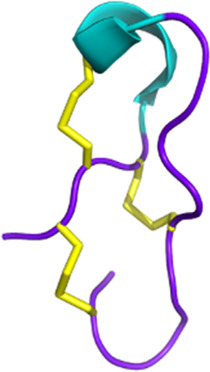 PDB 6OTB	
γ-conotoxins	3	Neuronal pacemaker cation currents	Gamma-conotoxin-like TEA53 (*Conus textile*): Not available in PDB AFDB accession: AF-AFQ3YEG0-F1	
δ-conotoxins	3	Na^+^ channels	TXVIA (*Conus textile*): 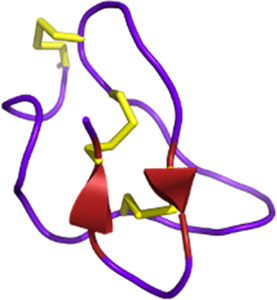 PDB 1FU3	
ε-conotoxins	2	G-Protein coupled receptors or Ca^2+^ channels	TxIX (*Conus textile*): 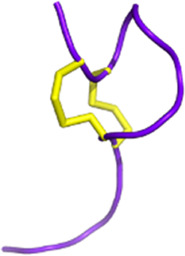 PDB 1WCT	
ι-conotoxins	4	Na^+^ channels	RXIA (*Conus radiatus*): 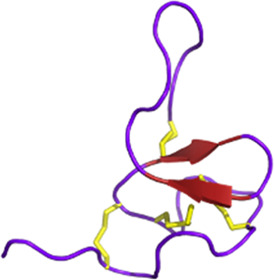 PDB 2JTU	
κ-conotoxins	3	K^+^ channels	PVIIA (*Conus purpurascens*): 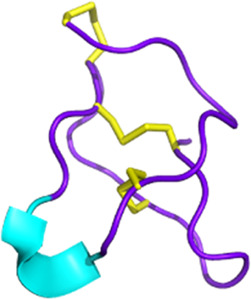 PDB 1AV3	
µ-conotoxins	3	Na^+^ channels	GIIIA (*Conus geographus*): 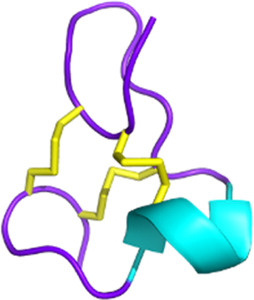 PDB 1TCG	
ρ-conotoxins	2	α-adrenoceptors	TIA (*Conus tulipa*): 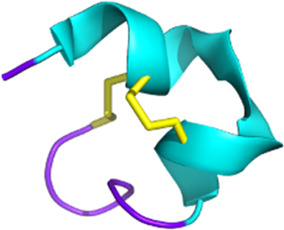 PDB 2LR9	
σ-conotoxins	5	Serotonin gated ion channels 5-HT_3_	GVIIIA (*Conus geographus*): Not available in PDB AFDB accession: AF-P58924-F1	
τ-conotoxins	2	Somatostatin receptors	CnVA (*Conus consors*): Not available in PBD or AF	
χ-conotoxins	2	Norepinephrine (NE) transporters	MrIA (*Conus marmoreus*): 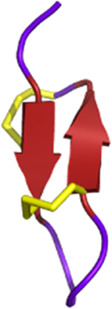 PDB 2EW4	
ψ-conotoxins	3	nAChRs	PIIIE (*Conus purpurascens*): 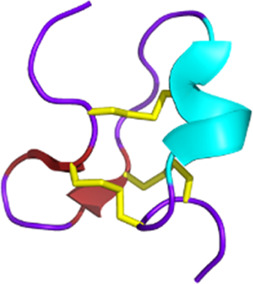 PDB 1JLO	
ω-conotoxins	3	Ca^2+^ channels	MVIIA (*Conus striatus*): 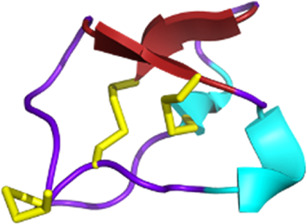 PDB 1MVJ	
φ-conotoxins	4	Granulin activity	MiXXVIIA (*Conus miles*): 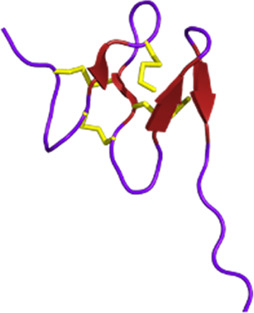 PDB 6PPC	

### 4.1 Chronic pain

Cone snail venom is well-known because it gave birth to the development of a famous drug called ziconotide (Prialt^©^). The latter, approved by the FDA in 2004, is used to treat chronic pain (as an analgesic). This venom-derived drug has the same sequence and structure as the ω-conotoxin MVIIA isolated and characterized from *Conus magus* venom (McIntosh et al., 1982; Miljanich, 2004). MVIIA is a blocker of the N-type Ca_v_2.2 voltage-gated calcium channel. Ca_v_2.2 is a validated target for the treatment of neuropathic pain. They are highly expressed in primary somatosensory afferent neurons, and the ventral horn and are then involved in synaptic transmission in ascending pain pathways (Kutzsche et al., 2024). α-RgIA (*Conus regius*) also shows an inhibitory activity on N-type Ca_v_2.2 channels with the same mechanism of action as MVIIA, explaining why this conotoxin could also be a promising treatment for neuropathic pain (Margiotta et al., 2022). Conotoxins, like many peptides, often cannot cross the blood-brain barrier, as is the case with ziconotide. Although it cannot then be administered systemically, but rather intrathecally, it has the advantage that its efficacy does not diminish over time, unlike opioid analgesics (morphine) (Gazerani and Cairns, 2014).

The χ-conotoxin family is known to target norepinephrine transporters (NETs) involved in neurological disorders, including neuropathic pain. χ-MrIA analog (Xen2174), isolated from *Conus marmoreus,* is an inhibitor of the norepinephrine transporter (NET) and shows high selectivity for this transporter (IC_50_ χ-MrIA = 645 nM). The synthetic analog has been tested for the treatment of chronic neuropathic pain in post-operative and cancer patients (Nielsen et al., 2005). Although the phase 2 clinical trial showed promising results, it did not pass phase 2b due to dose-limiting toxicity (Coulter-Parkhill et al., 2021) (see Table 3). The compound contulakin-G (16 residues), found in the venom of *Conus geographus*, entered clinical trials but was ultimately discontinued (see Table 3). It acts as an agonist of the neurotensin receptor subunit of the NMDA receptor (hNTR1) (Craig et al., 1999; Coulter-Parkhill et al., 2021).

### 4.2 Type-2 diabetes

Con-Ins G1 is an insulin-like peptide isolated from the venom of *Conus geographus*. This insulin molecule can activate the human insulin receptor (hIR) (see Figure 6). Unlike human insulin, Con-Ins G1 has a lower affinity for the primary binding site of the hIR and instead has a preferential affinity for the secondary binding site. This suggests a different mode of activation of the hIR (Safavi-Hemami et al., 2015; Xiong et al., 2020). The main problem with type-2 diabetes treatments (sulfonylureas, meglitinides, thiazolidines, GLP-1 mimetics, etc*.*) is that they are all associated with side effects, such as weight gain and hypoglycemia (Dobrica et al., 2019). Con-Ins G1 has led to the development of new recombinant analogs with a rapid onset of action due to their smaller size. The small size of the peptide makes chemical synthesis less complex, making it a strong candidate for a new human insulin treatment. Con-Ins G1 could become an important option among clinically approved insulin analogs (Xiong et al., 2020). The identification of conotoxin-like insulins opens the way to the study of cones and other marine species venoms. The comparison of sequence and structural features of human, zebrafish, and cone insulin provides a solid basis for exploring the diversity of conotoxin-like insulins to advance drug development efforts (Guo et al., 2024).

Conk-S1, isolated from the venom of *Conus striatus*, has shown a selective inhibitory activity for K_V_1.7 beta-cell channels. Insulin secretion is directly related to the electrical activity of the beta cell. Inhibition of this channel allows an increase in glucose-stimulated insulin secretion. Notably, the Conk-S1 inhibits the K_V_1.7 channel without causing hypoglycemia. Therefore, this conotoxin is of interest as a potential new therapeutic option, or at least to help characterize the mechanism of K_V_1.7 channels involved in insulin secretion, as this is still little understood (Finol-Urdaneta et al., 2012).

### 4.3 Channelopathies

Among ligand-gated ion channels, nicotinic acetylcholine receptors (nAChRs) and N-methyl-D-aspartate NMDA receptors have the greatest potential as lead compounds for new receptor therapies. nAChRs are found in both the peripheral and central nervous systems. They regulate the flow of sodium, potassium, and calcium ions across the cell membrane. These receptors mediate various cognitive processes and synaptic transmission from nerves to muscles (Hurst et al., 2013). As such, they play many important roles in the nervous system. There are 17 subtypes identified according to the combination of the five transmembrane subunits: α1 to α10, β1 to β4, γ, δ, and ε. Depending on the receptor subtype, and its localization, different disorders can be associated with them (Colombo et al., 2013). The main disorders are neurological, such as Alzheimer’s disease, schizophrenia, Parkinson’s disease, and depression, but nAChRs are also involved in nicotine addiction, and nicotine-induced behaviors, and are associated with small cell lung cancer (Ho et al., 2020). Cone venoms have evolved numerous classes of conopeptides that selectively target these channels. Among all the conotoxin families, no less than seven target the nAChR. The most abundant are the α-conotoxins (Lebbe et al., 2014). Within this family of conotoxins, the α3/5 targets muscle subtype nAChRs while α4/3, α4/4, α4/5, α4/6, and α4/7 conotoxins target neuronal nAChRs (Bekbossynova et al., 2021). For example, α-GI, an α3/5 targeting muscle nAChR subunit, isolated from *Conus geographus*, could be used as an alternative to a muscle relaxant administered during surgery (Tuba et al., 2002). The data show that α-GI targets the α/δ interface of the muscle nAChR with over 10,000 times higher affinity than the α/γ interface in mouse muscle. However, for the Torpedo nAChR, α-GI has a much higher affinity for the α/γ interface compared to the α/δ interface (Bekbossynova et al., 2021). Vc1.1 (ACV-1), from *Conus victoriae*, is a neuronal antagonist of α9α10 nAChRs, it was of interest as an analgesic for the treatment of neuropathic pain, but its efficacy did not reach the expected level, and phase II clinical trials were discontinued (see Figure 4; Table 3) (Sandall et al., 2003; Bordon et al., 2020). Another conotoxin MilIA, derived from *Conus milneedwardsi*, also shows activity for nAChR. MilIA is an α-conotoxin with a 3/5 framework. The potency against the muscle-type nAChR composed of α1β1γδ or α1β1εδ subunits, depending on the stage of development, fetal or adult, is rather low (IC_50_ fetal = 13,130 ± 1,125 nM and IC_50_ adult = 1,118 ± 78,891 nM). Synthetic analogs, MilIA [M9G] and MilIA [N10K], were then generated with a 23-fold and 3-fold improvement in potency, respectively. These analogs show selectivity for the fetal muscle type nAChR (Peigneur et al., 2019).

### 4.4 Cancers

**FIGURE 9:**
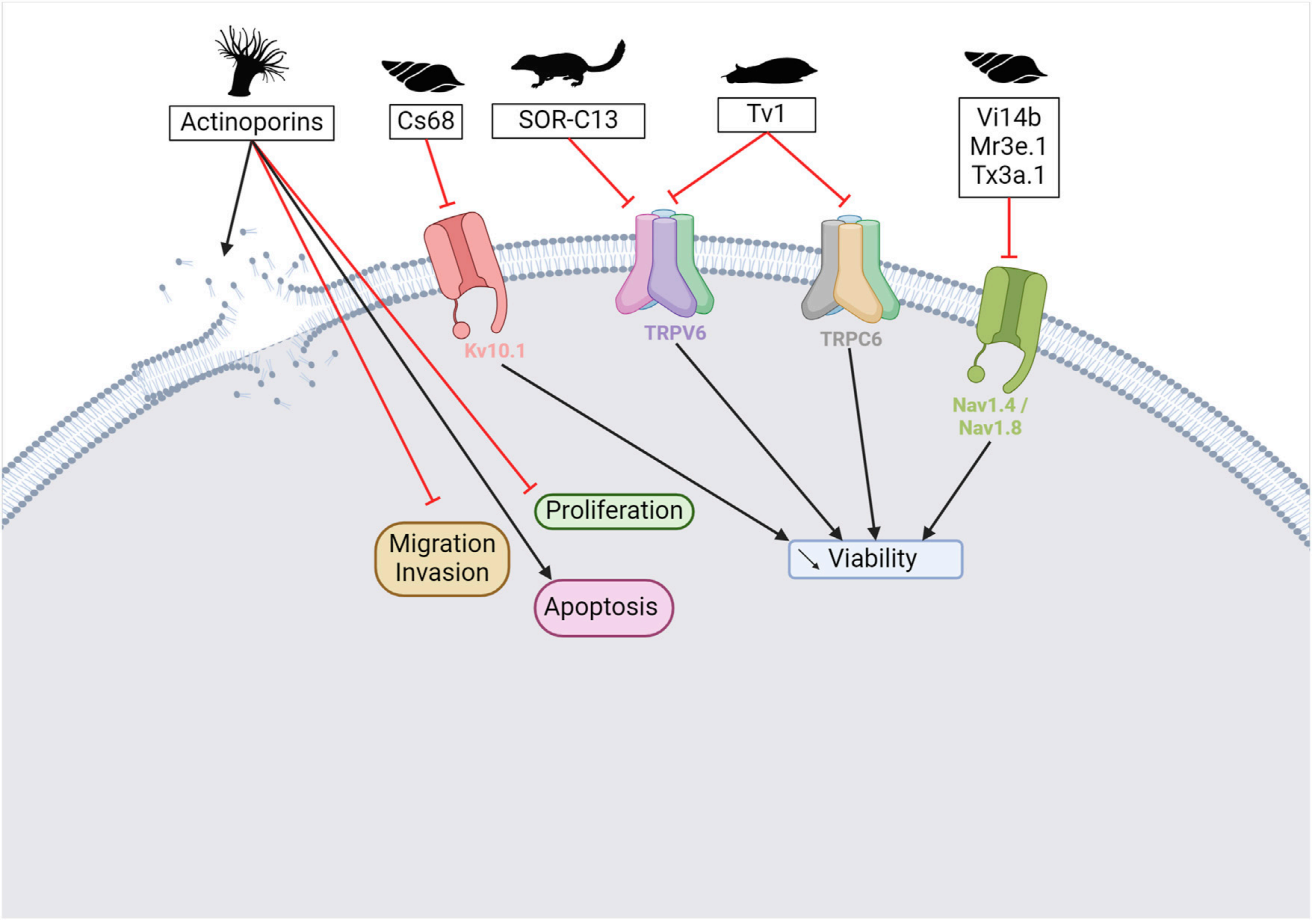
Potential anticancer peptides from cone snails and other species. Actinoporins from sea anemone venoms can form pores in cancer cell membranes but also reduce migration, invasion, and proliferation and trigger apoptosis (Kvetkina et al., 2020). Cs68 from *Conus spurius* inhibits the K_V_10.1 channel, leading to a reduction in cell viability—same observation for Vi14b, Mr3e.1, and Tx3a.1 that inhibit Na_V_1.4 and Na_V_1.8 channels (Martinez-Hernandez et al., 2023; Ju et al., 2022). SOR-C13 extracted from soricidin inhibits the TRPV6 channel (Stewart, 2018). Tv1 from the marine snail *Terebra variegate* can modulate the activity of TRPV6 and/or TRPC6 channels (Anand et al., 2019). Overall, specific mechanisms of action from those peptides remain to be elucidated. Created with BioRender.com (2024).

The conopeptide Cs68, discovered in the venom of *Conus spurius*, inhibits the oncogenic voltage-gated potassium K_V_10.1 channel, suggesting its potential as a therapeutic option (see Figure 9) (Martinez-Hernandez et al., 2023). It is worth noting that the efficacy of the peptide on cancer cells has not yet been investigated. Noteworthy, this peptide was also shown to inhibit K_V_11.1 and K_V_1.5. Similarly, three conotoxins (Vi14b, Mr3e.1, and Tx3a.1) isolated from *Conus virgo*, *Conus marmoreus*, and *Conus textile* exhibit anti-ovarian cancer activity and inhibit two voltage-gated sodium channels (Na_V_1.4 and Na_V_1.8) (Ju et al., 2022). This observation is directly related to the overexpression of voltage-gated sodium channels in ovarian cancer cells (Gao et al., 2010). However, the mechanisms by which these conotoxins reduce ovarian cancer cell viability are currently unknown and require further investigation.

### 4.5 Another conoidea member with anticancer activity

Terebridae belong to the same superfamily as Conidae. Its venom is understudied. Tv1, a peptide from the marine snail *Terebra variegate*, shows anticancer properties on hepatocellular carcinoma cells. Its efficacy is based on the modulation of the activity of TRPC6 and/or TRPV6 channels (Anand et al., 2019), which are involved in carcinogenesis. Administration of Tv1 to mice resulted in a reduction in tumor size.

## 5 Other venomous species of interest

### 5.1 Leeches

Leeches belong to the *Hirudinae* family. They are hematophagous, predatory, or parasitic feeders. There are over 700 species found worldwide, except Antarctica (Phillips et al., 2020). Their saliva -so not literally a venom- contains proteins with anticoagulant properties that prevent blood clotting and allow them to feed for long periods.

Hirudin, a protein isolated from the saliva of the leech *Hirudo medicinalis*, is a direct thrombin inhibitor (DTI). This peptide has an anticoagulant effect, but its irreversible inactivation of thrombin causes more bleeding than heparin (Warkentin, 2004). To improve the interaction with the active site of thrombin, some analogs have been designed based on the structure of hirudin. As a result, bivalirudin (Hirulog^®^), a 20 amino acid peptide, was developed and used as a clinical drug as it can reversibly bind to thrombin (Lee and Ansell, 2011).

### 5.2 Heloderma

There are only five known species of Heloderma. This family of venomous animals is found in the Southwestern United States, Mexico, and Central America. The venom of these venomous lizards is produced by their pre-mandibular glands and released through specialized grooved teeth. These animals use their venom for defense and for hunting small animals (Russell and Bogert, 1981). Proteomic studies of *Heloderma* venoms have highlighted various proteins and peptides such as hyaluronidase, CRiSP, exendin, helokinestatin, helofensin, kallikrein-like proteases, PLA_2_ (type III), and B-type natriuretic peptide (Koludarov et al., 2014). Helokinestatin and helofensin, which release toxic bioactive peptides after proteolytic degradation, are specific to venomous lizards (Sanggaard et al., 2015).

The venom of the Gila monster, *Heloderma suspectum*, contains a peptide homologous to the mammalian glucagon-like peptide 1 (GLP-1), exendin-4, which then binds to the incretin hormone GLP-1 receptor, a GPCR (Ki 0.46 nM). Because GLP-1 plays an important role in maintaining healthy blood glucose levels, a synthetic homolog of exendin-4, called exenatide (Byetta^®^), was developed in 2005 for type-2 diabetes treatment (see Figure 6). Exenatide induces insulin release, inhibits glucagon secretion, delays gastric emptying, and then suppresses appetite (Nadkarni et al., 2014). The first marketed drug derived from these observations, although effective, had an increased risk of pancreatitis. Therefore, other drugs have been developed to compensate for this problem (Lyxumia^®^ and Bydureon^®^) (Nauck et al., 2021). The development of these peptides is particularly interesting because it is the first treatment to target a metabolic function.

### 5.3 Cnidaria

Cnidaria includes sea anemones, sea pens, corals, jellyfish, and hydra, all of which are venomous and use their venom for predation and defense. There are about 10,000 aquatic species worldwide. They contain a collagen-filled capsule with venom and a thread-like tubule. This latter expands upon external mechanical or chemical stimulation. Most of the tubules can penetrate the skin and inject the venom contained in the capsule. Cnidaria venom is composed of enzymes, such as phospholipase A2 and metalloproteases, pore-forming toxins with actinoporins and perforin, and some neurotoxins that target potassium (KTxs) and sodium (NaTxs) channels, inhibitors of ASIC and transient receptor potential cation channel subfamily V member 1 (TRPV1), and Kunitz peptides (Jouiaei et al., 2015).

#### 5.3.1 Cancer

Sea anemone venoms are a rich source of peptides with exceptional pharmacological properties. Several peptides have been identified as useful for cancer research (see Figure 9). U-AITx-Ate1, isolated from the Australian sea anemone *Actinia tenebrosa*, is one of them. This peptide shows cytotoxicity against two breast cancer cell lines (MCF-7 and MDA-MB-231) (Elnahriry et al., 2019). However, the underlying mechanisms responsible for this cytotoxicity have not yet been investigated. Among the sea anemone toxins, the actinoporins are of particular interest. These toxins have been shown to reduce the proliferation and migration of several cancer cells, including leukemia, cervical cancer, breast cancer, and colon cancer. It can also induce apoptosis and reduce colony formation. The results show the potential of this toxin for cancer treatment (Kvetkina et al., 2020). However, since actinoporins are cytolytic by forming pores in cell membranes, their implementation in the clinic will require adaptations to reduce potential systemic toxicity.

#### 5.3.2 Channelopathies

As mentioned in the scorpion section, K_V_1.3 channels are implicated in several autoimmune diseases, such as multiple sclerosis, type 1 diabetes mellitus, and rheumatoid arthritis, where the ion channel plays a role in T-cell activation. *Stichodactyla helianthus*, a Caribbean Sea anemone, presents a toxin, ShK, that binds to this receptor at picomolar concentrations (Kd 11 pM) (Chandy et al., 2004). This potency is attractive for drug development, but a lack of selectivity reduces this interest. Indeed, ShK also presents an affinity for K_V_1.1 and K_V_1.6 at subnanomolar concentrations and for K_V_1.2, K_V_3.2, and K_Ca_3.1 in the nanomolar range. As a result, nearly 400 analogs have been produced (Chandy et al., 2023). ShK-186, called dalazatide, is the most promising of these analogs and has reached phase 2 clinical trials for psoriasis treatment (Olsen et al., 2016) (see Table 3).

Cnidaria venom contains various toxins that target ion channels, such as Na_V,_ K_V,_ and ASIC, which are involved in neurodegenerative diseases. According to Liao *et al*., the use of cnidaria toxins is underexploited, although this venom is a rich source of ion channel blockers, as presented in this review (Liao et al., 2019).

### 5.4 Mammalia

It is a recurring mistake to forget that venomous animals are also found among mammals, including monotremes (platypuses), eulipotyphla (shrews), chiropterans (vampire bats), and primates (pygmy lorises).

The monotreme order includes the platypus and four species of echidna. The male platypus (O*rnithorhynchus anatinus*) has a venom gland located on its hind leg. During the breeding season, the venom is used against competing males (Wong et al., 2012). The purpose of echidna venom is not yet understood. This venom is composed of C-type natriuretic peptides, defensin-like peptides, nerve growth factors, hyaluronidase, protease, and uncharacterized proteins (Ligabue-Braun et al., 2012). Monotreme venom also contains an analog of glucagon-like peptide-1 (pGLP-1) (Tsend-Ayush et al., 2016).

Soricidin is a peptide derived from the northern short-tailed shrew, *Blarina brevicauda* that has been shown to bind to the TRPV6 calcium channel (Stewart et al., 2006; Bowen et al., 2013). Two peptides, SOR-C13 and SOR-C17, extracted from soricidin, exhibit an affinity for ovarian and prostate tumors, enriched in TRPV6 channels (see Figure 9) (Stewart, 2018). These peptides’ conjugation with chemotherapeutics or contrast agents could further expand their use in diagnostic and therapeutic applications. SOR-C13 is in phase 1 of clinical development for the treatment of solid tumors (NCT01578564, NCT03784677) (Fu et al., 2017).

## 6 Limitations and future of venom-based drug discovery

Despite the large number of toxins with affinity for receptors involved in a variety of problems, and therefore a definite therapeutic potential, it can be observed that few venom-derived toxins are transformed into therapeutic successes. Many clinical trials are discontinued for lack of specificity, bioavailability, or efficacy (see Table 3). The difficulties in translating the *in vitro* pharmacological performance of Na_V_1.7 channels into effective *in vivo* analgesic outcomes were described in 2022 (Eagles et al., 2022). The challenge arises from the complexity of biological systems and the differences between controlled cellular environments and whole living organisms (Kim et al., 2020; Eagles et al., 2022). Indeed, the predictability of preclinical models is one of the causes explaining clinical trial failure (Gerard et al., 2021). Furthermore, research on receptors as therapeutic targets has highlighted the importance of understanding complex molecular interactions and specific mechanisms of action.

However, new possibilities are emerging now that artificial intelligence (AI) overturns how we work and think. Deep learning techniques like Molecular Contrastive Learning (MolCLR) and AlphaFold 3 can help predict molecular properties, protein structures, and how proteins interact with other molecules with high precision, thereby facilitating the discovery of new drugs (Jumper et al., 2021; Wang et al., 2022; Desai et al., 2024). These advanced technologies have the potential to discover venom-peptide analogs that efficiently target receptors, thus overcoming the limitation of *in vitro* testing. Moreover, 3D modeling applications could facilitate the development of toxins as therapeutics, enabling them to mimic the desired toxin activity without associated toxicity or to mitigate “off-target” effects. A data augmentation method, combining Generative Adversarial Networks (GAN) and Convolutional Neural Networks (CNN), was used recently to predict novel spider neurotoxic peptides (Lee et al., 2021). Deep learning tools, such as Graph Neural Networks (GNN) and Variational Autoencoders (VAE), can model the interaction between the peptide and the target at a granular level, providing valuable insights for designing more effective molecules (Seo et al., 2021; Li et al., 2022). In the context of venom-based drug discovery, these same technologies can create new chemical compounds inspired by venom with optimal pharmacological properties. DeepLPI, using Bidirectional Long Short-Term Memory (BiLSTM) can predict crucial protein-ligand interactions for therapeutic efficacy (Wei et al., 2022). The application of deep learning and AI tools in venom research provides a comprehensive understanding of intricate biological interactions and accelerates the drug discovery process. These technologies can overcome existing barriers by providing precise predictions and enabling faster, cost-effective testing (Bedraoui et al., 2024). The limitations in venom-based drug discovery highlight the importance of *in vivo* validation and the integration of advanced technologies to overcome obstacles in biomedical research.

## 7 Conclusion

In summary, venomous animals have evolved complex venom systems over millions of years, creating an extensive molecular arsenal that serves multiple biological functions beyond predation and defense. These venoms, rich in peptides and proteins, have proven highly effective in targeting specific molecular mechanisms, making them valuable candidates for drug development. The structural properties of venom peptides, such as their stability and selective binding capabilities, highlight their potential to create novel therapeutic agents. With 11 venom-derived drugs already approved for the treatment of various diseases and numerous studies underway, the potential of venom in medical applications is becoming increasingly evident. As research continues to explore the diverse bioactive compounds within venom, we can expect significant advances in the treatment of both cancer and non-cancer diseases by exploiting the unique properties of these naturally evolved molecules.

## References

[B1] Abdel-Ghani L. M. Rahmy T. R. Tawfik M. M. Kaziri I. Al-Obaidi A. Rowan E. G. (2019). Cytotoxicity of Nubein6.8 peptide isolated from the snake venom of Naja nubiae on melanoma and ovarian carcinoma cell lines. Toxicon 168, 22–31. 10.1016/j.toxicon.2019.06.220 31233771

[B2] Abdel-Salam M. A. L. Carvalho-Tavares J. Gomes K. S. Teixeira-Carvalho A. Kitten G. T. Nyffeler J. (2019). The synthetic peptide LyeTxI-b derived from Lycosa erythrognatha spider venom is cytotoxic to U-87 MG glioblastoma cells. Amino Acids 51 (3), 433–449. 10.1007/s00726-018-2678-4 30449002

[B3] Abdel-Salam M. A. L. Pinto B. Cassali G. Bueno L. Pegas G. Oliveira F. (2021). LyeTx I-b peptide attenuates tumor burden and metastasis in a mouse 4T1 breast cancer model. Antibiot. (Basel) 10 (9), 1136. 10.3390/antibiotics10091136 PMC8466574 34572719

[B4] Acharya K. R. Sturrock E. D. Riordan J. F. Ehlers M. R. (2003). Ace revisited: a new target for structure-based drug design. Nat. Rev. Drug Discov. 2 (11), 891–902. 10.1038/nrd1227 14668810 PMC7097707

[B5] Adams G. L. Pall P. S. Grauer S. M. Zhou X. Ballard J. E. Vavrek M. (2022). Development of ProTx-II analogues as highly selective peptide blockers of Na(v)1.7 for the treatment of pain. J. Med. Chem. 65 (1), 485–496. 10.1021/acs.jmedchem.1c01570 34931831

[B6] Agnarsson I. Coddington J. A. Kuntner M. (2013). “Systematics: progress in the study of spider diversity and evolution,” in Spider research in the 21st century: trends and perspectives (Siri Scientific Press), 58–111. P. D.

[B7] Aguiar F. L. L. Santos N. C. de Paula Cavalcante C. S. Andreu D. Baptista G. R. Goncalves S. (2020). Antibiofilm activity on Candida albicans and mechanism of action on biomembrane models of the antimicrobial peptide ctn[15-34]. Int. J. Mol. Sci. 21 (21), 8339. 10.3390/ijms21218339 33172206 PMC7664368

[B8] Aissaoui-Zid D. Saada M. C. Moslah W. Potier-Cartereau M. Lemettre A. Othman H. (2021). AaTs-1: a tetrapeptide from Androctonus australis scorpion venom, inhibiting U87 glioblastoma cells proliferation by p53 and FPRL-1 up-regulations. Molecules 26 (24), 7610. 10.3390/molecules26247610 34946686 PMC8704564

[B343] Akondi K. B. Muttenthaler M. Dutertre S. Kaas Q. Craik D. J. Lewis R. J. (2014). Discovery, synthesis, and structure-activity relationships of conotoxins. Chem. Rev. 114 (11), 5815–5847. 10.1021/cr400401e 24720541 PMC7610532

[B9] Alexandrou A. J. Brown A. R. Chapman M. L. Estacion M. Turner J. Mis M. A. (2016). Subtype-selective small molecule inhibitors reveal a fundamental role for Nav1.7 in nociceptor electrogenesis, axonal conduction and presynaptic release. PLoS One 11 (4), e0152405. 10.1371/journal.pone.0152405 27050761 PMC4822888

[B10] Almaaytah A. Albalas Q. (2014). Scorpion venom peptides with no disulfide bridges: a review. Peptides 51, 35–45. 10.1016/j.peptides.2013.10.021 24184590

[B11] Almeida C. F. Amaral C. Augusto T. V. Correia-da-Silva G. Marques de Andrade C. Torqueti M. R. (2021). The anti-cancer potential of crotoxin in estrogen receptor-positive breast cancer: its effects and mechanism of action. Toxicon 200, 69–77. 10.1016/j.toxicon.2021.07.003 34265323

[B12] Anand P. Filipenko P. Huaman J. Lyudmer M. Hossain M. Santamaria C. (2019). Selective inhibition of liver cancer cells using venom peptide. Mar. Drugs 17 (10), 587. 10.3390/md17100587 31627357 PMC6835663

[B13] Arbiser J. L. Kau T. Konar M. Narra K. Ramchandran R. Summers S. A. (2007). Solenopsin, the alkaloidal component of the fire ant (*Solenopsis invicta*), is a naturally occurring inhibitor of phosphatidylinositol-3-kinase signaling and angiogenesis. Blood 109 (2), 560–565. 10.1182/blood-2006-06-029934 16990598 PMC1785094

[B14] Ashcroft F. M. Rorsman P. (1989). Electrophysiology of the pancreatic β-cell. Prog. Biophys. Mol. Biol. 54 (2), 87–143. 10.1016/0079-6107(89)90013-8 2484976

[B15] Attarde S. S. Pandit S. V. (2017). Cytotoxic activity of NN-32 toxin from Indian spectacled cobra venom on human breast cancer cell lines. BMC Complement. Altern. Med. 17 (1), 503. 10.1186/s12906-017-2018-3 29183371 PMC5704554

[B16] Attarde S. S. Pandit S. V. (2020). Anticancer potential of nanogold conjugated toxin GNP-NN-32 from Naja naja venom. J. Venom. Anim. Toxins Incl. Trop. Dis. 26, e20190047. 10.1590/1678-9199-JVATITD-2019-0047 32180805 PMC7059613

[B17] Bandeira I. C. J. Bandeira-Lima D. Mello C. P. Pereira T. P. De Menezes R. Sampaio T. L. (2018). Antichagasic effect of crotalicidin, a cathelicidin-like vipericidin, found in *Crotalus durissus* terrificus rattlesnake's venom gland. Parasitology 145 (8), 1059–1064. 10.1017/S0031182017001846 29208061

[B18] Banerjee Y. Mizuguchi J. Iwanaga S. Kini R. M. (2005). Hemextin AB complex, a unique anticoagulant protein complex from Hemachatus haemachatus (African Ringhals cobra) venom that inhibits clot initiation and factor VIIa activity. J. Biol. Chem. 280 (52), 42601–42611. 10.1074/jbc.M508987200 16204244

[B19] Barnwal B. Jobichen C. Girish V. M. Foo C. S. Sivaraman J. Kini R. M. (2016). Ringhalexin from Hemachatus haemachatus: a novel inhibitor of extrinsic tenase complex. Sci. Rep. 6, 25935. 10.1038/srep25935 27173146 PMC4865804

[B20] Bedraoui A. Suntravat M. El Mejjad S. Enezari S. Oukkache N. Sanchez E. E. (2024). Therapeutic potential of snake venom: toxin distribution and opportunities in deep learning for novel drug discovery. Med. Drug Discov. 21, 100175. 10.1016/j.medidd.2023.100175

[B21] Bekbossynova A. Zharylgap A. Filchakova O. (2021). Venom-derived neurotoxins targeting nicotinic acetylcholine receptors. Molecules 26 (11), 3373. 10.3390/molecules26113373 34204855 PMC8199771

[B22] Bhattacharjee P. Bhattacharyya D. (2013). Factor V activator from Daboia russelli russelli venom destabilizes β-amyloid aggregate, the hallmark of alzheimer disease. J. Biol. Chem. 288 (42), 30559–30570. 10.1074/jbc.M113.511410 23986449 PMC3798527

[B23] Bhattacharya N. Kolvekar N. Mondal S. Sarkar A. Chakrabarty D. (2023). Biological activities of Vipegrin, an anti-adhesive Kunitz-type serine proteinase inhibitor purified from Russell's viper venom. Toxicon 232, 107213. 10.1016/j.toxicon.2023.107213 37419286

[B24] Bhowmik T. Gomes A. (2016). NKCT1 (purified Naja kaouthia protein toxin) conjugated gold nanoparticles induced Akt/mTOR inactivation mediated autophagic and caspase 3 activated apoptotic cell death in leukemic cell. Toxicon 121, 86–97. 10.1016/j.toxicon.2016.08.004 27527270

[B25] Bhowmik T. Gomes A. (2017). Down-regulation of cyclin-dependent kinase-4 and MAPK through estrogen receptor mediated cell cycle arrest in human breast cancer induced by gold nanoparticle tagged toxin protein NKCT1. Chem. Biol. Interact. 268, 119–128. 10.1016/j.cbi.2017.03.009 28322778

[B26] Bhowmik T. Saha P. P. Dasgupta A. Gomes A. (2013). Antileukemic potential of PEGylated gold nanoparticle conjugated with protein toxin (NKCT1) isolated from Indian cobra (Naja kaouthia) venom. Cancer Nanotechnol. 4 (1-3), 39–55. 10.1007/s12645-013-0036-5 26069500 PMC4451861

[B27] Bhowmik T. Saha P. P. Sarkar A. Gomes A. (2017). Evaluation of cytotoxicity of a purified venom protein from Naja kaouthia (NKCT1) using gold nanoparticles for targeted delivery to cancer cell. Chem. Biol. Interact. 261, 35–49. 10.1016/j.cbi.2016.11.007 27836789

[B28] Bledzka K. Smyth S. S. Plow E. F. (2013). Integrin alphaIIbbeta3: from discovery to efficacious therapeutic target. Circ. Res. 112 (8), 1189–1200. 10.1161/CIRCRESAHA.112.300570 23580774 PMC3711133

[B29] Boltman T. Meyer M. Ekpo O. (2023). Diagnostic and therapeutic approaches for glioblastoma and neuroblastoma cancers using chlorotoxin nanoparticles. Cancers (Basel) 15 (13), 3388. 10.3390/cancers15133388 37444498 PMC10341066

[B30] Bordon K. C. F. Cologna C. T. Fornari-Baldo E. C. Pinheiro-Junior E. L. Cerni F. A. Amorim F. G. (2020). From animal poisons and venoms to medicines: achievements, challenges and perspectives in drug discovery. Front. Pharmacol. 11, 1132. 10.3389/fphar.2020.01132 32848750 PMC7396678

[B31] Bowen C. V. DeBay D. Ewart H. S. Gallant P. Gormley S. Ilenchuk T. T. (2013). *In vivo* detection of human TRPV6-rich tumors with anti-cancer peptides derived from soricidin. PLoS One 8 (3), e58866. 10.1371/journal.pone.0058866 23554944 PMC3598914

[B32] Brown M. C. Staniszewska I. Del Valle L. Tuszynski G. P. Marcinkiewicz C. (2008). Angiostatic activity of obtustatin as α1β1 integrin inhibitor in experimental melanoma growth. Int. J. Cancer 123 (9), 2195–2203. 10.1002/ijc.23777 18712720 PMC2587450

[B33] Bychkov M. L. Kirichenko A. V. Shulepko M. A. Mikhaylova I. N. Kirpichnikov M. P. Lyukmanova E. N. (2021). Mambalgin-2 inhibits growth, migration, and invasion of metastatic melanoma cells by targeting the channels containing an ASIC1a subunit whose up-regulation correlates with poor survival prognosis. Biomedicines 9 (10), 1324. 10.3390/biomedicines9101324 34680442 PMC8533404

[B34] Bychkov M. L. Shulepko M. A. Vasileva V. Y. Sudarikova A. V. Kirpichnikov M. P. Lyukmanova E. N. (2020). ASIC1a inhibitor mambalgin-2 suppresses the growth of leukemia cells by cell cycle arrest. Acta Naturae 12 (2), 111–116. 10.32607/actanaturae.11158 PMC7385094 32742733

[B35] Calvete J. J. Marcinkiewicz C. Monleon D. Esteve V. Celda B. Juarez P. (2005). Snake venom disintegrins: evolution of structure and function. Toxicon 45 (8), 1063–1074. 10.1016/j.toxicon.2005.02.024 15922775

[B36] Campeiro J. D. Marinovic M. P. Carapeto F. C. Dal Mas C. Monte G. G. Carvalho Porta L. (2018). Oral treatment with a rattlesnake native polypeptide crotamine efficiently inhibits the tumor growth with no potential toxicity for the host animal and with suggestive positive effects on animal metabolic profile. Amino Acids 50 (2), 267–278. 10.1007/s00726-017-2513-3 29235017

[B37] Casewell N. R. Wuster W. Vonk F. J. Harrison R. A. Fry B. G. (2013). Complex cocktails: the evolutionary novelty of venoms. Trends Ecol. Evol. 28 (4), 219–229. 10.1016/j.tree.2012.10.020 23219381

[B38] Castle N. A. London D. O. Creech C. Fajloun Z. Stocker J. W. Sabatier J. M. (2003). Maurotoxin: a potent inhibitor of intermediate conductance Ca2+-activated potassium channels. Mol. Pharmacol. 63 (2), 409–418. 10.1124/mol.63.2.409 12527813

[B39] Castro-Amorim J. Novo de Oliveira A. Da Silva S. L. Soares A. M. Mukherjee A. K. Ramos M. J. (2023). Catalytically active snake venom PLA(2) enzymes: an overview of its elusive mechanisms of reaction. J. Med. Chem. 66 (8), 5364–5376. 10.1021/acs.jmedchem.3c00097 37018514 PMC10150362

[B40] Cavalcante C. S. Falcao C. B. Fontenelle R. O. Andreu D. Radis-Baptista G. (2017). Anti-fungal activity of Ctn[15-34], the C-terminal peptide fragment of crotalicidin, a rattlesnake venom gland cathelicidin. J. Antibiot. (Tokyo) 70 (3), 231–237. 10.1038/ja.2016.135 27876749

[B41] Chai J. Yang W. Gao Y. Guo R. Peng Q. Abdel-Rahman M. A. (2021). Antitumor effects of scorpion peptide Smp43 through mitochondrial dysfunction and membrane disruption on hepatocellular carcinoma. J. Nat. Prod. 84 (12), 3147–3160. 10.1021/acs.jnatprod.1c00963 34866381

[B42] Chandy K. G. Norton R. S. (2017). Peptide blockers of K(v)1.3 channels in T cells as therapeutics for autoimmune disease. Curr. Opin. Chem. Biol. 38, 97–107. 10.1016/j.cbpa.2017.02.015 28412597

[B43] Chandy K. G. Sanches K. Norton R. S. (2023). Structure of the voltage-gated potassium channel K(V)1.3: insights into the inactivated conformation and binding to therapeutic leads. Channels (Austin) 17 (1), 2253104. 10.1080/19336950.2023.2253104 37695839 PMC10496531

[B44] Chandy K. G. Wulff H. Beeton C. Pennington M. Gutman G. A. Cahalan M. D. (2004). K+ channels as targets for specific immunomodulation. Trends Pharmacol. Sci. 25 (5), 280–289. 10.1016/j.tips.2004.03.010 15120495 PMC2749963

[B45] Chassagnon I. R. McCarthy C. A. Chin Y. K. Pineda S. S. Keramidas A. Mobli M. (2017). Potent neuroprotection after stroke afforded by a double-knot spider-venom peptide that inhibits acid-sensing ion channel 1a. Proc. Natl. Acad. Sci. U. S. A. 114 (14), 3750–3755. 10.1073/pnas.1614728114 28320941 PMC5389327

[B46] Chen Z. Tran D. Li T. Arias K. Griffith B. P. Wu Z. J. (2020). The role of a disintegrin and metalloproteinase proteolysis and mechanical damage in nonphysiological shear stress-induced platelet receptor shedding. ASAIO J. 66 (5), 524–531. 10.1097/MAT.0000000000001028 31192844 PMC7323905

[B47] Chi Q. N. Jia S. X. Yin H. Wang L. E. Fu X. Y. Ma Y. N. (2023). Efficient synthesis and anticancer evaluation of spider toxin peptide LVTX-8-based analogues with enhanced stability. Bioorg Chem. 134, 106451. 10.1016/j.bioorg.2023.106451 36907048

[B48] Chiou J. T. Shi Y. J. Wang L. J. Huang C. H. Lee Y. C. Chang L. S. (2019). Naja atra cardiotoxin 3 elicits autophagy and apoptosis in U937 human leukemia cells through the Ca(2+)/PP2A/AMPK Axis. Toxins (Basel) 11 (9), 527. 10.3390/toxins11090527 31547294 PMC6784133

[B49] Chiou J. T. Wang L. J. Lee Y. C. Chang L. S. (2021). Naja atra cardiotoxin 1 induces the FasL/fas death pathway in human leukemia cells. Cells 10 (8), 2073. 10.3390/cells10082073 34440842 PMC8394927

[B50] Chong H. P. Tan K. Y. Tan C. H. (2020). Cytotoxicity of snake venoms and cytotoxins from two southeast asian cobras (Naja sumatrana, Naja kaouthia): exploration of anticancer potential, selectivity, and cell death mechanism. Front. Mol. Biosci. 7, 583587. 10.3389/fmolb.2020.583587 33263003 PMC7686564

[B51] Chung E. S. Lee G. Lee C. Ye M. Chung H. S. Kim H. (2015). Bee venom phospholipase A2, a novel Foxp3+ regulatory T cell inducer, protects dopaminergic neurons by modulating neuroinflammatory responses in a mouse model of Parkinson's disease. J. Immunol. 195 (10), 4853–4860. 10.4049/jimmunol.1500386 26453752

[B52] Ciolek J. Reinfrank H. Quinton L. Viengchareun S. Stura E. A. Vera L. (2017). Green mamba peptide targets type-2 vasopressin receptor against polycystic kidney disease. Proc. Natl. Acad. Sci. U. S. A. 114 (27), 7154–7159. 10.1073/pnas.1620454114 28630289 PMC5502595

[B53] Colombo S. F. Mazzo F. Pistillo F. Gotti C. (2013). Biogenesis, trafficking and up-regulation of nicotinic ACh receptors. Biochem. Pharmacol. 86 (8), 1063–1073. 10.1016/j.bcp.2013.06.023 23830821

[B54] Conde R. Zamudio F. Z. Rodriguez M. H. Possani L. D. (2000). Scorpine, an anti-malaria and anti-bacterial agent purified from scorpion venom. FEBS Lett. 471 (2-3), 165–168. 10.1016/s0014-5793(00)01384-3 10767415

[B55] Coulter-Parkhill A. McClean S. Gault V. A. Irwin N. (2021). Therapeutic potential of peptides derived from animal venoms: current views and emerging drugs for diabetes. Clin. Med. Insights Endocrinol. Diabetes 14, 117955142110060. 10.1177/11795514211006071 PMC8491154 34621137

[B56] Craig A. G. Norberg T. Griffin D. Hoeger C. Akhtar M. Schmidt K. (1999). Contulakin-G, an O-glycosylated invertebrate neurotensin. J. Biol. Chem. 274 (20), 13752–13759. 10.1074/jbc.274.20.13752 10318778

[B57] Cummins T. R. Sheets P. L. Waxman S. G. (2007). The roles of sodium channels in nociception: implications for mechanisms of pain. Pain 131 (3), 243–257. 10.1016/j.pain.2007.07.026 17766042 PMC2055547

[B58] Cura J. E. Blanzaco D. P. Brisson C. Cura M. A. Cabrol R. Larrateguy L. (2002). Phase I and pharmacokinetics study of crotoxin (cytotoxic PLA(2), NSC-624244) in patients with advanced cancer. Clin. Cancer Res. 8 (4), 1033–1041. 11948110

[B59] Cushman D. W. Ondetti M. A. (1991). History of the design of captopril and related inhibitors of angiotensin converting enzyme. Hypertension 17 (4), 589–592. 10.1161/01.hyp.17.4.589 2013486

[B60] Dalmolin G. D. Silva C. R. Rigo F. K. Gomes G. M. do Nascimento Cordeiro M. Richardson M. (2011). Antinociceptive effect of Brazilian armed spider venom toxin Tx3-3 in animal models of neuropathic pain. Pain 152 (10), 2224–2232. 10.1016/j.pain.2011.04.015 21570770

[B61] D'Amelio F. Vigerelli H. de Brandao Prieto da Silva A. R. Kerkis I. (2021). Bothrops moojeni venom and its components - an overview. J. Venom. Res. 11, 26–33. 34123362 PMC8169028

[B62] Dardevet L. Najlaoui F. Aroui S. Collot M. Tisseyre C. Pennington M. W. (2022). A conjugate between lqh-8/6, a natural peptide analogue of chlorotoxin, and doxorubicin efficiently induces glioma cell death. Biomedicines 10 (10), 2605. 10.3390/biomedicines10102605 36289865 PMC9599068

[B63] da Rocha R. G. Santos E. M. S. Tanaka-Azevedo A. M. Serino-Silva C. Souza M. G. Gomes E. S. B. (2023). The antineoplastic potential of crotoxin isolated from *Crotalus durissus* terrificus snake venom on oral squamous cell carcinoma. Toxicon 221, 106965. 10.1016/j.toxicon.2022.106965 36370827

[B64] Das T. Bhattacharya S. Biswas A. Gupta S. D. Gomes A. Gomes A. (2013). Inhibition of leukemic U937 cell growth by induction of apoptosis, cell cycle arrest and suppression of VEGF, MMP-2 and MMP-9 activities by cytotoxin protein NN-32 purified from Indian spectacled cobra (Naja naja) venom. Toxicon 65, 1–4. 10.1016/j.toxicon.2013.01.004 23337397

[B65] Das T. Bhattacharya S. Halder B. Biswas A. Das Gupta S. Gomes A. (2011). Cytotoxic and antioxidant property of a purified fraction (NN-32) of Indian Naja naja venom on Ehrlich ascites carcinoma in BALB/c mice. Toxicon 57 (7-8), 1065–1072. 10.1016/j.toxicon.2011.04.012 21530568

[B66] de Avelar Junior J. T. Lima-Batista E. Castro Junior C. J. Pimenta A. M. C. Dos Santos R. G. Souza-Fagundes E. M. (2022). LyeTxI-b, a synthetic peptide derived from a spider venom, is highly active in triple-negative breast cancer cells and acts synergistically with cisplatin. Front. Mol. Biosci. 9, 876833. 10.3389/fmolb.2022.876833 35601827 PMC9114809

[B67] de Azevedo R. A. Figueiredo C. R. Ferreira A. K. Matsuo A. L. Massaoka M. H. Girola N. (2015). Mastoparan induces apoptosis in B16F10-Nex2 melanoma cells via the intrinsic mitochondrial pathway and displays antitumor activity *in vivo* . Peptides 68, 113–119. 10.1016/j.peptides.2014.09.024 25305549

[B68] Debnath A. Saha A. Gomes A. Biswas S. Chakrabarti P. Giri B. (2010). A lethal cardiotoxic-cytotoxic protein from the Indian monocellate cobra (Naja kaouthia) venom. Toxicon 56 (4), 569–579. 10.1016/j.toxicon.2010.05.016 20595038

[B69] de Carvalho Porta L. Fadel V. D'Arc Campeiro J. Oliveira E. B. Godinho R. O. Hayashi M. A. F. (2020). Biophysical and pharmacological characterization of a full-length synthetic analog of the antitumor polypeptide crotamine. J. Mol. Med. Berl. 98 (11), 1561–1571. 10.1007/s00109-020-01975-y 32895732

[B70] de Moraes L. Silva P. S. E. Pereira T. Almeida Rodrigues T. A. Farias Frihling B. E. da Costa R. A. (2022). First generation of multifunctional peptides derived from latarcin-3a from Lachesana tarabaevi spider toxin. Front. Microbiol. 13, 965621. 10.3389/fmicb.2022.965621 36212827 PMC9532841

[B71] Deng Z. Gao Y. Nguyen T. Chai J. Wu J. Li J. (2023). The potent antitumor activity of Smp43 against non-small-cell lung cancer A549 cells via inducing membranolysis and mitochondrial dysfunction. Toxins (Basel) 15 (5), 347. 10.3390/toxins15050347 37235381 PMC10223799

[B72] Desai D. Kantliwala S. V. Vybhavi J. Ravi R. Patel H. Patel J. (2024). Review of AlphaFold 3: transformative advances in drug design and therapeutics. Cureus 16 (7), e63646. 10.7759/cureus.63646 39092344 PMC11292590

[B73] de Santana C. J. C. Pires Junior O. R. Fontes W. Palma M. S. Castro M. S. (2022). Mastoparans: a group of multifunctional α-helical peptides with promising therapeutic properties. Front. Mol. Biosci. 9, 824989. 10.3389/fmolb.2022.824989 35813822 PMC9263278

[B74] de Souza A. H. Lima M. C. Drewes C. C. da Silva J. F. Torres K. C. Pereira E. M. (2011). Antiallodynic effect and side effects of Phα1β, a neurotoxin from the spider Phoneutria nigriventer: comparison with ω-conotoxin MVIIA and morphine. Toxicon 58 (8), 626–633. 10.1016/j.toxicon.2011.09.008 21967810

[B75] De Waard S. Montnach J. Cortinovis C. Chkir O. Erfanian M. Hulin P. (2020). Maurocalcin and its analog MCaE12A facilitate Ca2+ mobilization in cardiomyocytes. Biochem. J. 477 (20), 3985–3999. 10.1042/BCJ20200206 33034621

[B76] Dintzis S. M. Hansen S. Harrington K. M. Tan L. C. Miller D. M. Ishak L. (2019). Real-time visualization of breast carcinoma in pathology specimens from patients receiving fluorescent tumor-marking agent tozuleristide. Arch. Pathol. Lab. Med. 143 (9), 1076–1083. 10.5858/arpa.2018-0197-OA 30550350 PMC11781288

[B77] Diochot S. Baron A. Salinas M. Douguet D. Scarzello S. Dabert-Gay A. S. (2012). Black mamba venom peptides target acid-sensing ion channels to abolish pain. Nature 490 (7421), 552–555. 10.1038/nature11494 23034652

[B78] Dobrica E. C. Gaman M. A. Cozma M. A. Bratu O. G. Pantea Stoian A. Diaconu C. C. (2019). Polypharmacy in type 2 diabetes mellitus: insights from an internal medicine department. Med. Kaunas. 55 (8), 436. 10.3390/medicina55080436 PMC6723949 31382651

[B79] Donato N. J. Martin C. A. Perez M. Newman R. A. Vidal J. C. Etcheverry M. (1996). Regulation of epidermal growth factor receptor activity by crotoxin, a snake venom phospholipase A2 toxin. Biochem. Pharmacol. 51 (11), 1535–1543. 10.1016/0006-2952(96)00097-4 8630095

[B80] Droctove L. Ciolek J. Mendre C. Chorfa A. Huerta P. Carvalho C. (2022). A new Kunitz-type snake toxin family associated with an original mode of interaction with the vasopressin 2 receptor. Br. J. Pharmacol. 179 (13), 3470–3481. 10.1111/bph.15814 35122240

[B81] Dubovskii P. V. Vassilevski A. A. Kozlov S. A. Feofanov A. V. Grishin E. V. Efremov R. G. (2015). Latarcins: versatile spider venom peptides. Cell Mol. Life Sci. 72 (23), 4501–4522. 10.1007/s00018-015-2016-x 26286896 PMC11113828

[B82] Duggan N. M. Saez N. J. Clayton D. Budusan E. Watson E. E. Tucker I. J. (2021). Total synthesis of the spider-venom peptide Hi1a. Org. Lett. 23 (21), 8375–8379. 10.1021/acs.orglett.1c03112 34632783

[B83] Eagles D. A. Chow C. Y. King G. F. (2022). Fifteen years of Na(V) 1.7 channels as an analgesic target: why has excellent *in vitro* pharmacology not translated into *in vivo* analgesic efficacy? Br. J. Pharmacol. 179 (14), 3592–3611. 10.1111/bph.15327 33206998

[B84] Elnahriry K. A. Wai D. C. C. Krishnarjuna B. Badawy N. N. Chittoor B. MacRaild C. A. (2019). Structural and functional characterisation of a novel peptide from the Australian sea anemone Actinia tenebrosa. Toxicon 168, 104–112. 10.1016/j.toxicon.2019.07.002 31302115

[B85] Elrayess R. A. Mohallal M. E. Mobarak Y. M. Ebaid H. M. Haywood-Small S. Miller K. (2021). Scorpion venom antimicrobial peptides induce caspase-1 dependant pyroptotic cell death. Front. Pharmacol. 12, 788874. 10.3389/fphar.2021.788874 35082671 PMC8784870

[B86] Er S. Y. Cristofori-Armstrong B. Escoubas P. Rash L. D. (2017). Discovery and molecular interaction studies of a highly stable, tarantula peptide modulator of acid-sensing ion channel 1. Neuropharmacology 127, 185–195. 10.1016/j.neuropharm.2017.03.020 28327374

[B87] Estrada-Gomez S. Gomez-Rave L. Vargas-Munoz L. J. van der Meijden A. (2017). Characterizing the biological and biochemical profile of six different scorpion venoms from the Buthidae and Scorpionidae family. Toxicon 130, 104–115. 10.1016/j.toxicon.2017.02.007 28209477

[B88] Falcao C. B. Perez-Peinado C. de la Torre B. G. Mayol X. Zamora-Carreras H. Jimenez M. A. (2015). Structural dissection of crotalicidin, a rattlesnake venom cathelicidin, retrieves a fragment with antimicrobial and antitumor activity. J. Med. Chem. 58 (21), 8553–8563. 10.1021/acs.jmedchem.5b01142 26465972

[B89] Faure G. Harvey A. L. Thomson E. Saliou B. Radvanyi F. Bon C. (1993). Comparison of crotoxin isoforms reveals that stability of the complex plays a major role in its pharmacological action. Eur. J. Biochem. 214 (2), 491–496. 10.1111/j.1432-1033.1993.tb17946.x 8513799

[B90] Ferreira S. H. Bartelt D. C. Greene L. J. (1970). Isolation of bradykinin-potentiating peptides from *Bothrops jararaca* venom. Biochemistry 9 (13), 2583–2593. 10.1021/bi00815a005 4317874

[B91] Finol-Urdaneta R. K. Remedi M. S. Raasch W. Becker S. Clark R. B. Struver N. (2012). Block of Kv1.7 potassium currents increases glucose-stimulated insulin secretion. EMBO Mol. Med. 4 (5), 424–434. 10.1002/emmm.201200218 22438204 PMC3403299

[B92] Flinspach M. Xu Q. Piekarz A. D. Fellows R. Hagan R. Gibbs A. (2017). Insensitivity to pain induced by a potent selective closed-state Nav1.7 inhibitor. Sci. Rep. 7, 39662. 10.1038/srep39662 28045073 PMC5206724

[B93] Formicola B. Dal Magro R. Montefusco-Pereira C. V. Lehr C. M. Koch M. Russo L. (2019). The synergistic effect of chlorotoxin-mApoE in boosting drug-loaded liposomes across the BBB. J. Nanobiotechnology 17 (1), 115. 10.1186/s12951-019-0546-3 31711496 PMC6844026

[B94] Fry B. G. Roelants K. Champagne D. E. Scheib H. Tyndall J. D. King G. F. (2009). The toxicogenomic multiverse: convergent recruitment of proteins into animal venoms. Annu. Rev. Genomics Hum. Genet. 10, 483–511. 10.1146/annurev.genom.9.081307.164356 19640225

[B95] Fu S. Hirte H. Welch S. Ilenchuk T. T. Lutes T. Rice C. (2017). First-in-human phase I study of SOR-C13, a TRPV6 calcium channel inhibitor, in patients with advanced solid tumors. Invest. New Drugs 35 (3), 324–333. 10.1007/s10637-017-0438-z 28150073 PMC5418314

[B96] Funk C. Gmur J. Herold R. Straub P. W. (1971). Reptilase®‐R—a new reagent in blood coagulation. Br. J. Haematol. 21 (1), 43–52. 10.1111/j.1365-2141.1971.tb03415.x 5105276

[B97] Gao R. Shen Y. Cai J. Lei M. Wang Z. (2010). Expression of voltage-gated sodium channel alpha subunit in human ovarian cancer. Oncol. Rep. 23 (5), 1293–1299. 10.3892/or_00000763 20372843

[B98] Gazerani P. Cairns B. E. (2014). Venom-based biotoxins as potential analgesics. Expert Rev. Neurother. 14 (11), 1261–1274. 10.1586/14737175.2014.962518 25234848

[B99] Gerard L. Duvivier L. Gillet J. P. (2021). Targeting tumor resistance mechanisms. Fac. Rev. 10, 6. 10.12703/r/10-6 33659924 PMC7894262

[B100] Ghazaryan N. Movsisyan N. Macedo J. C. Vaz S. Ayvazyan N. Pardo L. (2019). The antitumor efficacy of monomeric disintegrin obtustatin in S-180 sarcoma mouse model. Invest. New Drugs 37 (5), 1044–1051. 10.1007/s10637-019-00734-2 30680583

[B101] Ghazaryan N. A. Ghulikyan L. A. Kishmiryan A. V. Kirakosyan G. R. Nazaryan O. H. Ghevondyan T. H. (2015). Anti-tumor effect investigation of obtustatin and crude *Macrovipera lebetina* obtusa venom in S-180 sarcoma bearing mice. Eur. J. Pharmacol. 764, 340–345. 10.1016/j.ejphar.2015.07.011 26169565

[B102] Ghosh A. Roy R. Nandi M. Mukhopadhyay A. (2019). Scorpion venom-toxins that aid in drug development: a review. Int. J. Pept. Res. Ther. 25 (1), 27–37. 10.1007/s10989-018-9721-x 32214927 PMC7088386

[B103] Girish V. M. Kini R. M. (2016). Exactin: a specific inhibitor of Factor X activation by extrinsic tenase complex from the venom of Hemachatus haemachatus. Sci. Rep. 6, 32036. 10.1038/srep32036 27558950 PMC4997346

[B104] Gnanasambandam R. Ghatak C. Yasmann A. Nishizawa K. Sachs F. Ladokhin A. S. (2017). GsMTx4: mechanism of inhibiting mechanosensitive ion channels. Biophys. J. 112 (1), 31–45. 10.1016/j.bpj.2016.11.013 28076814 PMC5231890

[B105] Gomes G. M. Dalmolin G. D. Cordeiro M. doN. Gomez M. V. Ferreira J. Rubin M. A. (2013). The selective A-type K+ current blocker Tx3-1 isolated from the Phoneutria nigriventer venom enhances memory of naïve and Aβ25-35-treated mice. Toxicon official J. Int. Soc. Toxinology 76, 23–27. 10.1016/j.toxicon.2013.08.059 23994427

[B106] Gomis-Ruth F. X. Kress L. F. Bode W. (1993). First structure of a snake venom metalloproteinase: a prototype for matrix metalloproteinases/collagenases. EMBO J. 12 (11), 4151–4157. 10.1002/j.1460-2075.1993.tb06099.x 8223430 PMC413708

[B107] Gopal G. Muralidar S. Prakash D. Kamalakkannan A. Indhuprakash S. T. Thirumalai D. (2023). The concept of Big Four: road map from snakebite epidemiology to antivenom efficacy. Int. J. Biol. Macromol. 242 (Pt 1), 124771. 10.1016/j.ijbiomac.2023.124771 37169043

[B108] Graf N. Mokhtari T. E. Papayannopoulos I. A. Lippard S. J. (2012). Platinum(IV)-chlorotoxin (CTX) conjugates for targeting cancer cells. J. Inorg. Biochem. 110, 58–63. 10.1016/j.jinorgbio.2012.02.012 22465700 PMC3350571

[B109] Guido-Patino J. C. Plisson F. (2022). Profiling hymenopteran venom toxins: protein families, structural landscape, biological activities, and pharmacological benefits. Toxicon X 14, 100119. 10.1016/j.toxcx.2022.100119 35372826 PMC8971319

[B110] Guo M. Teng M. Niu L. Liu Q. Huang Q. Hao Q. (2005). Crystal structure of the cysteine-rich secretory protein stecrisp reveals that the cysteine-rich domain has a K+ channel inhibitor-like fold. J. Biol. Chem. 280 (13), 12405–12412. 10.1074/jbc.M413566200 15596436

[B111] Guo Q. Huang M. Li M. Chen J. Cheng S. Ma L. (2024). Diversity and evolutionary analysis of venom insulin derived from cone snails. Toxins (Basel) 16 (1), 34. 10.3390/toxins16010034 38251250 PMC10819828

[B112] Guo R. Chen X. Nguyen T. Chai J. Gao Y. Wu J. (2022). The strong anti-tumor effect of Smp24 in lung adenocarcinoma A549 cells depends on its induction of mitochondrial dysfunctions and ROS accumulation. Toxins (Basel) 14 (9), 590. 10.3390/toxins14090590 36136528 PMC9502404

[B113] Han R. Liang H. Qin Z. H. Liu C. Y. (2014). Crotoxin induces apoptosis and autophagy in human lung carcinoma cells *in vitro* via activation of the p38MAPK signaling pathway. Acta Pharmacol. Sin. 35 (10), 1323–1332. 10.1038/aps.2014.62 25132339 PMC4186982

[B114] Han S. Yi H. Yin S. J. Chen Z. Y. Liu H. Cao Z. J. (2008). Structural basis of a potent peptide inhibitor designed for Kv1.3 channel, a therapeutic target of autoimmune disease. J. Biol. Chem. 283 (27), 19058–19065. 10.1074/jbc.M802054200 18480054

[B115] Harel M. Kleywegt G. J. Ravelli R. B. Silman I. Sussman J. L. (1995). Crystal structure of an acetylcholinesterase-fasciculin complex: interaction of a three-fingered toxin from snake venom with its target. Structure 3 (12), 1355–1366. 10.1016/s0969-2126(01)00273-8 8747462

[B116] He J. K. Wu X. S. Wang Y. Han R. Qin Z. H. Xie Y. (2013). Growth inhibitory effects and molecular mechanisms of crotoxin treatment in esophageal Eca-109 cells and transplanted tumors in nude mice. Acta Pharmacol. Sin. 34 (2), 295–300. 10.1038/aps.2012.156 23202800 PMC4011616

[B117] Herrington J. (2007). Gating modifier peptides as probes of pancreatic β-cell physiology. Toxicon 49 (2), 231–238. 10.1016/j.toxicon.2006.09.012 17101164

[B118] Herrington J. Sanchez M. Wunderler D. Yan L. Bugianesi R. M. Dick I. E. (2005). Biophysical and pharmacological properties of the voltage‐gated potassium current of human pancreatic β‐cells. J. Physiol. 567 (Pt 1), 159–175. 10.1113/jphysiol.2005.089375 15932888 PMC1474166

[B119] Herzig V. Cristofori-Armstrong B. Israel M. R. Nixon S. A. Vetter I. King G. F. (2020). Animal toxins - nature's evolutionary-refined toolkit for basic research and drug discovery. Biochem. Pharmacol. 181, 114096. 10.1016/j.bcp.2020.114096 32535105 PMC7290223

[B120] Hilchie A. L. Sharon A. J. Haney E. F. Hoskin D. W. Bally M. B. Franco O. L. (2016). Mastoparan is a membranolytic anti-cancer peptide that works synergistically with gemcitabine in a mouse model of mammary carcinoma. Biochim. Biophys. Acta 1858 (12), 3195–3204. 10.1016/j.bbamem.2016.09.021 27693190 PMC5097029

[B121] Hirsch E. C. Hunot S. (2009). Neuroinflammation in Parkinson's disease: a target for neuroprotection? Lancet Neurol. 8 (4), 382–397. 10.1016/S1474-4422(09)70062-6 19296921

[B122] Hiu J. J. Yap M. K. K. (2021). The effects of Naja sumatrana venom cytotoxin, sumaCTX on alteration of the secretome in MCF-7 breast cancer cells following membrane permeabilization. Int. J. Biol. Macromol. 184, 776–786. 10.1016/j.ijbiomac.2021.06.145 34174307

[B123] Hmed B. Serria H. T. Mounir Z. K. (2013). Scorpion peptides: potential use for new drug development. J. Toxicol. 2013, 1–15. 10.1155/2013/958797 PMC3697785 23843786

[B124] Ho T. N. T. Abraham N. Lewis R. J. (2020). Structure-function of neuronal nicotinic acetylcholine receptor inhibitors derived from natural toxins. Front. Neurosci. 14, 609005. 10.3389/fnins.2020.609005 33324158 PMC7723979

[B125] Holford M. Daly M. King G. F. Norton R. S. (2018). Venoms to the rescue. Science 361 (6405), 842–844. 10.1126/science.aau7761 30166472

[B126] Hurst R. Rollema H. Bertrand D. (2013). Nicotinic acetylcholine receptors: from basic science to therapeutics. Pharmacol. Ther. 137 (1), 22–54. 10.1016/j.pharmthera.2012.08.012 22925690

[B127] Igarashi T. Araki S. Mori H. Takeda S. (2007). Crystal structures of catrocollastatin/VAP2B reveal a dynamic, modular architecture of ADAM/adamalysin/reprolysin family proteins. FEBS Lett. 581 (13), 2416–2422. 10.1016/j.febslet.2007.04.057 17485084

[B128] Inan S. Y. Yildirim S. Tanriover G. Ilhan B. (2024). P/Q type (Ca(v)2.1) calcium channel blocker omega-agatoxin IVA alters cleaved caspase-3 and BDNF expressions in the rat brain and suppresses seizure activity. Mol. Neurobiol. 61 (4), 1861–1872. 10.1007/s12035-023-03678-0 37798599

[B129] Izidoro L. F. Sobrinho J. C. Mendes M. M. Costa T. R. Grabner A. N. Rodrigues V. M. (2014). Snake venom L-amino acid oxidases: trends in pharmacology and biochemistry. Biomed. Res. Int. 2014, 1–19. 10.1155/2014/196754 PMC3971498 24738050

[B130] Jadvar H. Chen K. Park R. Yap L. P. Vorobyova I. Swenson S. (2019). Preclinical evaluation of a (64)Cu-labeled disintegrin for PET imaging of prostate cancer. Amino Acids 51 (10-12), 1569–1575. 10.1007/s00726-019-02794-3 31621030 PMC6881555

[B131] Jeong J. K. Moon M. H. Bae B. C. Lee Y. J. Seol J. W. Park S. Y. (2011). Bee venom phospholipase A2 prevents prion peptide induced-cell death in neuronal cells. Int. J. Mol. Med. 28 (5), 867–873. 10.3892/ijmm.2011.730 21701769

[B132] Jian C. Zhang P. Ma J. Jian S. Zhang Q. Liu B. (2018). The roles of fatty-acid modification in the activity of the anticancer peptide R-lycosin-I. Mol. Pharm. 15 (10), 4612–4620. 10.1021/acs.molpharmaceut.8b00605 30183307

[B133] Jin C. Ye Q. H. Yuan F. L. Gu Y. L. Li J. P. Shi Y. H. (2015). Involvement of acid-sensing ion channel 1α in hepatic carcinoma cell migration and invasion. Tumour Biol. 36 (6), 4309–4317. 10.1007/s13277-015-3070-6 25613068

[B134] Joubert F. J. Taljaard N. (1979). Some properties and the complete primary structures of two reduced and S-carboxymethylated polypeptides (S5C1 and S5C10) from Dendroaspis jamesoni kaimosae (Jameson's mamba) venom. Biochim. Biophys. Acta 579 (1), 228–233. 10.1016/0005-2795(79)90101-6 465532

[B135] Jouiaei M. Yanagihara A. A. Madio B. Nevalainen T. J. Alewood P. F. Fry B. G. (2015). Ancient venom systems: a review on Cnidaria toxins. Toxins (Basel) 7 (6), 2251–2271. 10.3390/toxins7062251 26094698 PMC4488701

[B136] Joviano-Santos J. V. Valadao P. A. C. Magalhaes-Gomes M. P. S. Fernandes L. F. Diniz D. M. Machado T. C. G. (2021). Protective effect of a spider recombinant toxin in a murine model of Huntington's disease. Neuropeptides 85, 102111. 10.1016/j.npep.2020.102111 33333486

[B137] Ju S. Zhang Y. Guo X. Yan Q. Liu S. Ma B. (2022). Anti-ovarian cancer conotoxins identified from Conus venom. Molecules 27 (19), 6609. 10.3390/molecules27196609 36235146 PMC9573077

[B138] Jumper J. Evans R. Pritzel A. Green T. Figurnov M. Ronneberger O. (2021). Highly accurate protein structure prediction with AlphaFold. Nature 596 (7873), 583–589. 10.1038/s41586-021-03819-2 34265844 PMC8371605

[B139] Kampo S. Ahmmed B. Zhou T. Owusu L. Anabah T. W. Doudou N. R. (2019). Scorpion venom analgesic peptide, BmK AGAP inhibits stemness, and epithelial-mesenchymal transition by down-regulating PTX3 in breast cancer. Front. Oncol. 9, 21. 10.3389/fonc.2019.00021 30740360 PMC6355678

[B140] Kampo S. Cui Y. Yu J. Anabah T. W. Falagan A. A. Bayor M. T. (2021). Scorpion Venom peptide, AGAP inhibits TRPV1 and potentiates the analgesic effect of lidocaine. Heliyon 7 (12), e08560. 10.1016/j.heliyon.2021.e08560 35005265 PMC8715296

[B141] Kang T. S. Georgieva D. Genov N. Murakami M. T. Sinha M. Kumar R. P. (2011). Enzymatic toxins from snake venom: structural characterization and mechanism of catalysis. FEBS J. 278 (23), 4544–4576. 10.1111/j.1742-4658.2011.08115.x 21470368

[B142] Kerkis I. Hayashi M. A. Prieto da Silva A. R. Pereira A. De Sa Junior P. L. Zaharenko A. J. (2014). State of the art in the studies on crotamine, a cell penetrating peptide from South American rattlesnake. Biomed. Res. Int. 2014, 1–9. 10.1155/2014/675985 PMC3914522 24551848

[B144] Kharrat R. Mabrouk K. Crest M. Darbon H. Oughideni R. Martin-Eauclaire M. F. (1996). Chemical synthesis and characterization of maurotoxin, a short scorpion toxin with four disulfide bridges that acts on K+ channels. Eur. J. Biochem. 242 (3), 491–498. 10.1111/j.1432-1033.1996.0491r.x 9022673

[B145] Kim J. Koo B. K. Knoblich J. A. (2020). Human organoids: model systems for human biology and medicine. Nat. Rev. Mol. Cell Biol. 21 (10), 571–584. 10.1038/s41580-020-0259-3 32636524 PMC7339799

[B146] King G. F. (2011). Venoms as a platform for human drugs: translating toxins into therapeutics. Expert Opin. Biol. Ther. 11 (11), 1469–1484. 10.1517/14712598.2011.621940 21939428

[B147] King G. F. (2013). Venoms to drugs: translating venom peptides into therapeutics. Aust. Biochem. 44 (3), 13–15.

[B148] King G. F. Hardy M. C. (2013). Spider-venom peptides: structure, pharmacology, and potential for control of insect pests. Annu. Rev. Entomol. 58, 475–496. 10.1146/annurev-ento-120811-153650 23020618

[B149] King G. F. Vetter I. (2014). No gain, no pain: NaV1.7 as an analgesic target. ACS Chem. Neurosci. 5 (9), 749–751. 10.1021/cn500171p 25111714

[B150] Kini R. M. (2006). Anticoagulant proteins from snake venoms: structure, function and mechanism. Biochem. J. 397 (3), 377–387. 10.1042/BJ20060302 16831131 PMC1533313

[B151] Kini R. M. Koh C. Y. (2020). Snake venom three-finger toxins and their potential in drug development targeting cardiovascular diseases. Biochem. Pharmacol. 181, 114105. 10.1016/j.bcp.2020.114105 32579959

[B152] Klaiss-Luna M. C. Giraldo-Lorza J. M. Jemiola-Rzeminska M. Strzalka K. Manrique-Moreno M. (2023). Biophysical insights into the antitumoral activity of crotalicidin against breast cancer model membranes. Int. J. Mol. Sci. 24 (22), 16226. 10.3390/ijms242216226 38003414 PMC10671781

[B153] Kohn A. J. (2018). Conus envenomation of humans: in fact and fiction. Toxins (Basel) 11 (1), 10. 10.3390/toxins11010010 30591658 PMC6356772

[B154] Koludarov I. Jackson T. N. Sunagar K. Nouwens A. Hendrikx I. Fry B. G. (2014). Fossilized venom: the unusually conserved venom profiles of Heloderma species (beaded lizards and gila monsters). Toxins (Basel) 6 (12), 3582–3595. 10.3390/toxins6123582 25533521 PMC4280549

[B155] Kozlov S. A. Vassilevski A. A. Feofanov A. V. Surovoy A. Y. Karpunin D. V. Grishin E. V. (2006). Latarcins, antimicrobial and cytolytic peptides from the venom of the spider Lachesana tarabaevi (Zodariidae) that exemplify biomolecular diversity. J. Biol. Chem. 281 (30), 20983–20992. 10.1074/jbc.M602168200 16735513

[B156] Kutzsche J. Guzman G. A. Willuweit A. Kletke O. Wollert E. Gering I. (2024). An orally available Ca(v)2.2 calcium channel inhibitor for the treatment of neuropathic pain. Br. J. Pharmacol. 181 (12), 1734–1756. 10.1111/bph.16309 38157867

[B157] Kvetkina A. Malyarenko O. Pavlenko A. Dyshlovoy S. von Amsberg G. Ermakova S. (2020). Sea anemone heteractis crispa actinoporin demonstrates *in vitro* anticancer activities and prevents HT-29 colorectal cancer cell migration. Molecules 25 (24), 5979. 10.3390/molecules25245979 33348592 PMC7766076

[B158] Langenegger N. Nentwig W. Kuhn-Nentwig L. (2019). Spider venom: components, modes of action, and novel strategies in transcriptomic and proteomic analyses. Toxins (Basel) 11 (10), 611. 10.3390/toxins11100611 31652611 PMC6832493

[B159] Lebbe E. K. Peigneur S. Wijesekara I. Tytgat J. (2014). Conotoxins targeting nicotinic acetylcholine receptors: an overview. Mar. Drugs 12 (5), 2970–3004. 10.3390/md12052970 24857959 PMC4052327

[B160] Lebbe E. K. Tytgat J. (2016). In the picture: disulfide-poor conopeptides, a class of pharmacologically interesting compounds. J. Venom. Anim. Toxins Incl. Trop. Dis. 22, 30. 10.1186/s40409-016-0083-6 27826319 PMC5100318

[B161] Lebreton L. Tuffigo M. Pillois X. Fiore M. (2016). L’intégrine α_IIb_β_3_: Une actrice insoupçonnée dans la formation des plaquettes sanguines. Med. Sci. Paris. 32 (3), 290–296. 10.1051/medsci/20163203014 27011248

[B162] Lee B. Shin M. K. Hwang I. W. Jung J. Shim Y. J. Kim G. W. (2021). A deep learning approach with data augmentation to predict novel spider neurotoxic peptides. Int. J. Mol. Sci. 22 (22), 12291. 10.3390/ijms222212291 34830173 PMC8619404

[B163] Lee C. J. Ansell J. E. (2011). Direct thrombin inhibitors. Br. J. Clin. Pharmacol. 72 (4), 581–592. 10.1111/j.1365-2125.2011.03916.x 21241354 PMC3195735

[B164] Li F. Wu S. Chen N. Zhu J. Zhao X. Zhang P. (2021). Fatty acid modification of the anticancer peptide LVTX-9 to enhance its cytotoxicity against malignant melanoma cells. Toxins (Basel) 13 (12), 867. 10.3390/toxins13120867 34941705 PMC8708390

[B165] Li T. Zhao X. M. Li L. (2022). Co-VAE: drug-target binding affinity prediction by Co-regularized variational Autoencoders. IEEE Trans. Pattern Anal. Mach. Intell. 44 (12), 8861–8873. 10.1109/TPAMI.2021.3120428 34652996

[B166] Liao Q. Feng Y. Yang B. Lee S. M. (2019). Cnidarian peptide neurotoxins: a new source of various ion channel modulators or blockers against central nervous systems disease. Drug Discov. Today 24 (1), 189–197. 10.1016/j.drudis.2018.08.011 30165198

[B167] Ligabue-Braun R. Verli H. Carlini C. R. (2012). Venomous mammals: a review. Toxicon 59 (7-8), 680–695. 10.1016/j.toxicon.2012.02.012 22410495

[B168] Limam I. Abdelkarim M. El Ayeb M. Crepin M. Marrakchi N. Di Benedetto M. (2023). Disintegrin-like protein strategy to inhibit aggressive triple-negative breast cancer. Int. J. Mol. Sci. 24 (15), 12219. 10.3390/ijms241512219 37569595 PMC10418936

[B169] Lin E. Wang Q. Swenson S. Jadvar H. Groshen S. Ye W. (2010). The disintegrin contortrostatin in combination with docetaxel is a potent inhibitor of prostate cancer *in vitro* and *in vivo* . Prostate 70 (12), 1359–1370. 10.1002/pros.21173 20623636

[B170] Liu Y. Ming W. Wang Y. Liu S. Qiu Y. Xiang Y. (2019). Cytotoxin 1 from Naja atra Cantor venom induced necroptosis of leukemia cells. Toxicon 165, 110–115. 10.1016/j.toxicon.2019.04.012 31029638

[B171] Liu Z. Deng M. Xiang J. Ma H. Hu W. Zhao Y. (2012). A novel spider peptide toxin suppresses tumor growth through dual signaling pathways. Curr. Mol. Med. 12 (10), 1350–1360. 10.2174/156652412803833643 22882120

[B172] Lluisma A. O. Lopez-Vera E. Bulaj G. Watkins M. Olivera B. M. (2008). Characterization of a novel psi-conotoxin from Conus parius Reeve. Toxicon 51 (2), 174–180. 10.1016/j.toxicon.2007.07.009 18054976 PMC2669105

[B173] Lucena S. E. Jia Y. Soto J. G. Parral J. Cantu E. Brannon J. (2012). Anti-invasive and anti-adhesive activities of a recombinant disintegrin, r-viridistatin 2, derived from the Prairie rattlesnake (Crotalus viridis viridis). Toxicon 60 (1), 31–39. 10.1016/j.toxicon.2012.03.011 22465495 PMC3874882

[B174] Luna-Ramirez K. Quintero-Hernandez V. Vargas-Jaimes L. Batista C. V. Winkel K. D. Possani L. D. (2013). Characterization of the venom from the Australian scorpion Urodacus yaschenkoi: molecular mass analysis of components, cDNA sequences and peptides with antimicrobial activity. Toxicon 63, 44–54. 10.1016/j.toxicon.2012.11.017 23182832

[B175] Luo Q. Wu T. Wu W. Chen G. Luo X. Jiang L. (2020). The functional role of voltage-gated sodium channel Nav1.5 in metastatic breast cancer. Front. Pharmacol. 11, 1111. 10.3389/fphar.2020.01111 32792949 PMC7393602

[B176] Mamelak A. N. Rosenfeld S. Bucholz R. Raubitschek A. Nabors L. B. Fiveash J. B. (2006). Phase I single-dose study of intracavitary-administered iodine-131-TM-601 in adults with recurrent high-grade glioma. J. Clin. Oncol. 24 (22), 3644–3650. 10.1200/JCO.2005.05.4569 16877732

[B177] Margiotta F. Micheli L. Ciampi C. Ghelardini C. McIntosh J. M. Di Cesare Mannelli L. (2022). Conus regius-derived conotoxins: novel therapeutic opportunities from a marine organism. Mar. Drugs 20 (12), 773. 10.3390/md20120773 36547920 PMC9783627

[B178] Martinez-Hernandez L. Lopez-Vera E. Aguilar M. B. Rodriguez-Ruiz X. C. Ortiz-Arellano M. A. (2023). κO-SrVIA conopeptide, a novel inhibitor peptide for two members of the human EAG potassium channel family. Int. J. Mol. Sci. 24 (14), 11513. 10.3390/ijms241411513 37511269 PMC10380377

[B179] Matteson D. R. Deutsch C. (1984). K channels in T lymphocytes: a patch clamp study using monoclonal antibody adhesion. Nature 307 (5950), 468–471. 10.1038/307468a0 6320008

[B180] McDowell R. S. Dennis M. S. Louie A. Shuster M. Mulkerrin M. G. Lazarus R. A. (1992). Mambin, a potent glycoprotein IIb-IIIa antagonist and platelet aggregation inhibitor structurally related to the short neurotoxins. Biochemistry 31 (20), 4766–4772. 10.1021/bi00135a004 1591238

[B181] McIntosh M. Cruz L. J. Hunkapiller M. W. Gray W. R. Olivera B. M. (1982). Isolation and structure of a peptide toxin from the marine snail Conus magus. Arch. Biochem. Biophys. 218 (1), 329–334. 10.1016/0003-9861(82)90351-4 7149738

[B182] Mendes L. C. Viana G. M. M. Nencioni A. L. A. Pimenta D. C. Beraldo-Neto E. (2023). Scorpion peptides and ion channels: an insightful review of mechanisms and drug development. Toxins (Basel) 15 (4), 238. 10.3390/toxins15040238 37104176 PMC10145618

[B183] Miljanich G. P. (2004). Ziconotide: neuronal calcium channel blocker for treating severe chronic pain. Curr. Med. Chem. 11 (23), 3029–3040. 10.2174/0929867043363884 15578997

[B184] Minea R. O. Helchowski C. M. Zidovetzki S. J. Costa F. K. Swenson S. D. Markland F. S. Jr. (2010). Vicrostatin - an anti-invasive multi-integrin targeting chimeric disintegrin with tumor anti-angiogenic and pro-apoptotic activities. PLoS One 5 (6), e10929. 10.1371/journal.pone.0010929 20532165 PMC2880590

[B185] Mlayah-Bellalouna S. Aissaoui-Zid D. Chantome A. Jebali J. Souid S. Ayedi E. (2023). Insights into the mechanisms governing P01 scorpion toxin effect against U87 glioblastoma cells oncogenesis. Front. Pharmacol. 14, 1203247. 10.3389/fphar.2023.1203247 37426811 PMC10326281

[B186] Mo Y. Shi Q. Qi G. Chen K. (2023). Potential anti-tumor effects of *Solenopsis invicta* venom. Front. Immunol. 14, 1200659. 10.3389/fimmu.2023.1200659 37283754 PMC10239855

[B187] Mohan M. K. Abraham N. R P. R. Jayaseelan B. F. Ragnarsson L. Lewis R. J. (2020). Structure and allosteric activity of a single-disulfide conopeptide from Conus zonatus at human alpha3beta4 and alpha7 nicotinic acetylcholine receptors. J. Biol. Chem. 295 (20), 7096–7112. 10.1074/jbc.RA119.012098 32234761 PMC7242716

[B188] Morales Duque H. Campos Dias S. Franco O. L. (2019). Structural and functional analyses of cone snail toxins. Mar. Drugs 17 (6), 370. 10.3390/md17060370 31234371 PMC6628382

[B189] Moreels L. Peigneur S. Yamaguchi Y. Vriens K. Waelkens E. Zhu S. (2017). Expanding the pharmacological profile of kappa-hefutoxin 1 and analogues: a focus on the inhibitory effect on the oncogenic channel K(v)10.1. Peptides 98, 43–50. 10.1016/j.peptides.2016.08.008 27578329

[B190] Morjen M. Kallech-Ziri O. Bazaa A. Othman H. Mabrouk K. Zouari-Kessentini R. (2013). PIVL, a new serine protease inhibitor from *Macrovipera lebetina* transmediterranea venom, impairs motility of human glioblastoma cells. Matrix Biol. 32 (1), 52–62. 10.1016/j.matbio.2012.11.015 23262217

[B191] Morsy M. A. Gupta S. Dora C. P. Jhawat V. Dhanawat M. Mehta D. (2023). Venoms classification and therapeutic uses: a narrative review. Eur. Rev. Med. Pharmacol. Sci. 27 (4), 1633–1653. 10.26355/eurrev_202302_31408 36876699

[B192] Mouhat S. Visan V. Ananthakrishnan S. Wulff H. Andreotti N. Grissmer S. (2005). K+ channel types targeted by synthetic OSK1, a toxin from Orthochirus scrobiculosus scorpion venom. Biochem. J. 385 (Pt 1), 95–104. 10.1042/BJ20041379 15588251 PMC1134677

[B193] Muller S. P. Silva V. A. O. Silvestrini A. V. P. de Macedo L. H. Caetano G. F. Reis R. M. (2018). Crotoxin from *Crotalus durissus* terrificus venom: *in vitro* cytotoxic activity of a heterodimeric phospholipase A(2) on human cancer-derived cell lines. Toxicon 156, 13–22. 10.1016/j.toxicon.2018.10.306 30395843

[B194] Munawar A. Ali S. A. Akrem A. Betzel C. (2018). Snake venom peptides: tools of biodiscovery. Toxins (Basel) 10 (11), 474. 10.3390/toxins10110474 30441876 PMC6266942

[B195] Munhoz J. Thome R. Rostami A. Ishikawa L. L. W. Verinaud L. Raposo C. (2021). The SNX-482 peptide from Hysterocrates gigas spider acts as an immunomodulatory molecule activating macrophages. Peptides 146, 170648. 10.1016/j.peptides.2021.170648 34537257

[B196] Murray C. J. Rosenfeld L. C. Lim S. S. Andrews K. G. Foreman K. J. Haring D. (2012). Global malaria mortality between 1980 and 2010: a systematic analysis. Lancet 379 (9814), 413–431. 10.1016/S0140-6736(12)60034-8 22305225

[B197] Nadkarni P. Chepurny O. G. Holz G. G. (2014). Regulation of glucose homeostasis by GLP-1. Prog. Mol. Biol. Transl. Sci. 121, 23–65. 10.1016/B978-0-12-800101-1.00002-8 24373234 PMC4159612

[B198] Nascimento F. D. Sancey L. Pereira A. Rome C. Oliveira V. Oliveira E. B. (2012). The natural cell-penetrating peptide crotamine targets tumor tissue *in vivo* and triggers a lethal calcium-dependent pathway in cultured cells. Mol. Pharm. 9 (2), 211–221. 10.1021/mp2000605 22142367

[B199] Nauck M. A. Quast D. R. Wefers J. Meier J. J. (2021). GLP-1 receptor agonists in the treatment of type 2 diabetes - state-of-the-art. Mol. Metab. 46, 101102. 10.1016/j.molmet.2020.101102 33068776 PMC8085572

[B200] Neff R. A. Wickenden A. D. (2021). Selective targeting of Nav1.7 with engineered spider venom-based peptides. Channels (Austin) 15 (1), 193–207. 10.1080/19336950.2020.1860382 PMC7808416 33427574

[B201] Newcomb R. Szoke B. Palma A. Wang G. Chen X. Hopkins W. (1998). Selective peptide antagonist of the class E calcium channel from the venom of the tarantula Hysterocrates gigas. Biochemistry 37 (44), 15353–15362. 10.1021/bi981255g 9799496

[B202] Nguyen P. T. Nguyen H. M. Wagner K. M. Stewart R. G. Singh V. Thapa P. (2022a). Computational design of peptides to target Na(V)1.7 channel with high potency and selectivity for the treatment of pain. Elife 11, e81727. 10.7554/eLife.81727 36576241 PMC9831606

[B203] Nguyen T. Guo R. Chai J. Wu J. Liu J. Chen X. (2022b). Smp24, a scorpion-venom peptide, exhibits potent antitumor effects against hepatoma HepG2 cells via multi-mechanisms *in vivo* and *in vitro* . Toxins (Basel) 14 (10), 717. 10.3390/toxins14100717 36287985 PMC9607800

[B204] Nielsen C. K. Lewis R. J. Alewood D. Drinkwater R. Palant E. Patterson M. (2005). Anti-allodynic efficacy of the chi-conopeptide, Xen2174, in rats with neuropathic pain. Pain 118 (1-2), 112–124. 10.1016/j.pain.2005.08.002 16154696

[B205] Nimmrich V. Gross G. (2012). P/Q-type calcium channel modulators. Br. J. Pharmacol. 167 (4), 741–759. 10.1111/j.1476-5381.2012.02069.x 22670568 PMC3575775

[B206] Okada M. Corzo G. Romero-Perez G. A. Coronas F. Matsuda H. Possani L. D. (2015). A pore forming peptide from spider Lachesana sp. venom induced neuronal depolarization and pain. Biochim. Biophys. Acta 1850 (4), 657–666. 10.1016/j.bbagen.2014.11.022 25484315

[B207] Okada M. Ortiz E. Corzo G. Possani L. D. (2019). Pore-forming spider venom peptides show cytotoxicity to hyperpolarized cancer cells expressing K+ channels: a lentiviral vector approach. PLoS One 14 (4), e0215391. 10.1371/journal.pone.0215391 30978253 PMC6461346

[B208] Okkerse P. Hay J. L. Sitsen E. Dahan A. Klaassen E. Houghton W. (2017). Pharmacokinetics and pharmacodynamics of intrathecally administered Xen2174, a synthetic conopeptide with norepinephrine reuptake inhibitor and analgesic properties. Br. J. Clin. Pharmacol. 83 (4), 751–763. 10.1111/bcp.13176 27987228 PMC5346871

[B209] Olaoba O. T. Karina Dos Santos P. Selistre-de-Araujo H. S. Ferreira de Souza D. H. (2020). Snake venom metalloproteinases (SVMPs): a structure-function update. Toxicon X 7, 100052. 10.1016/j.toxcx.2020.100052 32776002 PMC7399193

[B210] Oliveira A. L. Viegas M. F. da Silva S. L. Soares A. M. Ramos M. J. Fernandes P. A. (2022). The chemistry of snake venom and its medicinal potential. Nat. Rev. Chem. 6 (7), 451–469. 10.1038/s41570-022-00393-7 PMC9185726 35702592

[B211] Oliveira-Mendes B. B. R. Horta C. C. R. do Carmo A. O. Biscoto G. L. Sales-Medina D. F. Leal H. G. (2018). CPP-Ts: a new intracellular calcium channel modulator and a promising tool for drug delivery in cancer cells. Sci. Rep. 8 (1), 14739. 10.1038/s41598-018-33133-3 30282983 PMC6170434

[B212] Olsen C. Tarcha E. Probst P. Peckham D. Iadonato S. (2016). LB779 Dalazatide (ShK-186), a first-in-class peptide inhibitor of Kv1.3 potassium channels, demonstrates safety, tolerability and proof of concept of efficacy in patients with active plaque psoriasis. J. Investigative Dermatology 136 (8), B5. 10.1016/j.jid.2016.05.029

[B213] Ortiz E. Gurrola G. B. Schwartz E. F. Possani L. D. (2015). Scorpion venom components as potential candidates for drug development. Toxicon 93, 125–135. 10.1016/j.toxicon.2014.11.233 25432067 PMC7130864

[B214] Osteen J. D. Herzig V. Gilchrist J. Emrick J. J. Zhang C. Wang X. (2016). Selective spider toxins reveal a role for the Nav1.1 channel in mechanical pain. Nature 534 (7608), 494–499. 10.1038/nature17976 27281198 PMC4919188

[B215] Ownby C. L. Fletcher J. E. Colberg T. R. (1993). Cardiotoxin 1 from cobra (Naja naja atra) venom causes necrosis of skeletal muscle *in vivo* . Toxicon 31 (6), 697–709. 10.1016/0041-0101(93)90376-t 8342169

[B216] Pandey P. Khan F. Khan M. A. Kumar R. Upadhyay T. K. (2023). An updated review summarizing the anticancer efficacy of melittin from bee venom in several models of human cancers. Nutrients 15 (14), 3111. 10.3390/nu15143111 37513529 PMC10385528

[B217] Pardo L. A. del Camino D. Sanchez A. Alves F. Bruggemann A. Beckh S. (1999). Oncogenic potential of EAG K(+) channels. EMBO J. 18 (20), 5540–5547. 10.1093/emboj/18.20.5540 10523298 PMC1171622

[B218] Patchett A. A. (1984). The chemistry of enalapril. Br. J. Clin. Pharmacol. 18 (Suppl. 2), 201S–207S. 10.1111/j.1365-2125.1984.tb02599.x 6085275 PMC1463492

[B219] Patil C. G. Walker D. G. Miller D. M. Butte P. Morrison B. Kittle D. S. (2019). Phase 1 safety, pharmacokinetics, and fluorescence imaging study of tozuleristide (BLZ-100) in adults with newly diagnosed or recurrent gliomas. Neurosurgery 85 (4), E641–E649. 10.1093/neuros/nyz125 31069381

[B220] Pedron C. Antunes F. T. T. Rebelo I. N. Campos M. M. Correa A. P. Klein C. P. (2021). Phoneutria nigriventer Tx3-3 peptide toxin reduces fibromyalgia symptoms in mice. Neuropeptides 85, 102094. 10.1016/j.npep.2020.102094 33171335

[B221] Peigneur S. de Lima M. E. Tytgat J. (2018). Phoneutria nigriventer venom: a pharmacological treasure. Toxicon 151, 96–110. 10.1016/j.toxicon.2018.07.008 30003916

[B222] Peigneur S. Devi P. Seldeslachts A. Ravichandran S. Quinton L. Tytgat J. (2019). Structure-function elucidation of a new α-conotoxin, MilIA, from Conus milneedwardsi. Mar. Drugs 17 (9), 535. 10.3390/md17090535 31527432 PMC6780063

[B223] Pereira A. Kerkis A. Hayashi M. A. Pereira A. S. Silva F. S. Oliveira E. B. (2011). Crotamine toxicity and efficacy in mouse models of melanoma. Expert Opin. Investig. Drugs 20 (9), 1189–1200. 10.1517/13543784.2011.602064 21834748

[B224] Perez-Peinado C. Valle J. Freire J. M. Andreu D. (2020). Tumor cell attack by crotalicidin (ctn) and its fragment ctn[15-34]: insights into their dual membranolytic and intracellular targeting mechanism. ACS Chem. Biol. 15 (11), 2945–2957. 10.1021/acschembio.0c00596 33021779

[B225] Phillips A. J. Govedich F. R. Moser W. E. (2020). Leeches in the extreme: morphological, physiological, and behavioral adaptations to inhospitable habitats. Int. J. Parasitol. Parasites Wildl. 12, 318–325. 10.1016/j.ijppaw.2020.09.003 33101909 PMC7569739

[B226] Phuong H. B. T. Tran V. A. Ngoc K. N. Huu V. N. Thu H. N. Van M. C. (2023). Effect of substituting glutamine with lysine on structural and biological properties of antimicrobial peptide Polybia-MP1. Amino Acids 55 (7), 881–890. 10.1007/s00726-023-03276-3 37300579

[B227] Ponce-Soto L. A. Lomonte B. Gutierrez J. M. Rodrigues-Simioni L. Novello J. C. Marangoni S. (2007). Structural and functional properties of BaTX, a new Lys49 phospholipase A2 homologue isolated from the venom of the snake Bothrops alternatus. Biochim. Biophys. Acta 1770 (4), 585–593. 10.1016/j.bbagen.2006.11.015 17270350

[B228] Potter L. R. Yoder A. R. Flora D. R. Antos L. K. Dickey D. M. (2009). Natriuretic peptides: their structures, receptors, physiologic functions and therapeutic applications. Handb. Exp. Pharmacol. 191, 341–366. 10.1007/978-3-540-68964-5_15 PMC4855512 19089336

[B229] Prieto A. R. Ma H. Huang R. Khan G. Schwartz K. A. Hage-Korban E. E. (2002). Thrombostatin, a bradykinin metabolite, reduces platelet activation in a model of arterial wall injury. Cardiovasc Res. 53 (4), 984–992. 10.1016/s0008-6363(01)00514-4 11922908

[B230] Qi J. Wang W. Lu W. Chen W. Sun H. Shang A. (2020). Design and biological evaluation of novel BF-30 analogs for the treatment of malignant melanoma. J. Cancer 11 (24), 7184–7195. 10.7150/jca.47549 33193881 PMC7646182

[B231] Rashid M. H. Huq R. Tanner M. R. Chhabra S. Khoo K. K. Estrada R. (2014). A potent and Kv1.3-selective analogue of the scorpion toxin HsTX1 as a potential therapeutic for autoimmune diseases. Sci. Rep. 4, 4509. 10.1038/srep04509 24676092 PMC3968461

[B232] Ratibou Z. Inguimbert N. Dutertre S. (2024). Predatory and defensive strategies in cone snails. Toxins (Basel) 16 (2), 94. 10.3390/toxins16020094 38393171 PMC10892987

[B233] Reis P. V. M. Boff D. Verly R. M. Melo-Braga M. N. Cortes M. E. Santos D. M. (2018). LyeTxI-b, a synthetic peptide derived from Lycosa erythrognatha spider venom, shows potent antibiotic activity *in vitro* and *in vivo* . Front. Microbiol. 9, 667. 10.3389/fmicb.2018.00667 29681894 PMC5897548

[B234] Ren C. Li Y. Cong Z. Li Z. Xie L. Wu S. (2023). Bioengineered bacterial outer membrane vesicles encapsulated Polybia-mastoparan I fusion peptide as a promising nanoplatform for bladder cancer immune-modulatory chemotherapy. Front. Immunol. 14, 1129771. 10.3389/fimmu.2023.1129771 36999028 PMC10043419

[B235] Richards K. L. Milligan C. J. Richardson R. J. Jancovski N. Grunnet M. Jacobson L. H. (2018). Selective Na(V)1.1 activation rescues Dravet syndrome mice from seizures and premature death. Proc. Natl. Acad. Sci. U. S. A. 115 (34), E8077–E8085. 10.1073/pnas.1804764115 30076230 PMC6112713

[B236] Rigo F. K. Trevisan G. Rosa F. Dalmolin G. D. Otuki M. F. Cueto A. P. (2013). Spider peptide Phα1β induces analgesic effect in a model of cancer pain. Cancer Sci. 104 (9), 1226–1230. 10.1111/cas.12209 23718272 PMC7657190

[B237] Robinson S. D. Norton R. S. (2014). Conotoxin gene superfamilies. Mar. Drugs 12 (12), 6058–6101. 10.3390/md12126058 25522317 PMC4278219

[B238] Rooj A. K. McNicholas C. M. Bartoszewski R. Bebok Z. Benos D. J. Fuller C. M. (2012). Glioma-specific cation conductance regulates migration and cell cycle progression. J. Biol. Chem. 287 (6), 4053–4065. 10.1074/jbc.M111.311688 22130665 PMC3281704

[B239] Rosso J. P. Schwarz J. R. Diaz-Bustamante M. Ceard B. Gutierrez J. M. Kneussel M. (2015). MmTX1 and MmTX2 from coral snake venom potently modulate GABAA receptor activity. Proc. Natl. Acad. Sci. U. S. A. 112 (8), E891–E900. 10.1073/pnas.1415488112 25675485 PMC4345566

[B240] Russell F. E. Bogert C. M. (1981). Gila monster: its biology, venom and bite--a review. Toxicon 19 (3), 341–359. 10.1016/0041-0101(81)90040-4 7018022

[B241] Sachs F. (2015). Mechanical transduction by ion channels: a cautionary tale. World J. Neurol. 5 (3), 74–87. 10.5316/wjn.v5.i3.74 28078202 PMC5221657

[B242] Sadat S. N. Bagheri K. P. Maghsoudi H. Shahbazzadeh D. (2023). Oxineur, a novel peptide from Caspian cobra Naja naja oxiana against HT-29 colon cancer. Biochim. Biophys. Acta Gen. Subj. 1867 (2), 130285. 10.1016/j.bbagen.2022.130285 36462597

[B243] Saez N. J. Herzig V. (2019). Versatile spider venom peptides and their medical and agricultural applications. Toxicon 158, 109–126. 10.1016/j.toxicon.2018.11.298 30543821

[B244] Safavi-Hemami H. Gajewiak J. Karanth S. Robinson S. D. Ueberheide B. Douglass A. D. (2015). Specialized insulin is used for chemical warfare by fish-hunting cone snails. Proc. Natl. Acad. Sci. U. S. A. 112 (6), 1743–1748. 10.1073/pnas.1423857112 25605914 PMC4330763

[B245] Sandall D. W. Satkunanathan N. Keays D. A. Polidano M. A. Liping X. Pham V. (2003). A novel α-conotoxin identified by gene sequencing is active in suppressing the vascular response to selective stimulation of sensory nerves *in vivo* . Biochemistry 42 (22), 6904–6911. 10.1021/bi034043e 12779345

[B246] Sang C. N. Barnabe K. J. Kern S. E. (2016). Phase ia clinical trial evaluating the tolerability, pharmacokinetics, and analgesic efficacy of an intrathecally administered neurotensin A analogue in central neuropathic pain following spinal cord injury. Clin. Pharmacol. Drug Dev. 5 (4), 250–258. 10.1002/cpdd.253 27310326

[B247] Sanggaard K. W. Dyrlund T. F. Thomsen L. R. Nielsen T. A. Brondum L. Wang T. (2015). Characterization of the gila monster (*Heloderma suspectum* suspectum) venom proteome. J. Proteomics 117, 1–11. 10.1016/j.jprot.2015.01.004 25603280

[B248] Santos D. M. Verly R. M. Pilo-Veloso D. de Maria M. de Carvalho M. A. Cisalpino P. S. (2010). LyeTx I, a potent antimicrobial peptide from the venom of the spider Lycosa erythrognatha. Amino Acids 39 (1), 135–144. 10.1007/s00726-009-0385-x 19946788

[B249] Saverioni D. Notari S. Capellari S. Poggiolini I. Giese A. Kretzschmar H. A. (2013). Analyses of protease resistance and aggregation state of abnormal prion protein across the spectrum of human prions. J. Biol. Chem. 288 (39), 27972–27985. 10.1074/jbc.M113.477547 23897825 PMC3784711

[B250] Schendel V. Rash L. D. Jenner R. A. Undheim E. A. B. (2019). The diversity of venom: the importance of behavior and venom system morphology in understanding its ecology and evolution. Toxins (Basel) 11 (11), 666. 10.3390/toxins11110666 31739590 PMC6891279

[B251] Schonthal A. H. Swenson S. D. Chen T. C. Markland F. S. (2020). Preclinical studies of a novel snake venom-derived recombinant disintegrin with antitumor activity: a review. Biochem. Pharmacol. 181, 114149. 10.1016/j.bcp.2020.114149 32663453

[B252] Schwartz E. F. Capes E. M. Diego-Garcia E. Zamudio F. Z. Fuentes O. Possani L. D. (2009). Characterization of hadrucalcin, a peptide from Hadrurus gertschi scorpion venom with pharmacological activity on ryanodine receptors. Br. J. Pharmacol. 157 (3), 392–403. 10.1111/j.1476-5381.2009.00147.x 19389159 PMC2707986

[B253] Seo S. Choi J. Park S. Ahn J. (2021). Binding affinity prediction for protein-ligand complex using deep attention mechanism based on intermolecular interactions. BMC Bioinforma. 22 (1), 542. 10.1186/s12859-021-04466-0 PMC8576937 34749664

[B254] Serrano S. M. Maroun R. C. (2005). Snake venom serine proteinases: sequence homology vs. substrate specificity, a paradox to be solved. Toxicon 45 (8), 1115–1132. 10.1016/j.toxicon.2005.02.020 15922778

[B255] Servent D. Blanchet G. Mourier G. Marquer C. Marcon E. Fruchart-Gaillard C. (2011). Muscarinic toxins. Toxicon 58 (6-7), 455–463. 10.1016/j.toxicon.2011.08.004 21906611

[B256] Shahbazzadeh D. Srairi-Abid N. Feng W. Ram N. Borchani L. Ronjat M. (2007). Hemicalcin, a new toxin from the Iranian scorpion Hemiscorpius lepturus which is active on ryanodine-sensitive Ca2+ channels. Biochem. J. 404 (1), 89–96. 10.1042/BJ20061404 17291197 PMC1868827

[B257] Shen H. Xie Y. Ye S. He K. Yi L. Cui R. (2018). Spider peptide toxin lycosin-I induces apoptosis and inhibits migration of prostate cancer cells. Exp. Biol. Med. (Maywood) 243 (8), 725–735. 10.1177/1535370218772802 29763387 PMC6378508

[B258] Shiu J. H. Chen C. Y. Chang L. S. Chen Y. C. Chen Y. C. Lo Y. H. (2004). Solution structure of gamma-bungarotoxin: the functional significance of amino acid residues flanking the RGD motif in integrin binding. Proteins 57 (4), 839–849. 10.1002/prot.20269 15390258

[B259] Sieghart W. (2006). Structure, pharmacology, and function of GABAA receptor subtypes. Adv. Pharmacol. 54, 231–263. 10.1016/s1054-3589(06)54010-4 17175817

[B260] Silva F. R. Batista E. M. Gomez M. V. Kushmerick C. Da Silva J. F. Cordeiro M. N. (2016). The Phoneutria nigriventer spider toxin, PnTx4-5-5, promotes neuronal survival by blocking NMDA receptors. Toxicon official J. Int. Soc. Toxinology 112, 16–21. 10.1016/j.toxicon.2016.01.056 26802625

[B261] Simoes-Silva R. Alfonso J. Gomez A. Holanda R. J. Sobrinho J. C. Zaqueo K. D. (2018). Snake venom, A natural library of new potential therapeutic molecules: challenges and current perspectives. Curr. Pharm. Biotechnol. 19 (4), 308–335. 10.2174/1389201019666180620111025 29929461

[B262] Smith J. J. Lau C. H. Y. Herzig V. Ikonomopoulou M. P. Rash L. D. King G. F. (2015). “Therapeutic applications of spider-venom peptides,” in Venoms to drugs: therapeutic applications of spider-venom peptides, 221–244.

[B263] Smith J. J. Vetter I. Lewis R. J. Peigneur S. Tytgat J. Lam A. (2013). Multiple actions of phi-LITX-Lw1a on ryanodine receptors reveal a functional link between scorpion DDH and ICK toxins. Proc. Natl. Acad. Sci. U. S. A. 110 (22), 8906–8911. 10.1073/pnas.1214062110 23671114 PMC3670328

[B264] Soares S. Lopes K. S. Mortari M. Oliveira H. Bastos V. (2022). Antitumoral potential of Chartergellus-CP1 peptide from Chartergellus communis wasp venom in two different breast cancer cell lines (HR+ and triple-negative). Toxicon 216, 148–156. 10.1016/j.toxicon.2022.07.004 35839869

[B265] Soltan-Alinejad P. Alipour H. Meharabani D. Azizi K. (2022). Therapeutic potential of bee and scorpion venom phospholipase A2 (PLA2): a narrative review. Iran. J. Med. Sci. 47 (4), 300–313. 10.30476/IJMS.2021.88511.1927 35919080 PMC9339116

[B266] Son D. J. Lee J. W. Lee Y. H. Song H. S. Lee C. K. Hong J. T. (2007). Therapeutic application of anti-arthritis, pain-releasing, and anti-cancer effects of bee venom and its constituent compounds. Pharmacol. Ther. 115 (2), 246–270. 10.1016/j.pharmthera.2007.04.004 17555825

[B267] Sousa S. R. Vetter I. Lewis R. J. (2013). Venom peptides as a rich source of cav2.2 channel blockers. Toxins (Basel) 5 (2), 286–314. 10.3390/toxins5020286 23381143 PMC3640536

[B268] Souza B. M. Mendes M. A. Santos L. D. Marques M. R. Cesar L. M. Almeida R. N. (2005). Structural and functional characterization of two novel peptide toxins isolated from the venom of the social wasp Polybia paulista. Peptides 26 (11), 2157–2164. 10.1016/j.peptides.2005.04.026 16129513

[B269] Souza I. A. Cino E. A. Choy W. Y. Cordeiro M. N. Richardson M. Chavez-Olortegui C. (2012). Expression of a recombinant Phoneutria toxin active in calcium channels. Toxicon official J. Int. Soc. Toxinology 60 (5), 907–918. 10.1016/j.toxicon.2012.05.026 22659539

[B270] Stepensky D. (2018). Pharmacokinetics of toxin-derived peptide drugs. Toxins (Basel) 10 (11), 483. 10.3390/toxins10110483 30463321 PMC6266565

[B271] Stewart J. M. (2018). Peptide composition for cancer treatment by inhibiting TRPV6 calcium channel activity United States of America patent application 15/088,993.

[B272] Stewart J. M. Steeves B. J. Vernes K. (2006). Paralytic peptide for use in neuromuscular therapy United States of America patent application 10/858,233.

[B273] Sudarikova A. V. Bychkov M. L. Kulbatskii D. S. Chubinskiy-Nadezhdin V. I. Shlepova O. V. Shulepko M. A. (2022). Mambalgin-2 inhibits lung adenocarcinoma growth and migration by selective interaction with ASIC1/α-ENaC/γ-ENaC heterotrimer. Front. Oncol. 12, 904742. 10.3389/fonc.2022.904742 35837090 PMC9273970

[B274] Swartz K. J. MacKinnon R. (1995). An inhibitor of the Kv2.1 potassium channel isolated from the venom of a Chilean tarantula. Neuron 15 (4), 941–949. 10.1016/0896-6273(95)90184-1 7576642

[B275] Swenson S. Costa F. Minea R. Sherwin R. P. Ernst W. Fujii G. (2004). Intravenous liposomal delivery of the snake venom disintegrin contortrostatin limits breast cancer progression. Mol. cancer Ther. 3 (4), 499–511. 10.1158/1535-7163.499.3.4 15078994

[B276] Swenson S. Minea R. O. Tuan C. D. Thein T. Z. Chen T. C. Markland F. S. (2018). A novel venom-derived peptide for brachytherapy of glioblastoma: preclinical studies in mice. Molecules 23 (11), 2918. 10.3390/molecules23112918 30413113 PMC6278533

[B277] Tadokoro T. Modahl C. M. Maenaka K. Aoki-Shioi N. (2020). Cysteine-rich secretory proteins (CRISPs) from venomous snakes: an overview of the functional diversity in A large and underappreciated superfamily. Toxins (Basel) 12 (3), 175. 10.3390/toxins12030175 32178374 PMC7150914

[B278] Tan H. Huang Y. Xu J. Chen B. Zhang P. Ye Z. (2017). Spider toxin peptide lycosin-I functionalized gold nanoparticles for *in vivo* tumor targeting and therapy. Theranostics 7 (12), 3168–3178. 10.7150/thno.19780 28839471 PMC5566113

[B279] Tan H. Liu S. He Y. Cheng G. Zhang Y. Wei X. (2021). Spider toxin peptide-induced NIR gold nanocluster fabrication for GSH-responsive cancer cell imaging and nuclei translocation. Front. Bioeng. Biotechnol. 9, 780223. 10.3389/fbioe.2021.780223 34869292 PMC8635238

[B280] Tan H. Luo W. Wei L. Chen B. Li W. Xiao L. (2016). Quantifying the distribution of the stoichiometric composition of anticancer peptide lycosin-I on the lipid membrane with single molecule spectroscopy. J. Phys. Chem. B 120 (12), 3081–3088. 10.1021/acs.jpcb.5b12618 26937786

[B281] Tarcha E. J. Olsen C. M. Probst P. Peckham D. Munoz-Elias E. J. Kruger J. G. (2017). Safety and pharmacodynamics of dalazatide, a Kv1.3 channel inhibitor, in the treatment of plaque psoriasis: a randomized phase 1b trial. PLoS One 12 (7), e0180762. 10.1371/journal.pone.0180762 28723914 PMC5516987

[B282] Tasoulis T. Isbister G. K. (2017). A review and database of snake venom proteomes. Toxins (Basel) 9 (9), 290. 10.3390/toxins9090290 28927001 PMC5618223

[B283] Tasoulis T. Isbister G. K. (2023). A current perspective on snake venom composition and constituent protein families. Arch. Toxicol. 97 (1), 133–153. 10.1007/s00204-022-03420-0 36437303

[B284] Teesalu T. Sugahara K. N. Kotamraju V. R. Ruoslahti E. (2009). C-end rule peptides mediate neuropilin-1-dependent cell, vascular, and tissue penetration. Proc. Natl. Acad. Sci. U. S. A. 106 (38), 16157–16162. 10.1073/pnas.0908201106 19805273 PMC2752543

[B285] Teoh S. Yap M. (2020). Naja sumatrana venom cytotoxin, suma CTX exhibits concentration-dependent cytotoxicity via caspase-activated mitochondrial-mediated apoptosis without transitioning to necrosis. Toxin Rev. 40, 886–900. 10.1080/15569543.2020.1799408

[B286] Terlau H. Olivera B. M. (2004). Conus venoms: a rich source of novel ion channel-targeted peptides. Physiol. Rev. 84 (1), 41–68. 10.1152/physrev.00020.2003 14715910

[B287] Tonello R. Rigo F. Gewehr C. Trevisan G. Pereira E. M. Gomez M. V. (2014). Action of Phα1β, a peptide from the venom of the spider Phoneutria nigriventer, on the analgesic and adverse effects caused by morphine in mice. J. Pain 15 (6), 619–631. 10.1016/j.jpain.2014.02.007 24607814

[B288] Tsend-Ayush E. He C. Myers M. A. Andrikopoulos S. Wong N. Sexton P. M. (2016). Monotreme glucagon-like peptide-1 in venom and gut: one gene - two very different functions. Sci. Rep. 6, 37744. 10.1038/srep37744 27898108 PMC5127184

[B289] Tuba Z. Maho S. Vizi E. S. (2002). Synthesis and structure-activity relationships of neuromuscular blocking agents. Curr. Med. Chem. 9 (16), 1507–1536. 10.2174/0929867023369466 12171561

[B290] Ullah A. (2020). Structure-function studies and mechanism of action of snake venom L-amino acid oxidases. Front. Pharmacol. 11, 110. 10.3389/fphar.2020.00110 32158389 PMC7052187

[B291] Urra F. A. Pulgar R. Gutierrez R. Hodar C. Cambiazo V. Labra A. (2015). Identification and molecular characterization of five putative toxins from the venom gland of the snake Philodryas chamissonis (Serpentes: dipsadidae). Toxicon 108, 19–31. 10.1016/j.toxicon.2015.09.032 26410112

[B292] Utkin Y. Vassilevski A. Kudryavtsev D. Undheim E. A. B. (2019). Editorial: animal toxins as comprehensive pharmacological tools to identify diverse ion channels. Front. Pharmacol. 10, 423. 10.3389/fphar.2019.00423 31068819 PMC6491772

[B293] Utkin Y. N. (2015). Animal venom studies: current benefits and future developments. World J. Biol. Chem. 6 (2), 28–33. 10.4331/wjbc.v6.i2.28 26009701 PMC4436903

[B294] Utkin Y. N. (2019). Last decade update for three-finger toxins: newly emerging structures and biological activities. World J. Biol. Chem. 10 (1), 17–27. 10.4331/wjbc.v10.i1.17 30622682 PMC6314878

[B295] Valdivia H. H. Kirby M. S. Lederer W. J. Coronado R. (1992). Scorpion toxins targeted against the sarcoplasmic reticulum Ca(2+)-release channel of skeletal and cardiac muscle. Proc. Natl. Acad. Sci. U. S. A. 89 (24), 12185–12189. 10.1073/pnas.89.24.12185 1334561 PMC50723

[B296] Vannini E. Mori E. Tantillo E. Schmidt G. Caleo M. Costa M. (2021). CTX-CNF1 recombinant protein selectively targets glioma cells *in vivo* . Toxins (Basel) 13 (3), 194. 10.3390/toxins13030194 33800135 PMC7998600

[B297] Vargas-Jaimes L. Xiao L. Zhang J. Possani L. D. Valdivia H. H. Quintero-Hernandez V. (2017). Recombinant expression of Intrepicalcin from the scorpion Vaejovis intrepidus and its effect on skeletal ryanodine receptors. Biochim. Biophys. Acta Gen. Subj. 1861 (4), 936–946. 10.1016/j.bbagen.2017.01.032 28159581 PMC5329131

[B298] Vasconcelos A. A. Estrada J. C. David V. Wermelinger L. S. Almeida F. C. L. Zingali R. B. (2021). Structure-function relationship of the disintegrin family: sequence signature and integrin interaction. Front. Mol. Biosci. 8, 783301. 10.3389/fmolb.2021.783301 34926583 PMC8678471

[B299] Veiseh M. Gabikian P. Bahrami S. B. Veiseh O. Zhang M. Hackman R. C. (2007). Tumor paint: a chlorotoxin:Cy5.5 bioconjugate for intraoperative visualization of cancer foci. Cancer Res. 67 (14), 6882–6888. 10.1158/0008-5472.CAN-06-3948 17638899

[B300] Vieira L. B. Kushmerick C. Hildebrand M. E. Garcia E. Stea A. Cordeiro M. N. (2005). Inhibition of high voltage-activated calcium channels by spider toxin PnTx3-6. J. Pharmacol. Exp. Ther. 314 (3), 1370–1377. 10.1124/jpet.105.087023 15933156

[B301] Vines J. B. Yoon J. H. Ryu N. E. Lim D. J. Park H. (2019). Gold nanoparticles for photothermal cancer therapy. Front. Chem. 7, 167. 10.3389/fchem.2019.00167 31024882 PMC6460051

[B302] Vu T. T. Stafford A. R. Leslie B. A. Kim P. Y. Fredenburgh J. C. Weitz J. I. (2013). Batroxobin binds fibrin with higher affinity and promotes clot expansion to a greater extent than thrombin. J. Biol. Chem. 288 (23), 16862–16871. 10.1074/jbc.M113.464750 23612970 PMC3675619

[B303] Wang H. Ke M. Tian Y. Wang J. Li B. Wang Y. (2013). BF-30 selectively inhibits melanoma cell proliferation via cytoplasmic membrane permeabilization and DNA-binding *in vitro* and in B16F10-bearing mice. Eur. J. Pharmacol. 707 (1-3), 1–10. 10.1016/j.ejphar.2013.03.028 23541725

[B304] Wang J. Qin X. Zhang Z. Chen M. Wang Y. Gao B. (2014). Crotoxin suppresses the tumorigenic properties and enhances the antitumor activity of Iressa® (gefinitib) in human lung adenocarcinoma SPCA-1 cells. Mol. Med. Rep. 10 (6), 3009–3014. 10.3892/mmr.2014.2620 25310019

[B305] Wang J. H. Xie Y. Wu J. C. Han R. Reid P. F. Qin Z. H. (2012). Crotoxin enhances the antitumor activity of gefinitib (Iressa) in SK-MES-1 human lung squamous carcinoma cells. Oncol. Rep. 27 (5), 1341–1347. 10.3892/or.2012.1677 22322185

[B306] Wang K. Yan J. Liu X. Zhang J. Chen R. Zhang B. (2011). Novel cytotoxity exhibition mode of polybia-CP, a novel antimicrobial peptide from the venom of the social wasp Polybia paulista. Toxicology 288 (1-3), 27–33. 10.1016/j.tox.2011.06.014 21745529

[B307] Wang K. R. Zhang B. Z. Zhang W. Yan J. X. Li J. Wang R. (2008). Antitumor effects, cell selectivity and structure-activity relationship of a novel antimicrobial peptide polybia-MPI. Peptides 29 (6), 963–968. 10.1016/j.peptides.2008.01.015 18328599

[B308] Wang Y. Li K. Han S. Tian Y. H. Hu P. C. Xu X. L. (2019). Chlorotoxin targets ERα/VASP signaling pathway to combat breast cancer. Cancer Med. 8 (4), 1679–1693. 10.1002/cam4.2019 30806044 PMC6488122

[B309] Wang Y. Wang J. Cao Z. Barati Farimani A. (2022). Molecular contrastive learning of representations via graph neural networks. Nat. Mach. Intell. 4, 279–287. 10.1038/s42256-022-00447-x

[B310] Wang Y. Zhang J. Jiang P. Li K. Sun Y. Huang Y. (2021). ASIC1a promotes acidic microenvironment-induced HCC cells migration and invasion by inducing autophagy. Eur. J. Pharmacol. 907, 174252. 10.1016/j.ejphar.2021.174252 34116040

[B311] Waqar M. Batool S. (2015). *In silico* analysis of binding of neurotoxic venom ligands with acetylcholinesterase for therapeutic use in treatment of Alzheimer's disease. J. Theor. Biol. 372, 107–117. 10.1016/j.jtbi.2015.02.028 25747777

[B312] Ward C. W. Sachs F. Bush E. D. Suchyna T. M. (2018). GsMTx4-D provides protection to the D2.mdx mouse. Neuromuscul. Disord. 28 (10), 868–877. 10.1016/j.nmd.2018.07.005 30174173 PMC6368066

[B313] Warkentin T. E. (2004). Bivalent direct thrombin inhibitors: hirudin and bivalirudin. Best. Pract. Res. Clin. Haematol. 17 (1), 105–125. 10.1016/j.beha.2004.02.002 15171961

[B314] Wattam B. Shang D. Rahman S. Egglezou S. Scully M. Kakkar V. (2001). Arg-Tyr-Asp (RYD) and Arg-Cys-Asp (RCD) motifs in dendroaspin promote selective inhibition of β1 and β3 integrins. Biochem. J. 356 (Pt 1), 11–17. 10.1042/0264-6021:3560011 11336631 PMC1221807

[B315] Wei B. Zhang Y. Gong X. (2022). DeepLPI: a novel deep learning-based model for protein-ligand interaction prediction for drug repurposing. Sci. Rep. 12 (1), 18200. 10.1038/s41598-022-23014-1 36307509 PMC9616420

[B316] Westerlund B. Nordlund P. Uhlin U. Eaker D. Eklund H. (1992). The three-dimensional structure of notexin, a presynaptic neurotoxic phospholipase A2 at 2.0 A resolution. FEBS Lett. 301 (2), 159–164. 10.1016/0014-5793(92)81238-h 1568473

[B317] WHO (2021). Snake antivenoms. Available at: http://www.who.int/mediacentre/factsheets/fs337/en/(Accessed August 22, 2023).

[B318] Winblad B. Jelic V. (2004). Long-term treatment of Alzheimer disease: efficacy and safety of acetylcholinesterase inhibitors. Alzheimer Dis. Assoc. Disord. 18 (Suppl. 1), S2–S8. 10.1097/01.wad.0000127495.10774.a4 15249842

[B319] Wong E. S. Morgenstern D. Mofiz E. Gombert S. Morris K. M. Temple-Smith P. (2012). Proteomics and deep sequencing comparison of seasonally active venom glands in the platypus reveals novel venom peptides and distinct expression profiles. Mol. Cell Proteomics 11 (11), 1354–1364. 10.1074/mcp.M112.017491 22899769 PMC3494181

[B320] World Spider Catalog (2023). World Spider Catalog. Version 25.5. Natural History Museum Bern, Available at: http://wsc.nmbe.ch. (Accessed October 21, 2023).

[B321] Wu M. Ming W. Tang Y. Zhou S. Kong T. Dong W. (2013). The anticancer effect of cytotoxin 1 from Naja atra Cantor venom is mediated by a lysosomal cell death pathway involving lysosomal membrane permeabilization and cathepsin B release. Am. J. Chin. Med. 41 (3), 643–663. 10.1142/S0192415X13500456 23711147

[B322] Wu W. Yin Y. Feng P. Chen G. Pan L. Gu P. (2023). Spider venom-derived peptide JZTX-14 prevents migration and invasion of breast cancer cells via inhibition of sodium channels. Front. Pharmacol. 14, 1067665. 10.3389/fphar.2023.1067665 37033662 PMC10076671

[B323] Wulff H. Castle N. A. Pardo L. A. (2009). Voltage-gated potassium channels as therapeutic targets. Nat. Rev. Drug Discov. 8 (12), 982–1001. 10.1038/nrd2983 19949402 PMC2790170

[B324] Xia Z. He D. Wu Y. Kwok H. F. Cao Z. (2023). Scorpion venom peptides: molecular diversity, structural characteristics, and therapeutic use from channelopathies to viral infections and cancers. Pharmacol. Res. 197, 106978. 10.1016/j.phrs.2023.106978 37923027

[B325] Xiao L. Gurrola G. B. Zhang J. Valdivia C. R. SanMartin M. Zamudio F. Z. (2016). Structure-function relationships of peptides forming the calcin family of ryanodine receptor ligands. J. Gen. Physiol. 147 (5), 375–394. 10.1085/jgp.201511499 27114612 PMC4845687

[B326] Xiong X. Menting J. G. Disotuar M. M. Smith N. A. Delaine C. A. Ghabash G. (2020). A structurally minimized yet fully active insulin based on cone-snail venom insulin principles. Nat. Struct. Mol. Biol. 27 (7), 615–624. 10.1038/s41594-020-0430-8 32483339 PMC7374640

[B327] Xiong Z. G. Zhu X. M. Chu X. P. Minami M. Hey J. Wei W. L. (2004). Neuroprotection in ischemia: blocking calcium-permeable acid-sensing ion channels. Cell 118 (6), 687–698. 10.1016/j.cell.2004.08.026 15369669

[B328] Yacoub T. Rima M. Karam M. Fajloun J. Fajloun Z. (2020). Antimicrobials from venomous animals: an overview. Molecules 25 (10), 2402. 10.3390/molecules25102402 32455792 PMC7287856

[B329] Yamada M. Miller D. M. Lowe M. Rowe C. Wood D. Soyer H. P. (2021). A first-in-human study of BLZ-100 (tozuleristide) demonstrates tolerability and safety in skin cancer patients. Contemp. Clin. Trials Commun. 23, 100830. 10.1016/j.conctc.2021.100830 34401600 PMC8355837

[B330] Yan C. H. Liang Z. Q. Gu Z. L. Yang Y. P. Reid P. Qin Z. H. (2006). Contributions of autophagic and apoptotic mechanisms to CrTX-induced death of K562 cells. Toxicon 47 (5), 521–530. 10.1016/j.toxicon.2006.01.010 16542694

[B331] Yan C. H. Yang Y. P. Qin Z. H. Gu Z. L. Reid P. Liang Z. Q. (2007). Autophagy is involved in cytotoxic effects of crotoxin in human breast cancer cell line MCF-7 cells. Acta Pharmacol. Sin. 28 (4), 540–548. 10.1111/j.1745-7254.2007.00530.x 17376294

[B332] Yang C. Zhu Z. Ouyang X. Yu R. Wang J. Ding G. (2020). Overexpression of acid-sensing ion channel 1a (ASIC1a) promotes breast cancer cell proliferation, migration and invasion. Transl. Cancer Res. 9 (12), 7519–7530. 10.21037/tcr-20-2115 35117352 PMC8799141

[B333] Ye M. Chung H. S. Lee C. Yoon M. S. Yu A. R. Kim J. S. (2016). Neuroprotective effects of bee venom phospholipase A2 in the 3xTg AD mouse model of Alzheimer's disease. J. Neuroinflammation 13, 10. 10.1186/s12974-016-0476-z 26772975 PMC4715334

[B334] Zamponi G. W. (2016). Targeting voltage-gated calcium channels in neurological and psychiatric diseases. Nat. Rev. Drug Discov. 15 (1), 19–34. 10.1038/nrd.2015.5 26542451

[B335] Zhang J. Tang D. Liu S. Hu H. Liang S. Tang C. (2018). Purification and characterization of JZTx-14, a potent antagonist of mammalian and prokaryotic voltage-gated sodium channels. Toxins (Basel) 10 (10), 408. 10.3390/toxins10100408 30308978 PMC6215091

[B336] Zhang J. Zhang K. Ren Y. Wei D. (2021). The expression, purification, and functional evaluation of the novel tumor suppressor fusion protein IL-24-CN. Appl. Microbiol. Biotechnol. 105 (20), 7889–7898. 10.1007/s00253-021-11558-7 34568963

[B337] Zhang P. Ma J. Yan Y. Chen B. Liu B. Jian C. (2017). Arginine modification of lycosin-I to improve inhibitory activity against cancer cells. Org. Biomol. Chem. 15 (44), 9379–9388. 10.1039/c7ob02233f 29090725

[B338] Zhang P. Yan Y. Wang J. Dong X. Zhang G. Zeng Y. (2020a). An anti-cancer peptide LVTX-8 inhibits the proliferation and migration of lung tumor cells by regulating causal genes' expression in p53-related pathways. Toxins (Basel) 12 (6), 367. 10.3390/toxins12060367 32498425 PMC7354478

[B339] Zhang Q. Zhang P. Jian S. Li J. Li F. Sun X. (2020b). Drug-bearing peptide-based nanospheres for the inhibition of metastasis and growth of cancer. Mol. Pharm. 17 (9), 3165–3176. 10.1021/acs.molpharmaceut.0c00118 32787278

[B340] Zhou Q. Hu P. Ritter M. R. Swenson S. D. Argounova S. Epstein A. L. (2000a). Molecular cloning and functional expression of contortrostatin, a homodimeric disintegrin from southern copperhead snake venom. Arch. Biochem. Biophys. 375 (2), 278–288. 10.1006/abbi.1999.1682 10700384

[B341] Zhou Q. Sherwin R. P. Parrish C. Richters V. Groshen S. G. Tsao-Wei D. (2000b). Contortrostatin, a dimeric disintegrin from *Agkistrodon contortrix* contortrix, inhibits breast cancer progression. Breast Cancer Res. Treat. 61 (3), 249–260. 10.1023/a:1006457903545 10966001

[B342] Zhu S. Darbon H. Dyason K. Verdonck F. Tytgat J. (2003). Evolutionary origin of inhibitor cystine knot peptides. FASEB J. 17 (12), 1765–1767. 10.1096/fj.02-1044fje 12958203

